# Life at the extremes: maximally divergent microbes with similar genomic signatures linked to extreme environments

**DOI:** 10.1093/nargab/lqaf189

**Published:** 2025-12-23

**Authors:** Monireh Safari, Joseph Butler, Gurjit S Randhawa, Kathleen A Hill, Lila Kari

**Affiliations:** School of Computer Science, University of Waterloo, Waterloo, N2L 3G1, Canada; Department of Biology, University of Western Ontario, London, N6A 3K7, Canada; School of Computer Science, University of Guelph, Guelph, N1G 2W1, Canada; Department of Biology, University of Western Ontario, London, N6A 3K7, Canada; School of Computer Science, University of Waterloo, Waterloo, N2L 3G1, Canada

## Abstract

Extreme environments impose strong mutation and selection pressures that drive distinctive, yet understudied, genomic adaptations in extremophiles. In this study, we identify 15 bacterium–archaeon pairs that exhibit highly similar $k$-mer-based genomic signatures despite maximal taxonomic divergence, suggesting that shared environmental conditions can produce convergent, genome-wide sequence patterns that transcend evolutionary distance. To uncover these patterns, we developed a computational pipeline to select a composite genome proxy assembled from noncontiguous subsequences of the genome. Using supervised machine learning on a curated dataset of 693 extremophile microbial genomes, we found that 6-mers and 100 kbp genome proxy lengths provide the best balance between classification accuracy and computational efficiency. Our results provide conclusive evidence of the pervasive nature of $k$-mer-based patterns across the genome, and uncover the presence of taxonomic and environmental components that persist across all regions of the genome. The 15 bacterium–archaeon pairs identified by our method as having similar genomic signatures were validated through multiple independent analyses, including 3-mer frequency profile comparisons, phenotypic trait similarity, and geographic co-occurrence data. These complementary validations confirmed that extreme environmental pressures can override traditionally recognized taxonomic components at the whole-genome level. Together, these findings reveal that adaptation to extreme conditions can carry robust, taxonomic domain-spanning imprints on microbial genomes, offering new insight into the relationship between environmental impacts and genome sequence composition convergence.

## Graphical Abstract

**Graphical Abstract:**
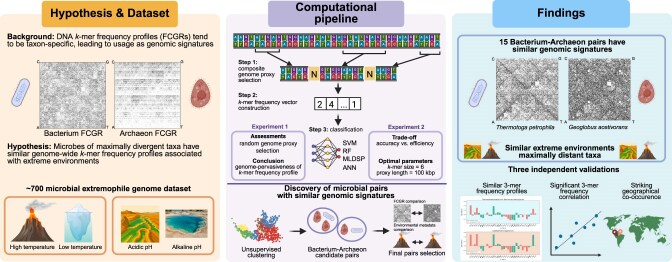

## Introduction

The study of extremophiles, organisms capable of thriving in Earth’s most extreme environments, provides crucial insights into genetic adaptations for survival under harsh conditions. These organisms have evolved to withstand extreme environmental conditions such as high temperature, pH, pressure, salinity, and radiation levels and, notably, they are often unable to survive outside of these physiologically extreme environmental conditions [1, 2]. Recent attention to microbial extremophiles has highlighted their value across diverse applications, including biotechnology, particularly in biorefineries and as sources of industrial extremozymes for high-temperature or high-pH processes [3–7], environmental bioremediation of metal-contaminated, saline, or radioactive environments [6], as well as agriculture and soil enhancement [8], and veterinary medicine [9]. Additionally, interest has grown in the ability of microbial extremophiles to survive the extreme conditions of outer space [10, 11].

Extremophiles have evolved specific genomic and proteomic adaptations to survive in environments with extreme conditions [12, 13]. At the genomic level, they frequently exhibit gene duplications [14, 15] and reduced genome sizes, particularly in thermophilic species [16]. The nucleotide composition of these organisms displays environment-specific patterns in G+C content and purine load [15, 17, 18]. Multiple molecular mechanisms, including gene duplication and horizontal gene transfer [19–21], also contribute to the genomic adaptation of extremophiles. Recent research has further emphasized the critical role of genomic regulatory elements in these environmental adaptations [6, 22, 23], as well as efficient DNA repair systems in extremophiles exposed to high radiation [7]. At the proteomic level, features such as codon usage bias and amino acid composition have been linked to thermal adaptation and have recently been used in machine learning models to predict optimal growth temperature [24].

A recent study applied supervised and unsupervised machine learning algorithms to explore the genomes of microbial extremophiles, uncovering both taxonomic and environmental components embedded within their genomic signatures [25]. In this approach, genomic signatures were derived from 500 kbp (contiguous) representative DNA fragments randomly selected from each genome, by computing the $k$-mer frequency vector of each fragment. Here, a $k$-mer is a DNA sequence of length $k$, and the $k$-mer frequency vector of a DNA fragment is a numerical vector comprising the counts of the occurrences of all possible $k$-mers in that fragment (in lexicographic order). These vectors enabled highly accurate classification and clustering tasks that revealed environmental components for extremophiles inhabiting environments with extreme temperature and/or pH conditions. While this approach provided valuable insights, it had some notable limitations. Firstly, the selection of the DNA fragment selected to act as a genome proxy was not entirely random (see the ‘Selecting a genome proxy’ section), which could potentially introduce bias in the derived genomic signatures. Secondly, the study did not quantitatively test the hypothesis of the pervasiveness of the environmental components across the entire genome.

This paper addresses these limitations by first refining the process of selecting a genomic signature to enhance the accuracy of organism classification based on genomic data. We focus on three key areas: (i) improving the genome coverage and randomness of the representative DNA fragment by replacing it with a *composite genome proxy* constructed through the pseudo-concatenation of several randomly selected (noncontiguous) DNA fragments, (ii) comprehensively testing the hypothesis of the pervasiveness of taxonomic and environmental components across a genome, and (iii) analyzing the impact of varying $k$-mer sizes and composite genome proxy lengths, ranging from 10 kbp to entire genomes. Through a series of computational experiments involving several genome proxy selection methods, $k$-mer sizes, and fragment lengths, we aimed to identify the optimal parameters for extremophile genomic signature analysis. The results of these analyses were then used to design a multilayered pipeline that identified multiple bacterium–archaeon pairs with similar genomic signatures in spite of their maximal taxonomic divergence, potentially due to the shared characteristics of their extreme environments. The main contributions of this paper are:

1. Conclusive evidence of the pervasiveness of a $k$-mer-based genomic signature throughout an extremophile genome.
2. A broadly applicable method for the selection of a composite genome proxy (hereafter referred to simply as ‘genome proxy’) assembled from noncontiguous subsequences of the genome. Empirical determination of the optimal $k$-mer size $(k=6$) and genome proxy length (100 000 bp) for fast and accurate taxonomic and environment-type classifications.
3. Discovery of 15 maximally divergent bacterium–archaeon pairs with similar genomic signatures linked to the characteristics of their extreme environment, through a multilayered filtering process used in conjunction with unsupervised machine learning.
4. Validation of the above computational findings through additional analyses, including 3-mer frequency profile analyses demonstrating agreement with known adaptative patterns in extremophiles, statistical confirmation of 3-mer frequency profiles similarity of the identified pairs using Spearman’s rank correlation analysis [26], and analysis of geographic co-occurrence data confirming that identified bacterium–archaeon pairs naturally co-occur in the same extreme environments.

## Materials and methods

This section outlines the methodology used in the computational experiments for this study. It includes a description of the genome sequence datasets and an explanation of how genomic signatures were calculated. The methodology also details the selection of a suitable genome proxy for taxonomic and environment-type machine learning classifications, including the empirical optimization of both the $k$-mer value and proxy length. Finally, it describes the multilayer approach used to identify bacterium–archaeon pairs that exhibit similar genomic signatures despite belonging to highly divergent taxa, with the goal of uncovering shared genomic characteristics associated with extreme environments.

### Dataset

In the quest to evaluate genome-wide genomic signatures potentially shaped by similar extreme environments for maximally divergent microbes, it is essential to determine the optimal genome proxy to represent each genome. This selection is crucial for ensuring accurate $k$-mer-based classification and clustering.

For this reason, and in order to be able to perform apples-to-apples comparisons with existing results, we utilized the dataset from [25]. The dataset consists of 693 high-quality extremophile microbial genome assemblies curated via a comprehensive review of primary literature and cross-referenced with the Genome Taxonomy Database [27]. These microbial genomes were grouped into two different environment-type datasets, one based on the organisms’ optimal growth temperature (psychrophiles, mesophiles, thermophiles, hyperthermophiles; see Table 1) and the other based on their optimal growth pH levels (acidophiles and alkaliphiles; see Table 2).

**Table 1.** The ‘Temperature dataset’: taxonomic diversity of archaea and bacteria across temperature categories

Domain	Temperature category	# Phyla	# Classes	# Orders	# Families	# Genera	# Species
Archaea	Psychrophiles	2	4	4	5	7	8
	Mesophiles	4	6	7	20	45	84
	Thermophiles	6	11	14	21	41	67
	Hyperthermophiles	5	6	8	15	31	70
Bacteria	Psychrophiles	4	4	6	13	19	140
	Mesophiles	3	3	6	10	14	106
	Thermophiles	15	19	24	27	47	116
	Hyperthermophiles	5	5	5	5	5	7

The four temperature categories are defined, based on the optimal temperature for growth (OTG). These categories are as follows: Psychrophiles (OTG of <20°C), Mesophiles (OTG of 20–45°C), Thermophiles (OTG of 45–80°C), and Hyperthermophiles (OTG of >80°C) [25].

**Table 2.** The ‘pH dataset’: taxonomic diversity of archaea and bacteria across pH categories

Domain	pH Category	# Phyla	# Classes	# Orders	# Families	# Genera	# Species
Archaea	Acidophiles	4	5	7	11	24	39
	Alkaliphiles	2	5	5	9	18	30
Bacteria	Acidophiles	10	12	13	13	32	61
	Alkaliphiles	12	14	25	30	36	56

The two pH categories are defined based on the optimal growth pH (OGpH). These are acidophiles (OGpH < pH 5) and alkaliphiles (OGpH > pH 9) [25].

The first dataset, called the ‘Temperature dataset’, is composed of 598 genomes including 148 psychrophile genomes, 190 mesophile genomes, 183 thermophile genomes, and 77 hyperthermophile genomes. The second dataset, called the ‘pH dataset’, is composed of 186 genomes, including 100 acidophile genomes and 86 alkaliphile genomes. There are 91 genomes that are present in both datasets, falling into one of two categories: mesophiles that live in acidic or alkaline environments (8 genomes), and polyextremophiles, that thrive in environments that are both acidic/alkaline and at extreme temperatures (83 genomes). The details of the samples that are in both datasets can be found in the Supplementary Materials, Section A.

### Genomic signature

Due to the massive lengths of genomic sequences and the high computational demands of alignment-based methods [28], researchers are now using alignment-free methods leveraging ‘genomic signatures’ for efficient genome classification or clustering. The genomic signature of an organism is typically represented by a $k$-mer frequency vector [29], derived from the entire genome or a ‘sufficiently long’ DNA fragment that captures the pervasiveness of the signature [30]. These signatures have proven effective in differentiating species and have been applied in various contexts, including microbial diversity analysis [31–35, 36], classification or subtyping of viral genomes [33, 37–41], and metagenomic classification and profiling [35, 42].

**Figure 1.**
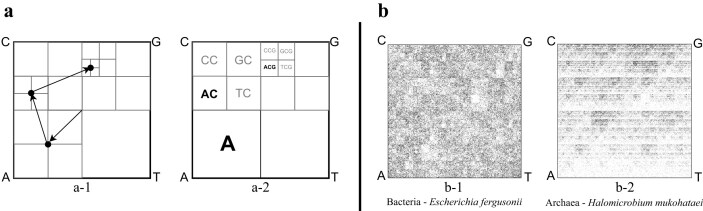
*Left:* CGR of the DNA sequence ‘ACG’. (**a-1**) The CGR is generated within a square with corners labeled A, C, G, T. The plot is generated by reading the sequence from left to right, and iteratively plotting the midpoint between the current point and the corner labeled by the nucleotide being read (the start point is the square’s center). For example, the sequence ACG consists of three points generated in the order illustrated by the arrows. (**a-2**) The resulting CGR, where the square regions labeled by **A, AC**, and **ACG** are the regions where the $k$-mers A, AC, ACG would be plotted, regardless of their position in the sequence. *Right:* FCGRs of randomly selected 100 kbp genomic fragments belonging to organisms from two different domains of life, (**b-1**) *Escherichia fergusonii* (bacterium) and (**b-2**) *Halomicrobium mukohataei* (archaeon). Both species live in similar habitats with moderate temperatures (optimal growth temperature of $20-45^{\circ }$C). The value of $k$ is 8, and thus each image is a $256 \times 256$ grayscale grid ($2^8 = 256$), where each pixel represents the frequency of a specific 8-mer in the DNA fragment. Darker (lighter) pixels indicate higher (lower) numbers of occurrences of the corresponding 8-mers in the respective sequences. The unique patterns in each image reflect the genome sequence composition for that species.

Chaos Game Representation (CGR) of DNA sequences, first introduced by Jeffrey in 1990 [43], has emerged as a particularly effective method for calculating and visualizing genomic signatures. Figure 1 (left panel) provides a brief illustration of the process of generating the CGR of the sample DNA sequence ‘ACG’. A quantified version of CGR, called Frequency Chaos Game Representation (FCGR) [44], produces a $2^k \times 2^k$ grayscale image, where the pixel intensities correspond to $k$-mer frequencies. The patterns in an FCGR of a genomic sequence reflect its composition, and several studies have demonstrated the effectiveness of FCGR images in taxonomic classification at various taxonomic levels [45–47]. As expected, the FCGRs of an archaeon and a bacterial species are visually different, which is consistent with the genetic difference anticipated for species of two different domains of life (Fig. 1, right panel).

The capability of genomic signatures to differentiate between organisms across taxonomic levels, combined with the visualization power of FCGRs, makes genomic signatures particularly suitable for the analysis of extremophiles, where we seek to understand how environmental adaptations might influence genomic signatures across different taxa. In this study, FCGR is employed for the quantitative evaluation of candidate bacterium–archaeon pairs (see the ‘FCGR comparison of candidate pairs’ section), while $k$-mer frequency vectors serve as the primary genomic signature throughout all classification and clustering analyses.

### Selecting the genome proxy, and empirically optimizing the *k*-mer value and genome proxy length

This section begins by proposing a new procedure for the selection of a genome proxy. Using this selection method, along with supervised learning methods applied to the ‘Temperature dataset’ and ‘pH dataset’, we then assessed the effect of genome proxy selection on classification accuracy, and empirically determined the optimal values for $k$-mer size and representative genome proxy length.

#### Selecting a genome proxy

While genomic signatures have been shown to be effective for classification and clustering of genomic sequences, the validity and accuracy of such analysis highly depend on the selection of representative DNA fragments capable of serving as a genome proxy. The approach utilized in [25] had notable limitations that our current methodology aims to address. Specifically, the selection process was not completely random, since the selected representative was a contiguous long fragment of the genome and the selection process prioritized longer contigs over shorter ones. Recall that the selection process in [25] starts from the list of contigs sorted in decreasing order of their length. If the longest contig exceeded 500 kbp in length, a 500 kbp subfragment was randomly chosen from that contig as the representative DNA fragment of that genome. Otherwise, the contigs were pseudo-concatenated one after another, until the pseudo-concatenated sequence reached a length of 500 kbp, and this sequence was taken to be the representative DNA fragment of that genome. Here, the *pseudo-concatenation* of DNA sequences is defined as listing them one after another, with a separator letter ‘N’ between every two consecutive sequences. Pseudo-concatenation prevents the formation of spurious $k$-mers during the process, and $k$-mers containing the letter ‘N’ are not counted when computing the $k$-mer frequency vector of the pseudo-concatenated sequence, with ‘N’ not being counted towards the pseudo-concatenated sequence length. One potential limitation of this selection process is that it biases the choice towards representative DNA fragments extracted from longer contigs. Another potential limitation is that, if the first contig is sufficiently large, the representative fragment will be selected from a single region of the genome. These limitations introduce a bias in the selection of the representative DNA fragment, which presupposed a genome-wide pervasive nature of a genomic signature and could potentially affect the classification accuracy.

To address these limitations, we propose a procedure for selecting a composite genome proxy that ensures that all fragments in the genome have an equal probability of being included in the final genome proxy. In addition, this procedure ensures that the final genome proxy includes multiple sequences from various locations in the genome. The method of selecting a genome proxy has three steps:

1. Empirically determining the optimal values for $n$ (the number of nonoverlapping genomic subfragments that comprise a genome proxy $s$), and for $len(s)$, the total length of the genome proxy $s$.
2. Pseudo-concatenating all contigs into a single large sequence, if the genome sequence is composed of multiple contigs;
3. Pseudo-concatenating $n$ different, randomly selected, nonoverlapping subfragments of length $len(s)/n$ from either the genome (if it consists of a single contig), or from the pseudo-concatenated sequence obtained in the preceding step (if the genome consists of multiple contigs).

**Figure 2.**
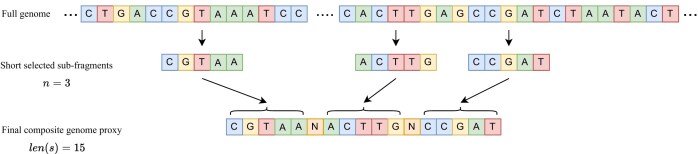
The selection process of a genome proxy $s$, comprising $n=3$ nonoverlapping sub-fragments, and with total length $len(s) = 15$. Top: Full genome, consisting of only one contig. Middle: $n$ nonoverlapping sub-fragments (here $n = 3$) randomly selected from the genome. Bottom: The genome proxy of length $ len(s) = 15$ obtained by pseudo-concatenating the sub-fragments.

Figure 2 illustrates the selection process of a genome proxy when the genome consists of only one contig, $n = 3$, and $len(s) = 15$.

#### Determining the optimal *k*-mer size and genome proxy length

After selecting the genome proxy in a way that ensured randomness, we evaluated the impact of the following three factors on the supervised classifier accuracies (i) the choice of genome proxy, (ii) $k$-mer size, and (iii) genome proxy length. The feature vectors used as an input for these classifiers were *canonical $k$-mer* frequency vectors. Here, a ‘canonical $k$-mer’ is defined as the first, in alphabetical order, of a $k$-mer and its Watson–Crick complement. For any DNA sequence, the final frequency vector was computed by averaging the $k$-mer counts of the sequence with $k$-mer counts of its Watson–Crick reverse complement [25]. In the remainder of this paper, only the canonical $k$-mer will be listed.

The classifier used to evaluate the impact of factor (i) was the support vector machine (SVM) with a Radial Basis Function (RBF) kernel, which has been shown to achieve high accuracies in genome sequence classification across various fields [25, 48, 49]. To investigate the impact of factors (ii) and (iii), we expanded our analysis to include six classifiers: SVM with RBF kernel, Random Forest with 100 estimators, and an Artificial Neural Network (ANN) with two hidden layers (sizes 256 and 64, and a learning rate of 0.001). Additionally, three variations of the Machine Learning with Digital Signal Processing (MLDSP) algorithm (MLDSP-1, MLDSP-2, and MLDSP-3) [33] were also included. Other deep learning classifiers, such as CNNs, were not considered because the number of samples in our dataset was insufficient to reliably train such models.

In these computational experiments, we explored nine $k$-mer sizes ranging from 1 to 9, to find the optimal value of $k$. Larger $k$-mer sizes were avoided to prevent sparsity in the feature vector, which can undermine classification accuracy. Six genome proxy lengths ($len(s)$) were evaluated: 10, 50, 100, 250, 500, and 1000 kbp. The fragment lengths were chosen to include both short sequences (e.g. 10 kbp, ∼3% of the average sequence length in our dataset) and longer sequences, allowing for a comprehensive comparison while also considering computational costs. For all experiments, the number of sub-fragments ($n$) comprising the sequence $s$ was set to 10, a value empirically determined as optimal for the datasets used. This choice is supported by existing studies indicating that the minimum sequence length necessary to capture genomic patterns is of the order of $10^3$ [43], making $n = 10$ an effective choice for ensuring that each sub-fragment independently captures the relevant genomic patterns, particularly in the case of shorter fragments.

In the experiment for evaluating the impact of factor (i), we repeated the following process 10 times: First, a random genome proxy was selected for each sequence. Then, for each combination of $k$-mer size and fragment length ($9 \times 6$ combinations), we performed classification using an SVM with 10-fold cross-validation. To assess the performance of the classification, the accuracy was defined as the ratio of the number of sequences with correctly predicted labels to the total number of sequences classified. The variance of the classification accuracy over these 10 runs of the classification was then calculated to determine if the accuracy was dependent on the choice of random genome proxy.

Following the observation that classification accuracy is not dependent on the choice of genome proxy (see the ‘Genome proxy’ section for details), finding the optimal $k$-mer size [factor (ii)], and finding the optimal fragment length [factor (iii)], were carried out by running classification experiments using the six classifiers with all combinations of $k$-mer sizes and fragment lengths. Note that, for each fragment length, a fixed randomly selected genome proxy was utilized.

In addition, a separate experiment was performed using the full DNA genome (maximal sequence length) and the determined optimal $k$-mer size, to evaluate the impact of considering the information from the whole genome on classification accuracy, as opposed to a random shorter fragment.

The computational experiments were performed for the two different datasets, the ‘Temperature dataset’ and the ‘pH dataset’. All classifications were conducted under two distinct supervised training scenarios: genome proxies labeled with taxonomic labels (bacteria or archaea), and genome proxies labeled with environment-type labels (for the ‘Temperature dataset’, psychrophiles, mesophiles, thermophiles, and hyperthermophiles; for the ‘pH dataset’, acidophiles and alkaliphiles).

Taxonomic analyses employed stratified 10-fold cross-validation. For environment-type classifications, tests were conducted under two scenarios: a ‘standard’ scenario and a ‘bias mitigation’ scenario.

In the standard scenario, conventional stratified 10-fold cross-validation was applied, and the average classification accuracy was reported across the ten folds. The bias mitigation scenario was designed to separate genus-level taxonomic signals from environment-specific genomic patterns. Here, folds were constructed so that all sequences from the same genus were placed in the same fold, while the distribution of all labels in each fold remained the same as in the entire dataset. The fact that sequences from the same genus were not split between folds ensured that the environment-type labels of test sequences were predicted due to their environment-specific similarities, rather than due to genus-specific similarities with sequences in the training set.

### Finding bacterium–archaeon pairs with similar genomic signatures, linked to their extreme environments

Once the performance of the optimal parameters was validated, the main objective of this study was to identify, if any, microbe pairs from two different taxonomic domains (archaea and bacteria) that shared similarities in their genomic signatures that were linked to their shared extreme environment types.

A multilayered approach was used to identify bacterium–archaeon pairs of sequences with similar genomic signatures. The first layer involved the generation of ‘candidate bacterium–archaeon pairs’, i.e. maximally different microbe pairs clustered together by nonparametric unsupervised clustering machine learning algorithms. To eliminate potential clustering algorithm errors, this candidate pair list was subjected to a second selection layer, comprising a quantitative comparison of the FCGRs of members of each candidate pair, which resulted in pairs with similar genomic signatures called ‘confirmed candidate pairs’. Finally, we used the confirmed candidate pairs to test the hypothesis that genomic similarities of pair members were due to shared environmental pressures, by exploring the isolating environment metadata of the members of each pair, resulting in a list of ‘environment-related pairs’.

We then further investigated the environment-related pairs by analyzing the 3-mer frequency profiles of the pair members and corroborating the results with biological findings of over- and under-representation of codons in extremophile microbes, as well as by exploring the geographic habitat co-occurrence of pair members.

#### Nonparametric clustering

In this section, our primary objective was to identify pairs of archaea and bacteria (if any) that clustered together based on similar genomic signatures, despite their maximal taxonomic divergence. To achieve this, we first sought to determine clustering methods that could reliably reproduce known taxonomic relationships at the genus level (the lowest taxonomic level in our datasets). These validated clustering algorithms were then applied to identify exceptional cross-domain clustering cases. The rationale behind this approach is that if a clustering algorithm could successfully group sequences by genus, then any instance where it grouped bacteria and archaea together was more likely to reflect a true cross-domain genomic signature similarity rather than being a computational artifact. To this end, only nonparametric unsupervised clustering algorithms were used, since they have the advantage of not needing the expected number of clusters as an input parameter. Specifically, the five algorithms used were the nonparametric version of the $i$DeLUCS algorithm [31], and four other nonparametric algorithms (HDBSCAN [50], Affinity Propagation [51], MeanShift [52], and iterative medoids [53]). These algorithms were applied in conjunction with two different dimensionality reduction techniques, variational autoencoders (VAE) [53], and uniform manifold approximation and projection (UMAP) [54].

We tested different combinations of dimensionality reduction techniques and clustering algorithms to find those that best reproduced clusters matching true genera in our datasets. Their performance was measured using completeness and contamination. Completeness refers to the proportion of true members within a cluster (cluster members belonging to the same genus) relative to the total cluster size, and contamination indicates the proportion of incorrect members (cluster members that belong to a different genus) relative to the total cluster size.

Only those clusters were accepted as ‘genus-accurate’ that had completeness >50% and contamination <50%. The next step was to rank the aforementioned combinations by the ratio of the number of genus-accurate clusters to the total number of generated clusters. The top five combinations were selected, namely: VAE+iterative medoids (IM), VAE+ Affinity Propagation, VAE+HDBSCAN, UMAP+HDBSCAN, and $i$DeLUCS.

In the final step, we used all output clusters from the selected top five combinations to identify pairs of archaea and bacteria that clustered together. Specifically, for each of the top five combinations, we ran the clustering process 10 times, each time with a different random seed, each time producing the pairs of maximally divergent microbes that were clustered together. From the resulting set of pairs, the pairs that appeared in >5 runs, and were clustered together by the majority of the five combinations, were retained, as being ‘candidate bacterium–archaeon pairs’, subjected to the next layer of analysis.

#### FCGR comparison of candidate pairs

To address the errors inherent in any unsupervised clustering method, we then analyzed the candidate bacterium–archaeon pairs identified in the pervious section by investigating the similarities of the FCGR patterns of the members of each candidate pair. For this analysis, FCGR images of candidate pairs were generated from the selected genome proxy using the optimal $k$-mer size determined previously. Subsequently, three distinct distance metrics were used, Descriptor [55], structural dissimilarity index measure (DSSIM) [56], and learned perceptual image patch similarity (LPIPS) [57] to calculate the distances between each pair of candidate bacterium–archaeon pairs that were clustered together. The refined set of candidate pairs was selected based on the similarity of their FCGR images. Specifically, pairs were selected if the distance between their FCGRs was below certain distance-dependent thresholds for all three distance metrics. The distance-dependent thresholds were 0.190211 for the Descriptor distance, 0.501385 for DSSIM, and 0.177668 for LPIPS, and were empirically determined as detailed below.

The thresholds for the distance metrics were determined based on the idea that two members of a bacterium–archaeon pair will be considered similar if their FCGR distance is less than the distance among FCGRs of species of the same genus. To this end, the intra-genus distance in the dataset was computed as follows. First, we selected all unique genera from both the ‘Temperature dataset’ and ‘pH dataset’, excluding those with only a single sample, which resulted in 92 unique genera. Then, for each genus, the pairwise distances between the FCGRs of all sequences were calculated. The average of these distances within each genus was deemed to be the intra-genus distance for that genus. Of the obtained intra-genus distances, 10% of the distances were excluded as outliers (the top and bottom 5%). In the final step, the 90th percentile of these average intra-genus distances was considered as the empirical threshold for FCGR comparison for that distance. More details of intra-genus distance computations can be found in Supplementary Materials, Section B. This approach ensured that the identified microbial pairs clustered together based on genomic signature similarity in the previous layer, and also exhibited significant similarities in their FCGR patterns. The output of this layer was a list of ‘confirmed candidate pairs’.

#### Hypothesis testing using isolating environment metadata of confirmed candidate pairs

After identifying confirmed candidate pairs with similar genomic signatures, we explored the hypothesis that this similarity was environment-related. To do so, we examined the environmental type of the habitats where the members of each pair were isolated. This process involved comparing the environmental labels assigned to each species within a pair (e.g. temperature and pH). Microbial pairs with matching (implying the same temperature and/or pH labels, i.e. both species are acidophiles) or nearly matching (similar temperature and/or pH labels, i.e. both species inhabit high-temperature environments, though one is thermophilic and the other is hyperthermophilic) environmental labels were considered to be ‘environment-related pairs’ and were retained for further analysis. We also conducted a more detailed analysis, where we retrieved the original studies that first characterized these microbes from PubMed (https://pubmed.ncbi.nlm.nih.gov/). The growth parameters and environmental metadata, such as optimal pH and temperature ranges, were compared across species. Additionally, we examined phenotypic traits and habitat-specific characteristics to gain a deeper understanding of shared environmental adaptations and similar phenotype features of the pairs.

#### Analysis of 3-mer frequency profiles of environment-related bacterium–archaeon pairs

Following the refinement steps, we conducted a comprehensive 3-mer usage bias analysis by comparing the 3-mer frequency profiles of the environment-related pairs. We selected $k=3$ for this analysis because this $k$-mer length effectively captures codon usage bias, amino acid bias, and protein-associated phenotypic adaptations [25, 58, 59]. Our analysis consisted of four main components. First, for each 3-mer, we calculated its average frequency across all samples in the ‘Temperature dataset’ and ‘pH dataset’, then calculated the deviation of the 3-mer frequency of each member of the environment-related pairs from its dataset average. This approach revealed patterns of similar 3-mer over- and under-representation in pair members compared to the entire dataset, allowing us to investigate whether similar environmental conditions induced comparable patterns of 3-mer usage across microbial pairs. Second, we tested the correlation between the 3-mer counts of members of the confirmed pair in each group using Spearman’s rank correlation coefficient, a nonparametric measure of the strength and direction of association between two variables measured on an ordinal scale [26, 39, 60–62]. This step investigated the pairwise correlation of 3-mer representation among confirmed pairs, providing a *P*-value to assess the significance of similarity or dissimilarity in the 3-mer over- and under-representation.

Third, we identified the specific 3-mers that influenced environmental label prediction in supervised classification for each microbial species in the environment-related pairs. We used the SHapley Additive exPlanations (SHAP) [63] feature importance method to quantify each 3-mer’s contribution to the model’s environmental classification decisions. SHAP is a model-agnostic explainability method that assigns importance values to individual features based on their marginal contributions to the prediction outcome. Specifically, SHAP quantifies how much each 3-mer frequency increases or decreases the probability of correctly classifying the environment-type relative to the baseline (average) prediction. We referred to these 3-mers as ‘environment-relevant 3-mers’ due to their impact on the model’s ability to distinguish between sequences belonging to organisms living in different environmental conditions.

Finally, we treated the ‘environment-relevant 3-mers’ as quasi-codons and translated them to corresponding amino acids [64]. This translation step enabled direct comparisons between the environment-relevant 3-mers discovered by our method and both codon and amino acid usage biases previously reported in the literature for the respective extremophilic groups. This comparative analysis serves to validate our methodology by demonstrating that the 3-mers we identified as important for environmental-based classification align with known adaptive patterns in extremophiles reported in the literature [25].

#### Geographic habitat co-occurrence analysis of environment-related pairs

In this analysis, the Microbe Atlas Project (MAP) database [65], cataloging 16S ribosomal RNA (rRNA) reads of microbes isolated from a wide range of environments, was used to analyze the geographic habitat co-occurrence of species in environment-related pairs. 16S rRNA is a gene encoding a ribosomal subunit highly conserved between different prokaryotes (including bacteria and archaea) [66]. The sequencing of this gene permits highly sensitive taxonomic classification/identification of prokaryotic samples, proving extremely helpful in identifying species found in diverse microbiomes. The MAP tool compiles millions of samples isolated across the world, along with their taxonomic classifications down to the species level, and geographic metadata (including coordinate information) associated with the sample collection site. The MAP was thus employed to identify the location data of 16S rRNA read occurrences of each species in the environment-related pairs list. After identifying the 16S rRNA sample reads cataloged for a particular species, the read locations, along with project and sample IDs (linking to project descriptions on the MAP database, which further characterize the geographic metadata), were exported to a spreadsheet. In the next step, the project IDs associated with the reads of each species within each respective group were cross-referenced to identify samples isolated from the same project ID (i.e. the same geographic location or microbiome). The projects found to contain 16S rRNA reads for each of the species within the final groups were identified via their respective ID in the MAP tool. Finally, environmental metadata, including environmental descriptors and longitude and latitude coordinates for each particular read, were identified. Through this process, we investigated the geographic habitat co-occurrence (referred to simply as ‘co-occurrence’ throughout the remainder of the paper) of the pairs of environment-related pairs, as well as descriptions of the unique environments that organisms in these groups inhabit.

## Results

In the following section, we first present the results of assessing the effect of randomly selecting a genome proxy on classification accuracy. We then detail the findings from the second experiment, focusing on the optimal values for $k$-mer size and genome proxy length, as well as the supervised classification accuracy achieved using these optimal parameters. Finally, we describe the candidate bacterium–archaeon pairs identified through nonparametric methods, the results of subsequent filtering steps, the confirmed set of bacterium–archaeon pairs, the analysis of 3-mer frequency profiles in these pairs, and the results of co-occurrence analysis for the confirmed bacterium–archaeon pairs.

### Genome proxy

As described in the ‘Materials and methods’ section, we conducted an experiment to assess the impact of a randomly selected genome proxy on taxonomic and environment-type classification under two different scenarios: the bias mitigation scenario and the standard scenario. For each scenario, we used 10-fold cross-validation classification with SVM classifier and repeated the classification process 10 times for each genome proxy length. To evaluate the results, we calculated the average accuracy and variance over the 10 runs for each genome proxy length. The results for the bias mitigation scenario are summarized in Table 3 (‘Temperature dataset’) and Table 4 (‘pH dataset’). For each tested genome proxy length, we reported the maximum average accuracy across the $k$-mer values and the value of $k$ for which it was obtained. The results for the standard scenario are similar and can be found in the Supplementary Materials, Section C.

**Table 3.** Maximum average accuracy across six genome proxy lengths in ten repeated SVM classification trials on the ‘Temperature dataset’ under the bias mitigation scenario, for $k$-mer sizes 1–9

Genome proxy length	Class labeling type	Max avg accuracy (%)	Variance (%)	$k$ -value
10 kbp	Taxonomy	98.35	0.0010	5
	Temperature	67.39	0.0267	6
50 kbp	Taxonomy	99.03	0.0001	6
	Temperature	72.36	0.0224	7
100 kbp	Taxonomy	99.13	0.0000	6
	Temperature	73.18	0.0209	7
250 kbp	Taxonomy	99.15	0.0000	6
	Temperature	75.31	0.0035	9
500 kbp	Taxonomy	99.15	0.0000	6
	Temperature	76.97	0.0035	9
1000 kbp	Taxonomy	99.15	0.0000	6
	Temperature	76.91	0.0013	9

The table lists the highest average accuracy for each genome proxy length, alongside the $k$-mer size that achieved this accuracy and the variance in percentage. The ‘Temperature dataset’ has 598 samples, consisting of 369 bacteria and 229 archaea. There are 148 psychrophiles, 190 mesophiles, 183 thermophiles, and 77 hyperthermophiles in this dataset.

**Table 4.** Maximum average accuracy across six genome proxy lengths in ten repeated SVM classification trials on the ‘pH dataset’ under the bias mitigation scenario, for $k$-mer sizes 1–9

Genome proxy length	Class labeling type	Max avg accuracy (%)	Variance (%)	$k$ -value
10 kbp	Taxonomy	97.18	0.0041	5
	pH	83.17	0.0469	6
50 kbp	Taxonomy	98.25	0.0022	7
	pH	84.89	0.0040	7
100 kbp	Taxonomy	98.63	0.0006	7
	pH	85.61	0.0092	8
250 kbp	Taxonomy	98.62	0.0012	8
	pH	85.41	0.0053	9
500 kbp	Taxonomy	98.94	0.0000	9
	pH	86.20	0.0030	9
1000 kbp	Taxonomy	98.94	0.0000	9
	pH	85.74	0.0035	9

The table lists the highest average accuracy for each genome proxy length alongside the $k$-mer size that achieved this accuracy and the variance in percentage. The ‘pH dataset’ has 186 samples, consisting of 117 bacteria and 69 archaea. There are 100 acidophiles and 86 alkaliphiles in this dataset.

In spite of the fact that each experiment was repeated 10 times, each time using a different randomly selected genome proxy, the maximum average accuracies are consistently high for taxonomy classifications and medium-high for environment-type classifications, with low variance across 10 different runs. These results support the hypothesis that the genomic signature, herein defined as the $k$-mer frequency vector of a short genomic fragment, is pervasive across the genome. Overall, these results indicate that selecting and pseudo-concatenating random regions of the genome into a contiguous genome proxy does not affect the taxonomic and environment-type classification accuracy, and is thus a valid selection method for these purposes.

The notable difference in environment-type classification accuracy between the two datasets can be partially attributed to the complexity of the classification task. Indeed, the ‘Temperature dataset’ has four unique labels while the ‘pH dataset’ has only two, making the latter an inherently simpler classification task.

### Optimal *k*-mer size and genome proxy length

The aim of this experiment is to identify the optimal $k$-mer size and the optimal genome proxy length for the purpose of taxonomy and environment-type classifications. To achieve this, we began by first determining the optimal $k$-mer size and then proceeded to determine the optimal genome proxy length. Our approach, especially when analyzing the various $k$-mer sizes, was to find a balance between computational time complexity/memory usage and classification accuracy.

**Figure 3.**
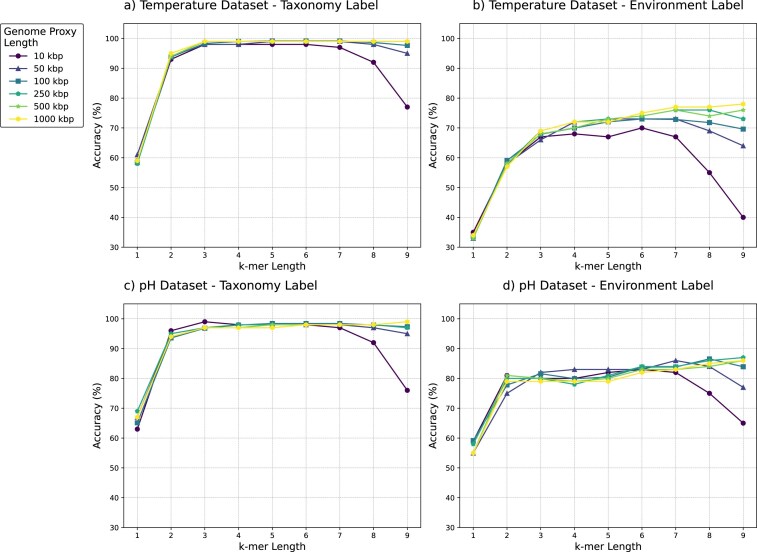
Classification accuracy of SVM classifier under bias mitigation scenario. (**A**) ‘Temperature dataset’ with taxonomy labels. (**B**) ‘Temperature dataset’ with environment-type labels. (**C**) ‘pH dataset’ with taxonomy labels. (**D**) ‘pH dataset’ with environment-type labels. Each subfigure shows accuracy across nine $k$-mer sizes and six genome proxy lengths.

Figure 3 presents the classification accuracy results of SVM classifiers applied to both the ‘Temperature dataset’ and the ‘pH dataset’ under the bias mitigation scenario, with taxonomy and environment-type labeling, respectively. This figure illustrates how the classification accuracy changes as the value of $k$ increases, for the six different genome proxy lengths analyzed. The classification accuracies for the other five classifiers, and for all six classifiers under the standard scenario, for both the ‘Temperature dataset’ and the ‘pH dataset’ are similar, and can be found in the Supplementary Materials, Section D.

As seen in Fig. 3, increasing the length of $k$-mer from 1 to 6 leads to a significant improvement in classification accuracy. For values of $k$ higher than 6, the changes in accuracy depend on the genome proxy length. For longer genome proxies (100, 250, 500, and 1000 kbp), the taxonomic classification accuracy remains stable for increasing values of $k$ from $k=6$ to $k=9$, and the environment-type classification accuracy increases with the increase in $k$-mer size. However, for shorter genome proxies (10 and 50 kbp), both the taxonomic and environment-type accuracies decrease with the increase of $k$-mer sizes from 6 to 9.

The decline of classification accuracy with the $k$-mer size increase, when $k$ is higher than a certain threshold, is due to the fact that the increase in the length of the $k$-mer frequency feature vector is exponential in $k$. For small values of $k$, this increase results in more information available to the classifier. However, the number of $k$-mers that actually occur in the sequence is bound by the length of the sequence. Thus, after $k$ passes a certain threshold, the feature vector becomes so sparse that it increasingly fails to capture the genomic patterns necessary for an accurate classification. This threshold is reached earlier for shorter sequences (10 or 50 kbp) than for longer sequences.

Since, for all genome proxy lengths considered, the classification accuracies increase until $k=6$, we concluded that the value of $k$ should be 6 at the minimum, and performed a detailed analysis for values $k=6, 7, 8, 9$.

The detailed analysis for $k$-mer sizes of 6–9 for the ‘Temperature dataset’ shows that the highest taxonomic classification accuracy for the six fragment lengths considered in the bias mitigation scenario ranges from 98.15% to 99.50%, and the highest environment-type classification accuracy ranges from 70.29% to 78.14%. Also, the results for the ‘pH dataset’ indicate that the highest taxonomic classification accuracy for different fragment lengths ranges from 97.89% to 98.95%, and for environment-type classification ranges from 83.30% to 87.10%. The classifier’s performance in the standard scenario is similar.

The detailed results of these experiments for both bias mitigation and standard scenarios can be found in Supplementary Materials, Section E. Overall, one observes that increasing the value of $k$ from 6 to 9 does not result in significant increases in classification accuracy. This, combined with the fact that increasing $k$ leads to an exponential increase in memory usage (the feature vector size increases from $2^{12}$ to $2^{18}$) and time complexity, leads to the conclusion that $k=6$ is the optimal choice for the $k$-mer size in this context.

In the next step, we maintained a fixed $k$-mer size of $k=6$ and assessed the effectiveness of six classifiers for the six genome proxy lengths considered in this study. This allowed us to identify the optimal genome proxy length for both the ‘Temperature dataset’ and ‘pH dataset’. Table 5 displays the highest classification accuracy achieved for each genome proxy length, for both the standard scenario and the bias mitigation scenario. As observed in Table 5, a fragment length of 100 kbp achieves the highest accuracy in three of the classification tasks: the standard taxonomic classification for both datasets and the bias mitigation taxonomic classification for the ‘Temperature dataset’. In the remaining cases, the difference between the best performance and the 100 kbp performance was <0.5% in the standard scenario and <1% in the bias mitigation scenario. Thus, a genome proxy length of 100 kbp (at $k=6$) is the optimal overall selection.

**Table 5.** Comparison of the best classification accuracy across all classifiers, using $k=6$, the optimal chosen value for $k$, for all six genome proxy lengths

Dataset	Genome proxy length	Label type	Standard scenario	Bias mitigation scenario
			accuracy (%)	accuracy (%)
Temperature	10 kbp	Taxonomy	98.50	98.20
		Environment	82.00	70.30
	50 kbp	Taxonomy	**99.50**	99.00
		Environment	83.80	72.80
	100 kbp	Taxonomy	**99.50**	**99.20**
		Environment	85.10	74.80
	250 kbp	Taxonomy	**99.50**	**99.20**
		Environment	84.80	74.30
	500 kbp	Taxonomy	**99.50**	**99.20**
		Environment	84.80	74.40
	1000 kbp	Taxonomy	**99.50**	**99.20**
		Environment	**85.30**	**75.10**
pH	10 kbp	Taxonomy	97.80	97.90
		Environment	89.20	83.30
	50 kbp	Taxonomy	**98.40**	98.40
		Environment	91.30	84.90
	100 kbp	Taxonomy	**98.40**	98.40
		Environment	93.10	85.50
	250 kbp	Taxonomy	**98.40**	**98.90**
		Environment	93.00	86.00
	500 kbp	Taxonomy	**98.40**	**98.90**
		Environment	92.00	**86.00**
	1000 kbp	Taxonomy	**98.40**	97.90
		Environment	**93.50**	**86.00**

All occurrences of maximum accuracy are shown in bold, and the performance for a fragment length of 100 kbp is shown as underlined.

In our last experiment, our objective was to determine whether using a short genome proxy might lead to any loss of information compared to using the whole genome. To evaluate this, we performed taxonomic and environment-type classification using entire genomes, while maintaining the same setup as our previous supervised experiment, under bias mitigation scenarios with = 6. Our findings show that for taxonomic classification of whole genomes with $k=6$, the accuracy was 99.15% (compared to 99.20% using random 100 kbp genome proxies) for the ‘Temperature dataset’, and 98.42% (compared to 98.40%) for the ‘pH dataset’. For environment-type classification, the best accuracy for whole genomes was 75.51% (compared to 73.00%) for the ‘Temperature dataset’, and 84.35% (compared to 85.50%) for the ‘pH dataset’. These results indicate that classification accuracy using a genome proxy of length 100 kbp is comparable to using the entire genome, which in our datasets has an average length of 3500 kbp (the genome proxy is 35 times shorter on average).

### A multilayered pipeline to find bacterium–archaeon pairs with similar genomic signatures

The identification of bacterium–archaeon pairs is a multilayered filtering process that progressively narrows down the candidate pairs generated through unsupervised clustering, to reach the environment-related bacterium–archaeon pairs.

**Figure 4.**
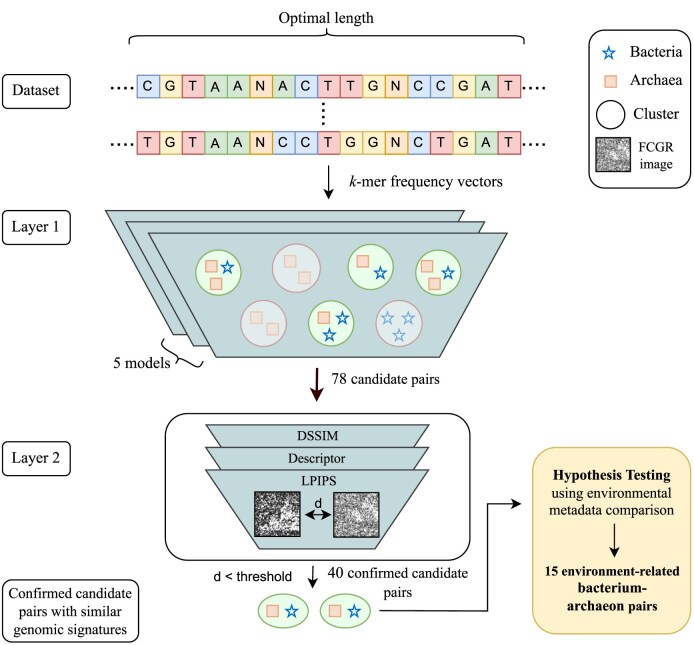
Multilayered pipeline for identifying bacterium–archaeon pairs with similar genomic signatures. *Layer 1:* Five selected nonparametric clustering methods identify clusters of organisms with similar genomic signatures. The clusters containing both bacteria and archaea (green) generate a list of 78 candidate bacterium–archaeon pairs, grouped by these algorithms based on their similar genomic signatures. *Layer 2:* The candidate pairs from Layer 1 undergo pairwise distance calculations between their FCGRs using four different distance metrics. Only 40 pairs, with the majority of distances below empirically determined thresholds, are retained. *Hypothesis Testing:* after identifying confirmed candidate bacterium–archaeon pairs with similar genomic signatures, a biological analysis is conducted. This includes checking environment labels and examining metadata about their living environments to select pairs isolated from similar types of extreme environments. The final output is a list of 15 environment-related bacterium–archaeon pairs (comprising 16 unique genera and 20 unique species) that have similar genomic signatures and passed the hypothesis testing. These pairs can confidently be proposed as maximally taxonomically divergent microbes (from different domains, Bacteria and Archaea) that share similar genomic signatures associated with their living environments.

Figure 4 illustrates the details of this multilayered filtering approach. We further investigated the 3-mer usage bias in these 15 environment-related bacterium–archaeon pairs (which passed all filtering layers) and found that they demonstrate a similar genomic signature linked to their extreme environment despite their maximal taxonomic differences. As the last analysis, we also studied the co-occurrence of environment-related pairs.

#### Layer 1: nonparametric clustering

We initiated the process using nonparametric clustering algorithms in combination with dimensionality reduction methods. As described in the ‘Materials and methods’ section, we evaluated the contamination and completeness scores of the clusters and identified the top five performing clustering methods, selecting those that performed best at generating clusters that correspond to true genera.

From the clusters obtained using the chosen algorithms, a set of candidate pairs, consisting of bacterium–archaeon pairs whose genomic signatures were consistently clustered together by the majority of the algorithms, was identified for each dataset. To ensure robustness, we repeated the above analysis (clustering and selecting bacterium–archaeon pairs) 10 times. We then selected the bacterium–archaeon pairs that appeared in at least 5 of the 10 runs. This initial step generated 78 candidate bacterium–archaeon pairs (38 unique genera, 85 unique species).

#### Layer 2: FCGR comparison of candidate pairs

In the second layer, we filtered the candidate pairs based on their FCGR distances. As described in the ‘Materials and methods’ section, we calculated the FCGR images for each pair of sequences, using a genome proxy length of 100 kbp and a $k$ value of 6, and measured the distances between these FCGRs using three distance metrics. We selected bacterium–archaeon pairs with distances below empirically determined thresholds for the majority of distance metrics.

**Figure 5.**
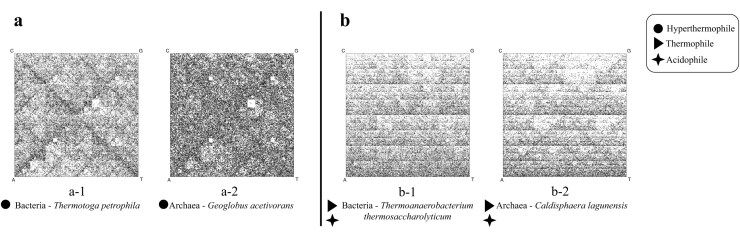
FCGR images of two confirmed candidate pairs (four unique species), with a resolution of $k$ = 8. The first pair includes (**a-1**) a hyperthermophilic bacterium and (**a-2**) a hyperthermophilic archaeon, while the second pair consists of (**b-1**) an acidophilic thermophilic bacterium and (**b-2**) an acidophilic thermophilic archaeon. The first pair was drawn from the ‘Temperature dataset’, and the second pair appears in both ‘Temperature dataset’ and ‘pH dataset’. In both candidate pairs, the FCGRs display strikingly similar patterns between the two species, despite belonging to different taxonomic domains (Bacteria and Archaea).

After this filtering layer, we identified 40 confirmed candidate pairs (32 unique genera, 48 unique species), with similar FCGR images, determined by the three distance metrics. The members of each of these confirmed pairs can now be confidently considered as having similar genomic signatures (see Supplementary Materials, Section F for details). Figure 5 shows the FCGR images of two pairs, one extremophile (*Thermotoga petrophila* and *Geoglobus acetivorans*) and one polyextremophile pair (*Thermoanaerobacterium thermosaccharolyticum* and *Caldisphaera lagunensis*). For better visualization, the value $k$ = 8 was used, and the images confirmed that the FCGRs show visual pattern similarities, in addition to the distance between FCGRs being below the empirically determined threshold.

#### Hypothesis testing using isolating environment metadata of confirmed candidate pairs

To test the hypothesis that the genomic signature similarities between the confirmed candidate pairs result from shared environmental pressures, we conducted a comparison of environmental metadata of their isolation habitats. Out of 40 confirmed candidate pairs obtained from the multilayered pipeline, 18 pairs initially passed hypothesis testing. However, three bacterium–archaeon pairs were excluded because the archaeon’s reference genome was recently suppressed on NCBI (see Supplementary Materials, Section F). This left a final set of 15 confirmed candidate pairs, representing 16 unique genera and 20 unique species. These pairs, validated by their isolation environment metadata and labels, are proposed as environment-related bacterium–archaeon associations. The details of the environmental data of the selected pairs can be found in the Supplementary Materials, Section G.

**Figure 6.**
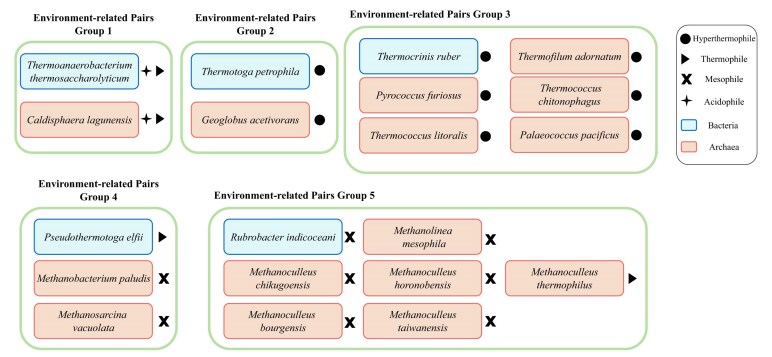
Environment-related pairs, grouped by the bacterial species. Each bacterium–archaeon pair belonged to the same cluster generated by the clustering algorithms, and passed both the FCGR comparison and the hypothesis testing layers. The environment-related pairs set comprises 20 species, including five bacteria and 15 archaea from 16 unique genera. Among these, two species are poly-extremophiles (acidophilic thermophiles), 10 are extremophiles (eight hyperthermophiles and two thermophiles), and the remaining eight are mesophiles.

Since these pairs revealed cases where multiple archaea were grouped with a single bacterium, we organized these pairs into 5 groups based on the bacterial species. Notably, Groups 1, 2, and 3 include sequences of organisms isolated from extreme environments, while the majority of organisms in Groups 4 and 5 are associated with normal temperature (mesophiles) and normal pH (absent from the ‘pH dataset’) conditions. We further examined the 3-mer usage bias of species in these confirmed 15 pairs, as well as their co-occurrences. For Groups 4 and 5, we also investigated any potential extreme conditions in their environments other than extreme temperature or pH. The details of these five groups are shown in Fig. 6 , and their FCGR images can be found in Supplementary Materials, Section H.

#### Analysis of 3-mer frequency profiles of environment-related bacterium–archaeon pairs

To investigate potential biases in 3-mer usage associated with environmental adaptation, we conducted a detailed analysis of the 3-mer frequency profiles for the genome proxies of the organisms in the environment-related bacterium–archaeon pairs groups. We focused on $k=3$ due to its biological relevance, since the set of codons is a subset of the set of 3-mers. Following the four-step analysis, this section examines how 3-mer frequencies reflect environmental adaptations across taxonomically divergent microbes. The results of this analysis are summarized in Table 6 for each of the five environment-related bacterium–archaeon pairs groups.

**Table 6.** Combined summary of 3-mer profile analysis for Groups 1–5

			Shared			Overall	Biology literature
			environment			3-mer	observed
			relevant			similarity	shared
Group	Bacterium	Archaeon	3-mers (ratio)	*rho*	Score		3-mers
Group 1	*T. thermosaccharolyticum*	*C. lagunensis*	0.83	0.96	0.89	Compelling	9
Group 2	*T. petrophila*	*G. acetivorans*	1.00	0.81	0.90	Compelling	4
Group 3	*Thermocrinis ruber*	*Thermofilum adornatum*	1.00	0.77	0.89	Compelling	5
		*Thermococcus chitonophagus*	1.00	0.81	0.90	Compelling	4
		*Palaeococcus pacificus*	0.90	0.76	0.83	Very strong	4
		*Pyrococcus furiosus*	0.89	0.81	0.85	Compelling	5
		*Thermococcus litoralis*	0.91	0.80	0.85	Compelling	4
Group 4	*Pseudothermotoga elfii*	*Methanobacterium paludis*	1.00	0.94	0.97	Compelling	1
		*Methanosarcina vacuolata*	0.75	0.95	0.85	Compelling	1
Group 5	*Rubrobacter indicoceani*	*Methanolinea mesophila*	0.60	0.83	0.71	Moderate	1
		*Methanoculleus chikugoensis*	0.77	0.93	0.8	Compelling	3
		*Methanoculleus bourgensis*	0.73	0.91	0.82	Very strong	2
		*Methanoculleus horonobensis*	0.69	0.95	0.82	Very strong	3
		*Methanoculleus taiwanensis*	0.64	0.92	0.78	Strong	3
		*Methanoculleus thermophilus*	0.78	0.89	0.83	Very strong	0

For each pair, we calculated the number of shared environment-relevant 3-mers exhibiting similar over- or under-representation patterns between the two species of the pair, and reported the ratio out of 15. The Spearman rank correlation coefficient ($rho$) was computed to quantify the correlation between the 3-mer representation patterns of each pair; all correlations were statistically significant (*P* < 10^−5^). A combined score was calculated as the average of the shared environment-relevant 3-mer ratio and $rho$ to assess the overall similarity of each pair. For further validation, the last column reports the number of shared environment-relevant 3-mers that have over- or under-representation patterns consistent with findings in the biological literature.

For each environment-related pair, the set of ‘shared environment-relevant 3-mers’ is defined as the intersection of the set of environment-relevant 3-mers of the bacterium genome proxy with that of the archaeon genome proxy. Among these shared 3-mers, we calculated the proportion of 3-mers that show the same pattern of over- or under-representation in both species and reported it in Table 6. Additionally, we calculated the Spearman rank correlation coefficient ($rho$) between the 3-mer representation patterns of the two organisms in each pair. Notably, all correlations were statistically significant with *P* < 10^−5^ for all pairs. Since the shared 3-mer ratio and $rho$ collectively represent the results of steps 1–3 of the 3-mer frequency profile analysis pipeline (see the ‘Materials and methods’ section), we calculated a combined score as the average of these two values to provide an overall measure of 3-mer frequency profile similarity between the species of each pair. Based on the combined score, we also assigned a descriptive term to each pair for a clear comparison. Specifically, we labeled pairs as ‘Compelling’ for scores ≥0.85, ‘Very strong’ for scores between 0.85 and 0.80, ‘Strong’ for scores between 0.80 and 0.75, and ‘Moderate’ for scores between 0.75 and 0.70. These thresholds were determined empirically based on the distribution of our results.

Finally, as outlined in the last step of the 3-mer frequency profile analysis, we examined the biological literature on codon usage to determine whether the observed over- or under-representation of each shared environment-relevant 3-mer had been previously reported in biological literature. The final column of Table 6 reports the number of shared 3-mers for which our findings in over- or under-representation align with evidence from prior studies, providing further validation of the observed similarities. Importantly, we did not include this literature-based validation in the combined score calculation, as low values in this step may only reflect a lack of prior research in the literature rather than a true biological absence.

The results revealed nine pairs with compelling 3-mer similarity, four pairs with very strong similarity, one pair with strong similarity and one pair with moderate similarity. No pairs exhibited very low similarity (the minimum similarity score is 0.71), indicating a moderate to high level of 3-mer frequency profile similarity across all confirmed pairs. Notably, the first three groups, which include poly-extremophile or extremophiles, showed a higher average number of shared environment-relevant 3-mers observed in the biological literature (average: five) compared to Groups 4 and 5, which predominantly consist of mesophiles (average: two).

Interestingly, despite being composed mainly of mesophiles, Groups 4 and 5 included two pairs with compelling similarity and four pairs with very strong similarity. This unexpected finding suggests that factors beyond temperature or pH, such as other environmental pressures, may contribute to genomic sequence composition convergence in these pairs, which is further discussed in the ‘Discussion’ section. Detailed results of the 3-mer frequency profile analysis are presented in Supplementary Materials, Section I.

#### Co-occurrence of organisms from the confirmed bacterium–archaeon pairs

In the final analysis, using the MAP tool [65], we analyzed the habitats of all environment-related pairs within their respective groups, to identify any shared environments.

This analysis revealed distinct patterns of co-occurrence across different groups. Both species in **Group 1** were found together in Washburn Hot Springs, a geothermal hot spring in Yellowstone National Park, Wyoming, USA [67]. Notably, this co-occurrence habitat differs from the environments where the species were originally isolated [68–70]. Despite the large geographic distances between the original isolation and co-occurrence sites, these habitats have similar environmental pressures and geochemical properties.

Similar observations were made for the species in the pair of **Group 2**, which were found to co-occur in two distinct habitats: Brothers Volcano, a submarine volcano in the Pacific Ocean near New Zealand [71], and Juan de Fuca Ridge, a mid-ocean ridge flank near Vancouver Island [72]. Note that these species were initially isolated from a deep Japanese oil reservoir [73] and a deep-sea hydrothermal vent [74], respectively.

In **Group 3**, a subset of species co-occurred in environments overlapping with those of Group 1 and Group 2, including Brothers Volcano and Washburn Hot Springs. Additional co-occurrence sites were found across Yellowstone National Park. Similar to Group 1 and Group 2, the environmental conditions of the co-occurrence habitats resemble the conditions of isolating environments of the respective species. It is worth mentioning that even though species from Groups 1, 2, and 3 were found to co-occur in the same habitat, our clustering methods provide the sensitivity to detect specific 3-mer biases within their genomic signatures. This enables classification based on their evolved niche adaptations rather than their current habitat, which explains why these groups were clustered separately despite sometimes sharing the same environment. Detailed geographic maps and co-occurrence data for these three groups can be found in the Supplementary Materials, Section J.

Although the majority of species in **Group 4** are mesophiles, they co-occurred in multiple independent environments characterized by other common extreme environment conditions, such as anaerobic and methanogenic conditions. These habitats include the Shengli Oil Field in China, hypothesized to involve anaerobic, mesophilic microbiomes in the ‘methanogenic degradation of hydrocarbons’ [75], and a Japanese bioreactor [76]. No co-occurrence was identified for **Group 5** species. Detailed geographic maps and co-occurrence for Group 4 and Group 5 can be found in Supplementary Materials, Section J.

Importantly, this co-occurrence analysis supports the bacterium–archaeon pairs clusters identified by our multilayered approach. Indeed, it demonstrates that many of the species pairs that were computationally grouped together by our method, despite being originally isolated from different environments, were later found to co-occur naturally in shared environments distinct from their isolation sites.

## Discussion

Our computational analysis revealed that both taxonomic and environmental components can be pervasive throughout extremophile prokaryotic genomes, suggesting that environmental adaptations influence the entire genome rather than specific genic or regulatory regions exclusively. Indeed, our novel computational pipeline resulted in high classification and clustering accuracies, despite using as ‘genome proxy’ a relatively short DNA fragment constructed by the pseudo-concatenation of 10 randomly selected 10 000 bp fragments (total length 100 000 bp, that is $\approx 35$ times shorter than a complete genome). This indicates that taxonomic and environmental components are detectable even with limited genomic samples, which has important implications for studying environmental adaptations when complete genome sequences are not available.

Our multilayered approach identified 15 pairs of maximally distant organisms that have similar genomic signatures, grouped into five distinct categories. The statistically significant 3-mer over-representation and under-representation analysis further confirmed the genomic composition similarity of these pairs. Notably, the identified environmentally-relevant 3-mer representation patterns align with known extremophile adaptation mechanisms as detailed below.

In **Group 1**, the over-representation of the 3-mer ‘CAA’ (corresponding to a glutamine codon) in the genome of thermophilic acidophiles aligns with previous findings of codon usage bias in acidophilic prokaryotes which prefer the ‘CAA’ codon when calling for glutamine [18]. Moreover, the under-representation of the 3-mer ‘ACG’ (corresponding to a threonine codon) in this group is consistent with amino acid abundance patterns found in thermophilic prokaryotic proteins which demonstrate a relative lack of threonine [77].

In **Group 2** and **Group 3**, consisting of hyperthermophiles, observations of the elevated representation of 3-mers corresponding to arginine codons, and decreased representation of 3-mers corresponding to asparagine and glutamine codons align with previous observations related to amino acid abundances in hyperthermophilic and thermophilic prokaryotic proteins [17, 78, 79]. Specifically, (hyper)thermophilic proteins demonstrate an increased abundance of arginine, and decreased abundance of asparagine and glutamine amino acids, which is reflected by the relative representations of 3-mers respectively. Note that several species in Group 3, specifically, *T. ruber* (bacteria) and three archaeal species (*P. furiosus, T. litoralis*, and *Pyrococcus chitonophagus*), were previously identified as having similar genomic signatures by using slightly different methods [25], which further validates our multilayered approach.

The 3-mer frequency profile analysis of **Group 4** also showed some agreement with known codon usage patterns. In this group, all species, including the thermophilic bacterium, exhibited an under-representation of 3-mers corresponding to a serine codon. This pattern aligns for mesophilic species, which demonstrate a codon usage bias against the 3-mer ‘AGC’ when calling for serine [80] and with the observed lower serine amino acid in thermophilic proteins relative to mesophilic proteins [81]. The grouping of mesophilic species from maximally divergent taxa in Group 4, along with their similarity in genomic compositions and 3-mer representations, suggests the influence of extreme environmental pressures beyond temperature and pH. Indeed, we observed that Group 4 species co-occur in anaerobic, methanogenic environments and share the phenotypic trait of oxygen intolerance (Supplementary Materials, Section F). This indicates that additional extreme factors, such as high concentrations of endogenously-produced methane, or exogenous hydrocarbons encountered in oil fields or wells, could potentially influence extremophilic genomic signature composition.

In **Group 5**, in contrast with Groups 1, 2, 3, and 4, our findings revealed unique genomic signature patterns that differ from previous biological findings of extremophile codon usage bias. In this group, our findings showed an under-representation of the 3-mer ‘CTA’, which codes for leucine. This was expected in mesophilic species of this group, as mesophilic prokaryotes commonly exhibit a bias against using this codon [80]. However, surprisingly, we found the same under-representation in the thermophilic species of this group, in contrast with previous studies which showed ‘CTA’ to be typically abundant in other thermophiles [17]. Our finding contradicts previous assumptions of codon usage bias in thermophilic prokaryotes, suggesting that the impact of environmental adaptation on prokaryotic genomes may be more nuanced than previously thought and needs further investigation. Although no co-occurrence environments were found for Group 5, their initial isolation from predominantly methanogenic habitats, as described in their discovering papers, suggests a potential role of methanogenic processes in shaping the selection of, and thus the composition of genomic signatures of these species [82]. Further investigation is needed to clarify these relationships.

It is worth noting that horizontal gene transfer, a phenomenon in which one species can transfer genetic material to another, is a major driver of adaptation in extreme environments [83]. However, a Basic Local Alignment Search Tool (BLAST) [84] analysis showed little to no evidence of extensive or localized transfer between species across the five Groups in our study (see Supplementary Materials, Section K). Only the discontinuous megablast parameters revealed a 1%–2% query cover between archaeal genomes and the bacterium in each Group. This indicates that the archaeal genomes share minimal genetic material with the bacterial genome in the same group, as expected. Moreover, within these aligned regions, the genetic sequences show only moderate similarity, which suggests that the genetic material is not highly conserved. This finding contrasts with what we typically see in extreme environments, where genes that provide survival advantages are usually highly conserved [85]. Thus, while alignment-based approaches confirm the local absence of shared genetic material between archaeon and bacterium pair members, our techniques reveal the presence of shared genomic composition patterns throughout their entire genomes.

Our computational approach also has some limitations. For example, this $k$-mer-based method cannot capture long-range genomic interactions, although this could potentially be addressed through the use of transformer models [86]. Additionally, the exponential growth in the size of feature vectors with increasing $k$-mer size limited our analysis to $k \le 9$, potentially obscuring larger sequence patterns. Also, while the parameters that were empirically determined to be optimal proved effective for the classification/clustering of this extremophile dataset, they may not generalize across all genomic analyses, as they likely depend on dataset characteristics and the complexity of the classification task. Lastly, the choice of the distance thresholds can depend on the datasets, and this choice is discussed in Supplemental Materials, Section L.

A point that warrants further discussion is the choice of the parameter $n$, which determines how many randomly selected DNA sub-fragments are pseudo-concatenated into a single composite genome proxy for computational analysis. In this study, all experiments were conducted with $n=10$. This being said, as detailed in Section M of the Supplementary Materials, a comprehensive analysis shows that larger values of $n$, up to $n=10{,}000$ for a genome proxy length of 100 000, still capture the global environmental and taxonomic components, even though the resulting sub-fragments are as short as 10 bp. Remarkably, these settings achieved classification accuracies of 99.16% for taxonomic classification and 73.16% for environment-type classification in the ‘Temperature dataset’, and 98.32% and 83.27% for the ‘pH dataset’, respectively. These results demonstrate that a 100 000 bp genome proxy constructed from sub-fragments as short as 10 bp can still capture taxonomic and environmental patterns. We also extended this analysis to other genome proxy lengths (ranging from 10 000 bp to 1000 kbp) and obtained consistent high classification performance (over 97% for taxonomic and over 70% for environment-type classification) with sub-fragments as short as 10 bp.

Overall, our findings demonstrate that extreme environmental adaptation significantly impacts prokaryotic genomic signature compositions, with environmental pressures capable of overriding traditionally recognized taxonomic influences. The biological significance of our approach is highlighted by the discovery of 15 microbial species pairs that share genomic signatures despite maximal taxonomic divergence, suggesting that shared environmental pressures can drive convergent genome sequence composition across vastly different species. These results provide compelling evidence that environment-driven genomic components persist across diverse taxa, offering new perspectives on how environment-associated mutagenesis and selection shape microbial genomes. Our work broadens the field’s perspective beyond the traditional focus on phenotype, proteome, and gene-specific analyses to genome-wide considerations. By bridging computational methods with biological context, this work advances machine learning applications in genomics and our understanding of extremophile adaptation mechanisms. Future research will explore the biological mechanisms underlying these shared genomic signatures and their implications for evolutionary biology, biotechnology, and environmental genomics.

## Supplementary Material

lqaf189_Supplemental_File

## Data Availability

All sequence data used in this paper, unique assembly accession IDs of all the sequences, and the metadata are available at https://doi.org/10.5281/zenodo.17148766.

## Code availability

All code used in this study is publicly available on GitHub at https://github.com/Kari-Genomics-Lab/Extreme_Env_2 and archived in the Zenodo repository at https://doi.org/10.5281/zenodo.17172798.
