# Supplementary material for: Life at the extremes: maximally divergent microbes with similar genomic signatures linked to extreme environments
Source: NAR Genom Bioinform. 2025 Dec 23;7(4):lqaf189. doi: 10.1093/nargab/lqaf189 (PMC12723239; doi:10.1093/nargab/lqaf189)
Supplement: lqaf189_Supplemental_File [file lqaf189_supplemental_file.pdf]

Supplementary Materials for the paper "Life at the extremes: Maximally divergent microbes with similar genomic signatures linked to extreme environments"

Monireh Safari, Joseph Butler,
Gurjit S. Randhawa, Kathleen A. Hill, Lila Kari

A Dataset Details

In this study, we used two datasets of microbial genomes that live in extreme temperature (*Temperature Dataset*) or extreme pH (*pH Dataset*). In total, there are 91 species that are shared in both the *Temperature dataset* and *pH dataset*. Eight genomes are mesophiles and live in acidic or alkaline environments, as detailed in Table S1. From the remaining 83 genomes that are poly-extremophiles, there are 40 Archaea (Table S2), and 43 Bacteria (Table S3).

Table S1: Genomes that are listed in both the *Temperature* and *pH datasets*. The domain of all the sequences in this category is Archaea. These genomes are not poly-extremophiles, as their temperature label is mesophile.

**Assembly ID**	**Species**	**Temperature Label**	**pH Label**
GCA_000336935.1	*Halococcus salifodinae*	Mesophiles	Alkaliphiles
GCA_000337135.1	*Natrialba chahannaoensis*	Mesophiles	Alkaliphiles
GCA_000337575.1	*Natrialba hulunbeirensis*	Mesophiles	Alkaliphiles
GCA_001971705.1	*Natronorubrum daqingense*	Mesophiles	Alkaliphiles
GCA_004745425.1	*Methanolobus sp004745425*	Mesophiles	Alkaliphiles
GCA_014647115.1	*Halarchaeum rubridurum*	Mesophiles	Acidophiles
GCA_900104065.1	*Natronobacterium texcoconense*	Mesophiles	Alkaliphiles
GCA_900188065.1	*Halorubrum vacuolatum*	Mesophiles	Alkaliphiles

Table S2: The poly-extremophiles from Archaea domain, shared between both the *Temperature Dataset* and *pH Dataset*.

**Assembly ID**	**Species**	**Temperature Label**	**pH Label**
GCA_000008665.1	*Archaeoglobus fulgidus*	Hyperthermophiles	Alkaliphiles
GCA_000018305.1	*Caldivirga maquilingensis*	Hyperthermophiles	Acidophiles
GCA_000018365.1	*Thermococcus onnurineus*	Hyperthermophiles	Alkaliphiles
GCA_000022485.1	*Saccharolobus islandicus*	Hyperthermophiles	Acidophiles
GCA_000148385.1	*Vulcanisaeta distributa*	Hyperthermophiles	Acidophiles
GCA_000215995.1	*Pyrococcus yayanosii*	Hyperthermophiles	Acidophiles
GCA_000223395.1	*Pyrolobus fumarii*	Hyperthermophiles	Acidophiles
GCA_000253055.1	*Thermoproteus tenax*	Hyperthermophiles	Acidophiles
GCA_001748385.1	*Vulcanisaeta_B thermophila*	Hyperthermophiles	Acidophiles
GCA_002116695.1	*Acidianus manzaensis*	Hyperthermophiles	Acidophiles
GCA_009729015.1	*Acidianus ambivalens*	Hyperthermophiles	Acidophiles
GCA_014646555.1	*Vulcanisaeta souniana*	Hyperthermophiles	Acidophiles
GCA_900079115.1	*Saccharolobus solfataricus*	Hyperthermophiles	Acidophiles
GCA_000011185.1	*Thermoplasma volcanium*	Thermophiles	Acidophiles
GCA_000012285.1	*Sulfolobus acidocaldarius*	Thermophiles	Acidophiles
GCA_000025665.1	*Aciduliprofundum boonei*	Thermophiles	Acidophiles
GCA_000144915.1	*Acidilobus saccharovorans*	Thermophiles	Acidophiles
GCA_000193375.1	*Thermoproteus uzoniensis*	Thermophiles	Acidophiles
GCA_000195915.1	*Thermoplasma acidophilum*	Thermophiles	Acidophiles
GCA_000196895.1	*Halalkalicoccus jeotgali*	Thermophiles	Alkaliphiles
GCA_000204925.1	*Metallosphaera cuprina*	Thermophiles	Acidophiles
GCA_000243315.1	*Metallosphaera yellowstonensis*	Thermophiles	Acidophiles
GCA_000317795.1	*Caldisphaera lagunensis*	Thermophiles	Acidophiles
GCA_000336615.1	*Haloarcula amylolytica*	Thermophiles	Alkaliphiles
GCA_000336755.1	*Haloferax elongans*	Thermophiles	Acidophiles
GCA_000337735.1	*Natronorubrum sulfidifaciens*	Thermophiles	Alkaliphiles
GCA_000455345.1	*Halopiger_A goleimassiliensis*	Thermophiles	Alkaliphiles
GCA_000508305.1	*Sulfolobus acidocaldarius_A*	Thermophiles	Acidophiles
GCA_000632495.1	*Acidianus copahuensis*	Thermophiles	Acidophiles
GCA_001719125.1	*Saccharolobus sp001719125*	Thermophiles	Acidophiles
GCA_002153915.1	*Methanonatronarchaeum thermophilum*	Thermophiles	Alkaliphiles
GCA_003201675.2	*Metallosphaera hakonensis*	Thermophiles	Acidophiles
GCA_003201765.2	*Acidianus sulfidivorans*	Thermophiles	Acidophiles
GCA_003201835.2	*Acidianus_B brierleyi*	Thermophiles	Acidophiles
GCA_003967175.1	*Sulfodiicoccus acidiphilus*	Thermophiles	Acidophiles
GCA_009729035.1	*Stygiolobus azoricus*	Thermophiles	Acidophiles
GCA_009729055.1	*Sulfurisphaera ohwakuensis*	Thermophiles	Acidophiles
GCA_013340765.1	*Conexivisphaera calida*	Thermophiles	Acidophiles
GCA_013343295.1	*Metallosphaera tengchongensis*	Thermophiles	Acidophiles
GCA_900176435.1	*Picrophilus oshimae*	Thermophiles	Acidophiles

Table S3: The poly-extremophiles from Bacteria domain, shared between both the *Temperature Dataset* and *pH Dataset*.

Assembly ID	Species	Temperature Label	pH Label
GCA_000014185.1	*Rubrobacter_B xylanophilus*	Thermophiles	Alkaliphiles
GCA_000015025.1	*Acidothermus cellulolyticus*	Thermophiles	Acidophiles
GCA_000024285.1	*Alicyclobacillus acidocaldarius*	Thermophiles	Acidophiles
GCA_000092425.1	*Truepera radiovictrix*	Thermophiles	Alkaliphiles
GCA_000145615.1	*Thermoanaerobacterium thermosaccharolyticum*	Thermophiles	Acidophiles
GCA_000175575.2	*Acidithiobacillus_A caldus*	Thermophiles	Acidophiles
GCA_000219875.1	*Alicyclobacillus acidocaldarius_A*	Thermophiles	Acidophiles
GCA_000226295.1	*Chloracidobacterium thermophilum*	Thermophiles	Alkaliphiles
GCA_000421625.1	*Thermus islandicus*	Thermophiles	Acidophiles
GCA_000429525.1	*Alicyclobacillus_A contaminans*	Thermophiles	Acidophiles
GCA_000430585.1	*Alicyclobacillus_G herbarius*	Thermophiles	Acidophiles
GCA_000444055.1	*Alicyclobacillus acidoterrestris*	Thermophiles	Acidophiles
GCA_000472905.1	*Alicyclobacillus_A pomorum*	Thermophiles	Acidophiles
GCA_000833605.1	*Anoxybacillus ayderensis*	Thermophiles	Alkaliphiles
GCA_000953475.1	*Methylacidiphilum fumariolicum*	Thermophiles	Acidophiles
GCA_001280565.1	*Sulfobacillus thermosulfidooxidans_A*	Thermophiles	Acidophiles
GCA_001447355.1	*Alicyclobacillus tengchongensis*	Thermophiles	Acidophiles
GCA_001552255.1	*Alicyclobacillus_G shizuokensis*	Thermophiles	Acidophiles
GCA_001552655.1	*Alicyclobacillus_G kakegawensis*	Thermophiles	Acidophiles
GCA_001552675.1	*Alicyclobacillus sendaiensis*	Thermophiles	Acidophiles
GCA_001570745.1	*Alicyclobacillus mali*	Thermophiles	Acidophiles
GCA_003057965.1	*Thermodesulfobium acidiphilum*	Thermophiles	Acidophiles
GCA_004366795.1	*Alicyclobacillus sacchari*	Thermophiles	Acidophiles
GCA_007475525.1	*Methylacidiphilum kamchatkense*	Thermophiles	Acidophiles
GCA_007991715.1	*Alicyclobacillus acidoterrestris_A*	Thermophiles	Acidophiles
GCA_013760845.1	*Anoxybacillus_B calidus*	Thermophiles	Alkaliphiles
GCA_014196195.1	*Anoxybacillus_A rupiensis*	Thermophiles	Alkaliphiles
GCA_014201585.1	*Anoxybacillus tengchongensis*	Thermophiles	Alkaliphiles
GCA_017298635.1	*Alicyclobacillus_B ferrooxydans_B*	Thermophiles	Acidophiles
GCA_017310505.1	*Methylacidiphilum sp004421255*	Thermophiles	Acidophiles
GCA_900107035.1	*Alicyclobacillus hesperidum*	Thermophiles	Acidophiles
GCA_900111795.1	*Anoxybacillus pushchinoensis*	Thermophiles	Alkaliphiles
GCA_900116805.1	*Alicyclobacillus_H macrosporangiidus*	Thermophiles	Acidophiles
GCA_900129115.1	*Thermoanaerobacter uzonensis*	Thermophiles	Acidophiles
GCA_900142255.1	*Alicyclobacillus_I montanus*	Thermophiles	Acidophiles
GCA_900156755.1	*Alicyclobacillus vulcanalis*	Thermophiles	Acidophiles
GCA_900176145.1	*Sulfobacillus thermosulfidooxidans*	Thermophiles	Acidophiles
GCA_000195575.1	*Carnobacterium_A sp000195575*	Psychrophiles	Alkaliphiles
GCA_900110375.1	*Flavobacterium sinopsychrotolerans*	Psychrophiles	Alkaliphiles
GCA_018861005.1	*Polaribacter vadi_A*	Psychrophiles	Alkaliphiles
GCA_003259835.1	*Flavobacterium aquaticum*	Psychrophiles	Alkaliphiles
GCA_001761365.1	*Polaribacter vadi*	Psychrophiles	Alkaliphiles
GCA_001975665.1	*Polaribacter reichenbachii*	Psychrophiles	Alkaliphiles

B Intra-genus distance details

The intra-genus distances for all genera in the Temperature and pH datasets were calculated. To reduce the influence of extreme values, 10% of the values (5% from the lower end and 5% from the upper end) were removed as outliers. This step was necessary because some metrics, such as LPIPS, are not fully generalizable for FCGRs and can produce a wide range of extreme values. After outlier removal, the threshold for filtering the FCGRs of bacterium-archaeon pairs was set at the 90th percentile of the adjusted distribution. Figure S1, Figure S2, and Figure S3 show the adjusted distance distributions.

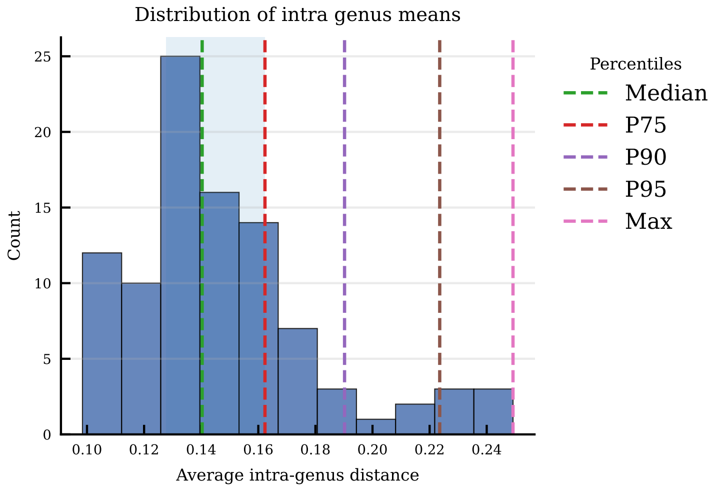

Figure S1: Distribution of intra-genus using Descriptor metric.

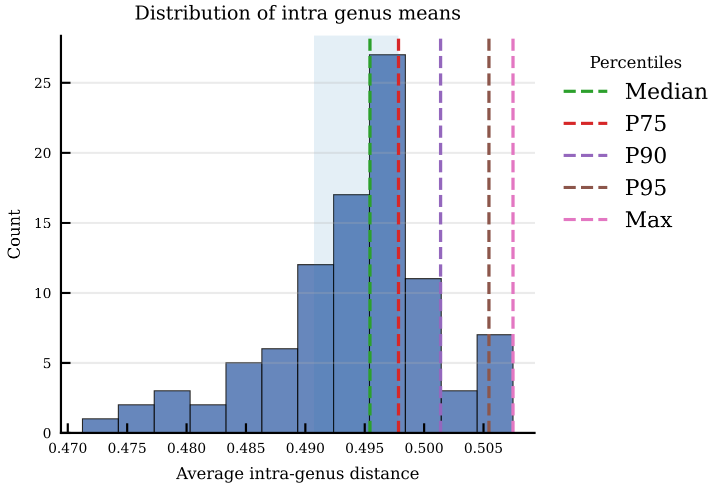

Figure S2: Distribution of intra-genus using DSSIM metric.

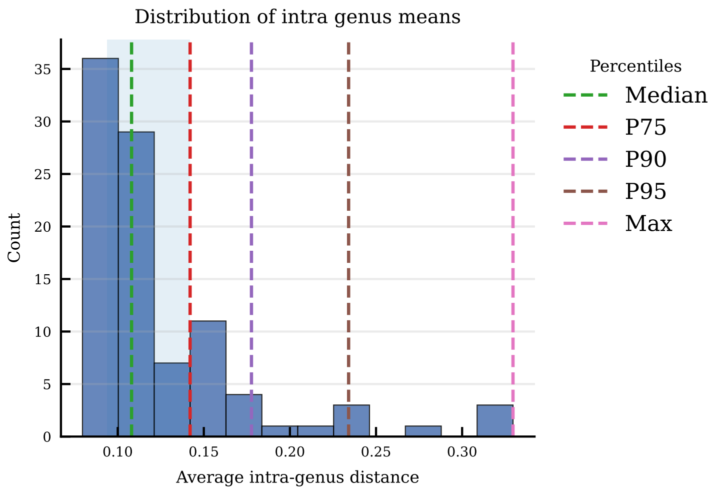

Figure S3: Distribution of intra-genus using LPIPS metric.

C Pervasiveness results for the standard scenario

The random genome proxy was tested for classification using SVM models. This classification was repeated 10 times, each time a 10-fold cross-validation with a new random genome proxy was performed to check the pervasiveness of the genomic signature across the genome. The results of this analysis under the standard scenario are provided in Table S4 and Table S5, for the *Temperature Dataset* and *pH Dataset*, respectively.

Table S4: Maximum average accuracy across six genome proxy lengths in ten repeated SVM classification trials on the *Temperature Dataset* under the standard scenario, for k-mer sizes 1 to 9. The table lists the highest average accuracy for each genome proxy length, alongside the k-mer size that achieved this accuracy and the variance in percentage.

Genome proxy length	Class labelling type	Max average accuracy (%)	Variance (%)	k-value
10 kbp	Taxonomy	99.03	0.0007	5
	Temperature	81.66	0.007	5
50 kbp	Taxonomy	99.41	0.0001	6
	Temperature	84.42	0.005	7
100 kbp	Taxonomy	99.49	0.0001	7
	Temperature	85.06	0.003	7
250 kbp	Taxonomy	99.48	0.0002	7
	Temperature	85.90	0.002	9
500 kbp	Taxonomy	99.51	0.0002	7
	Temperature	86.15	0.004	9
1,000 kbp	Taxonomy	99.51	0.0002	9
	Temperature	86.20	0.0007	9

Table S5: Maximum average accuracy across six genome proxy lengths in ten repeated SVM classification trials on the *pH Dataset* under the standard scenario, for k-mer sizes 1 to 9. The table lists the highest average accuracy for each genome proxy length, alongside the k-mer size that achieved this accuracy and the variance in percentage.

Genome proxy length	Class labelling type	Max average accuracy (%)	Variance (%)	k-value
10 kbp	Taxonomy	97.44	0.002	4
	pH	89.80	0.014	6
50 kbp	Taxonomy	97.80	0.003	7
	pH	91.40	0.006	7
100 kbp	Taxonomy	98.33	0.0008	6
	pH	91.63	0.007	8
250 kbp	Taxonomy	98.32	0.002	8
	pH	92.52	0.007	8
500 kbp	Taxonomy	98.54	0.001	8
	pH	92.59	0.006	9
1,000 kbp	Taxonomy	92.91	0.002	9
	pH	98.43	0.002	9

D Performance of all classification models across all nine k values

The classification of extremophiles using taxonomic and environment-type labels was done using 6 classifiers, 9 different k-mer sizes, and 6 different genome proxy lengths, for both the *Temperature Dataset* and *pH Dataset*, under standard and bias mitigation scenarios. Figure S4 and Figure S5 present the taxonomy classification accuracy for the *Temperature Dataset* under both standard and bias mitigation scenarios. Similarly, Figure S6 and Figure S7 display the taxonomy classification accuracy for the *pH Dataset* under the same scenarios.

Figure S8 and Figure S9 present the environment-type classification accuracy for the *Temperature Dataset* under both standard and bias mitigation scenarios. Similarly, Figure S10 and Figure S11 display the environment-type classification accuracy for the *pH Dataset* under the same scenarios.

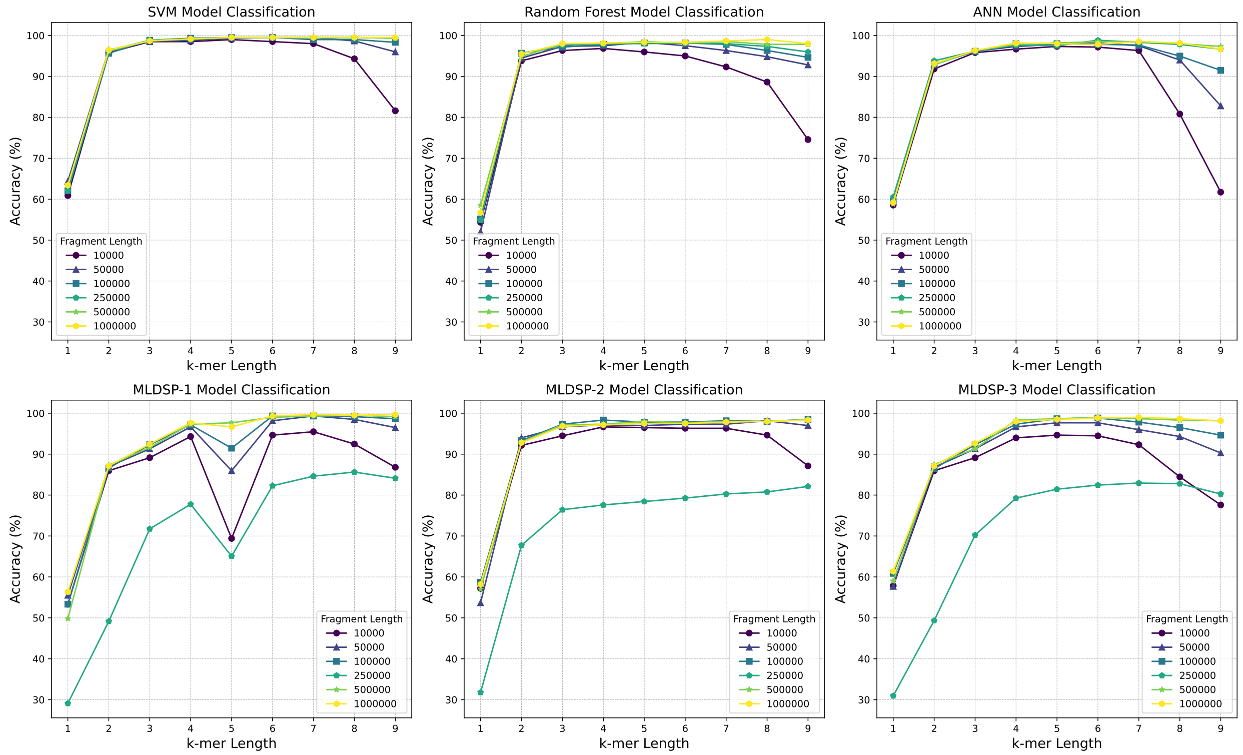

Figure S4: Taxonomic classification accuracy of the *Temperature Dataset* under standard scenario.

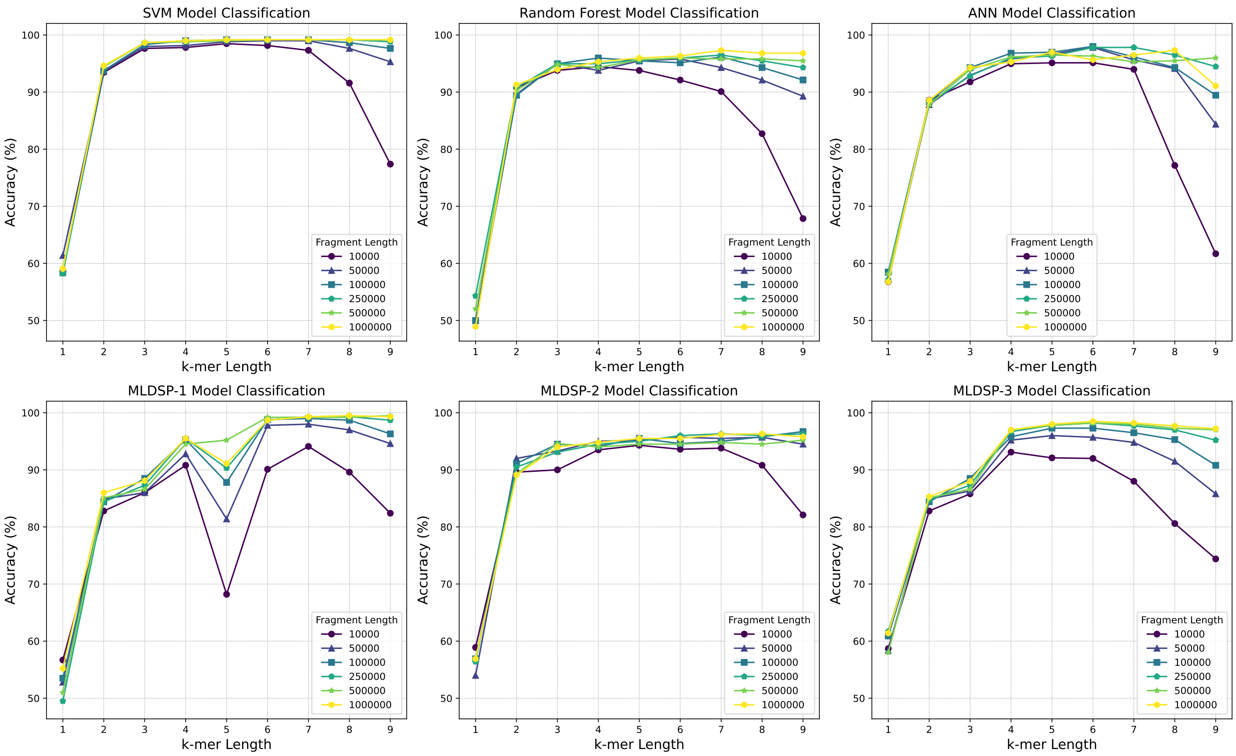

Figure S5: Taxonomic classification accuracy of the *Temperature Dataset* under bias mitigation scenario.

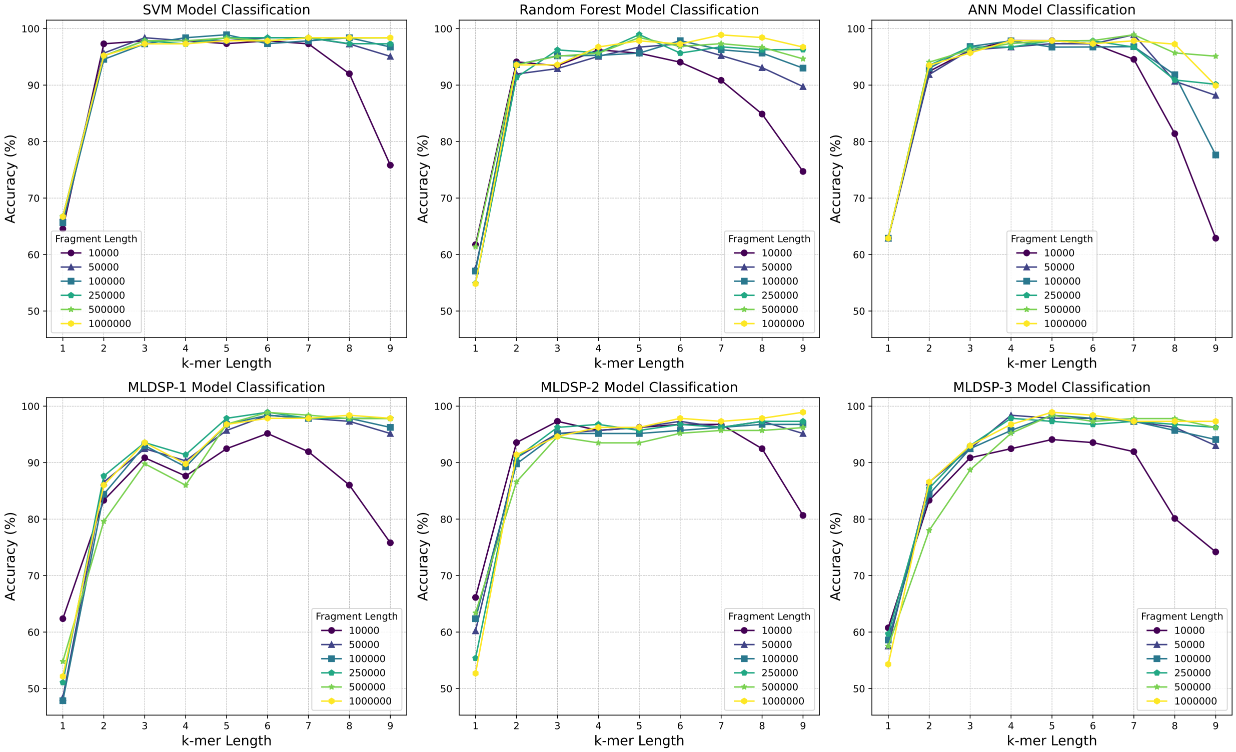

Figure S6: Taxonomic classification accuracy of the *pH Dataset* under standard scenario.

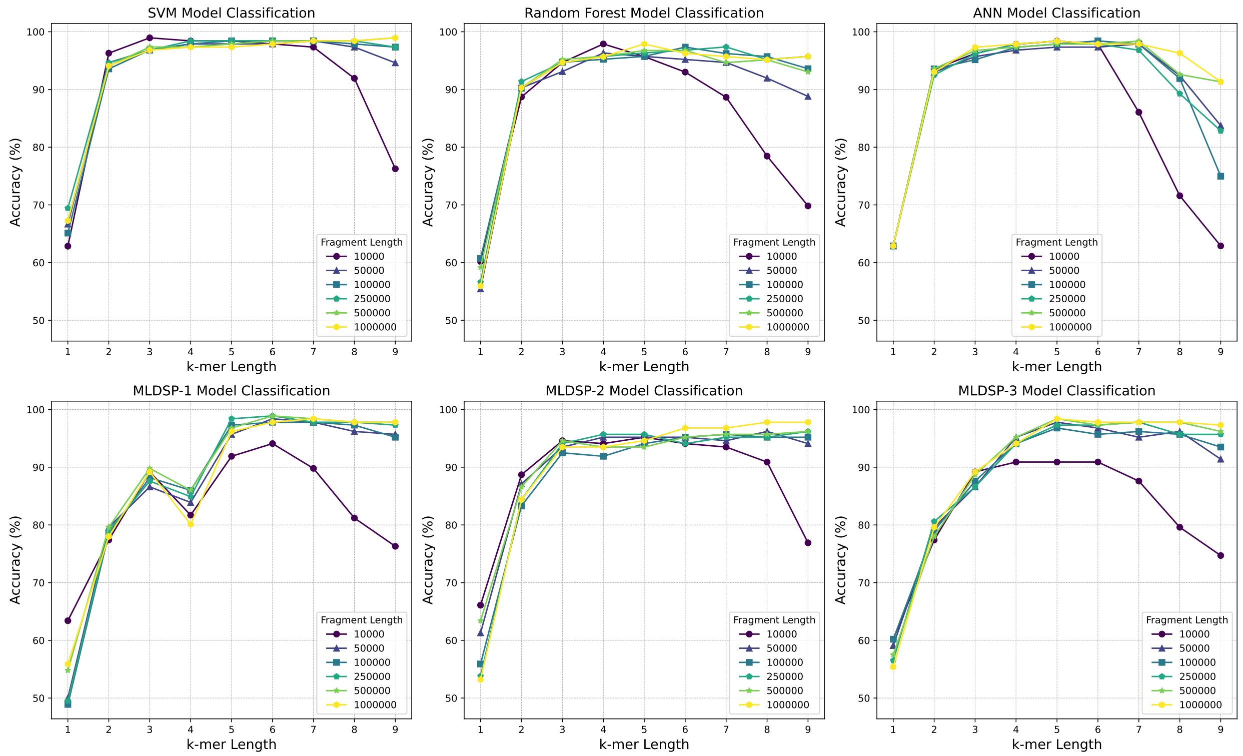

Figure S7: Taxonomic classification accuracy of the *pH Dataset* under bias mitigation scenario.

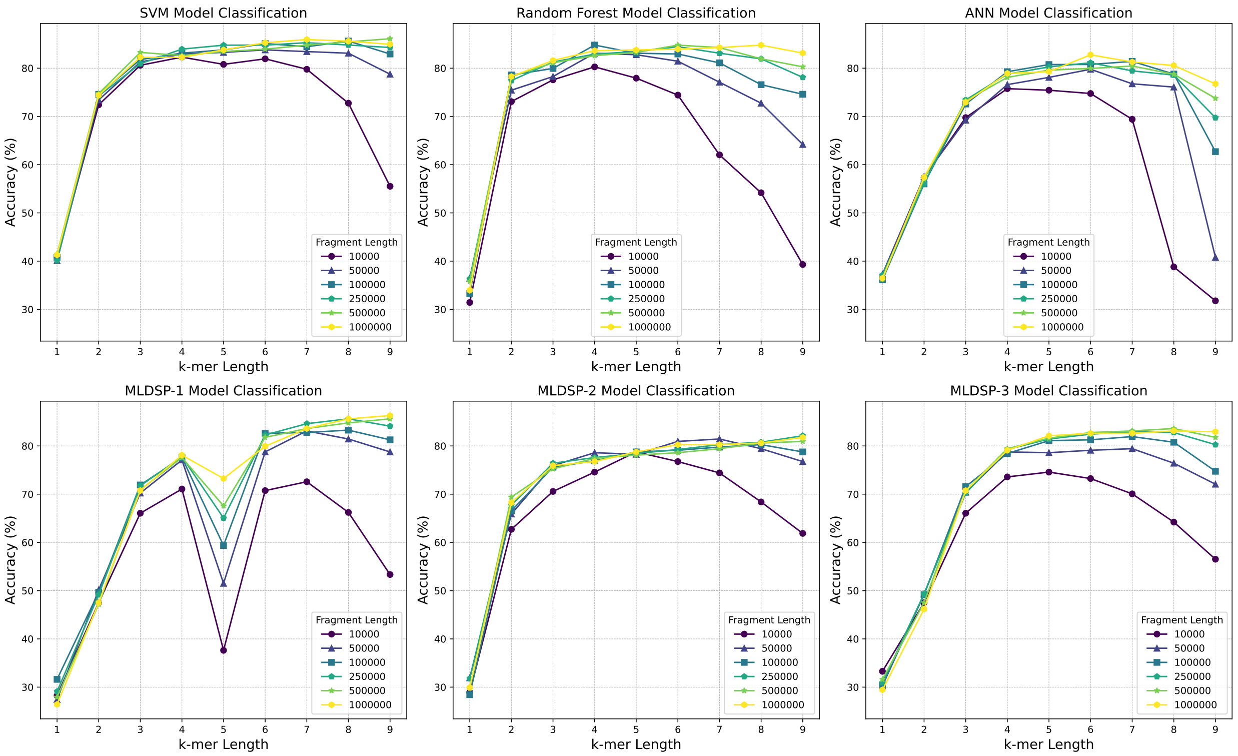

Figure S8: Environment-type classification accuracy of the *Temperature Dataset* under standard scenario.

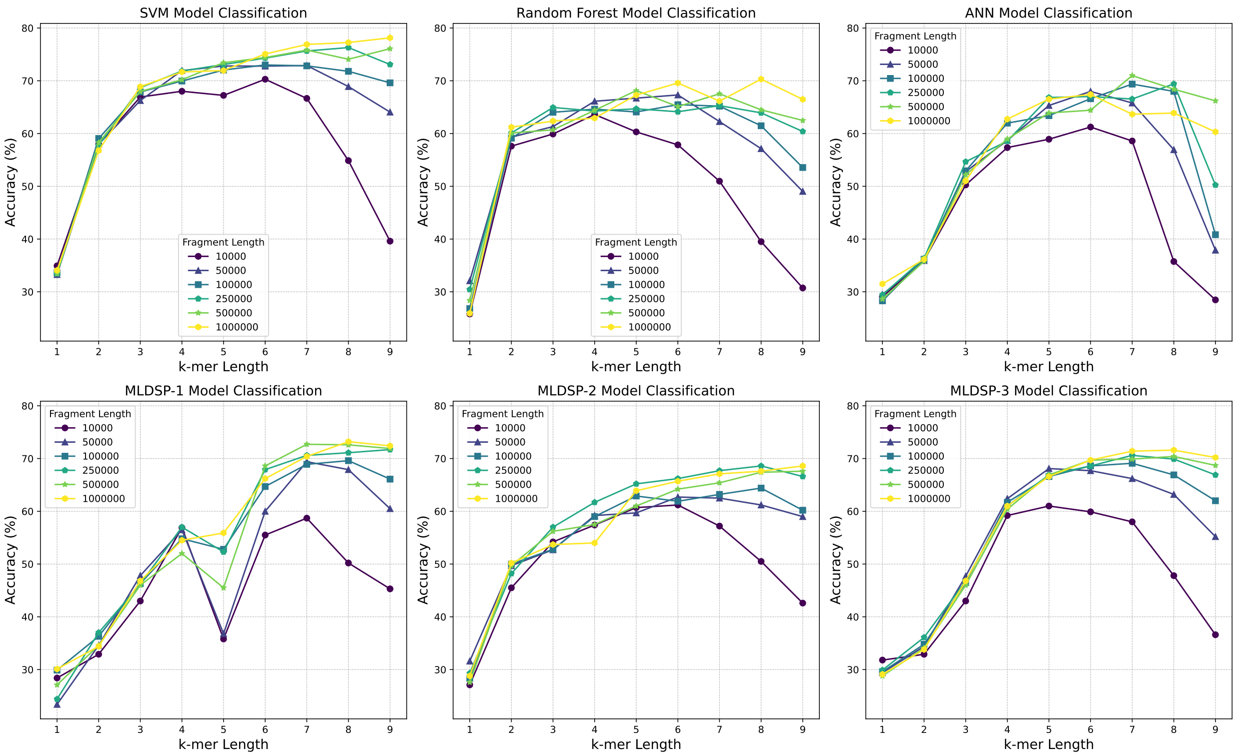

Figure S9: Environment-type classification accuracy of the *Temperature Dataset* under bias mitigation scenario.

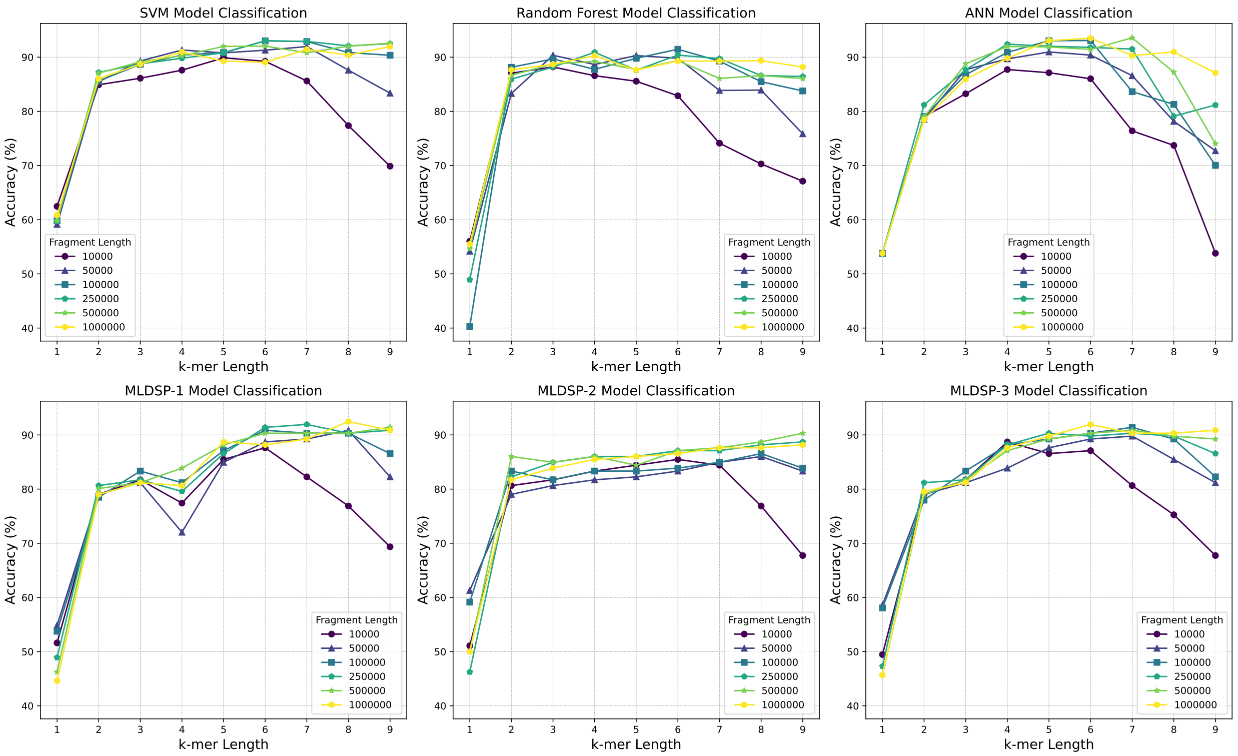

Figure S10: Environment-type classification accuracy of the *pH Dataset* under standard scenario.

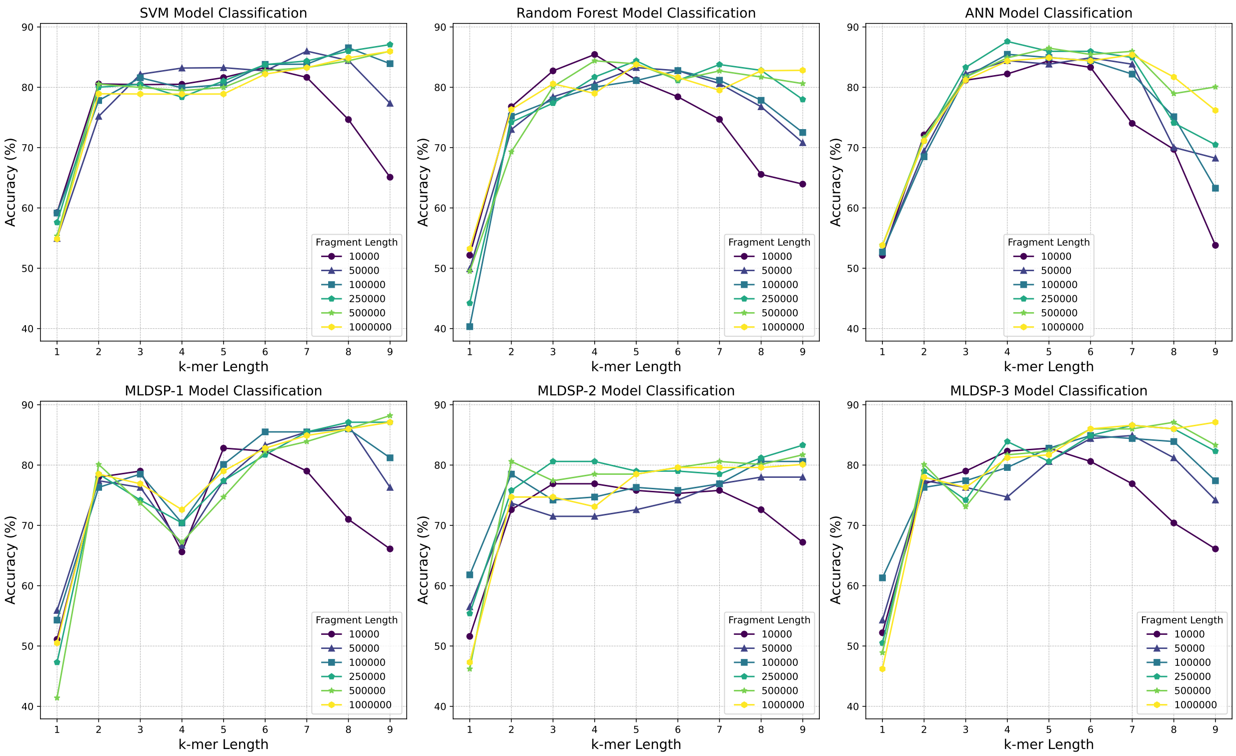

Figure S11: Environment-type classification accuracy of the *pH Dataset* under bias mitigation scenario.

E The detailed results of all classifiers for six genome proxy length and k-mer sizes ranging from 6 to 9

The detailed classification accuracy of all six classifiers for all the genome proxy lengths and k-mer sizes ranging from 6 to 9 for the standard scenario is provided in Table S6, and Table S8, and for the bias mitigation scenario is provided in Table S7, and Table S9.

Table S6: The accuracy of six supervised learning classifiers trained on the *Temperature Dataset*, under the standard scenario, for two different label assignments, taxonomy and environment category, and k values ranging from 6 to 9 and fragment lengths of 10kbp, 50kbp, 100 kbp, 250 kbp, 500 kbp, and 1000 kbp. The classification accuracy was determined through 10-fold cross-validation. The numbers in bold show the highest accuracy for the respective genome proxy length.

Genome proxy length	k-mer	Labelling type	ANN	RF	SVM	MLDSP-1	MLDSP-2	MLDSP-3
**10 kbp**	6	Temperature	74.75	74.42	**81.94**	70.74	76.76	73.24
		Taxonomy	97.15	94.99	**98.50**	94.65	96.32	94.48
	7	Temperature	69.40	62.03	79.77	72.58	74.41	70.07
		Taxonomy	96.31	92.31	98.00	95.48	96.32	92.31
	8	Temperature	38.80	54.17	72.74	66.22	68.39	64.21
		Taxonomy	80.80	88.62	94.31	92.47	94.65	84.45
	9	Temperature	31.77	39.30	55.51	53.34	61.87	56.52
		Taxonomy	61.71	74.57	81.59	86.79	87.12	77.59
**50 kbp**	6	Temperature	79.77	81.44	**83.79**	78.76	80.94	79.10
		Taxonomy	98.16	97.50	**99.50**	98.16	97.32	97.66
	7	Temperature	76.76	77.09	83.44	83.11	81.44	79.43
		Taxonomy	97.49	96.32	**99.50**	99.33	97.32	95.99
	8	Temperature	76.08	72.74	83.10	81.44	79.43	76.42
		Taxonomy	93.99	94.81	98.66	98.49	98.16	94.31
	9	Temperature	40.79	64.19	78.75	78.76	76.76	72.07
		Taxonomy	82.79	92.81	95.99	96.49	96.99	90.30
**100 kbp**	6	Temperature	80.77	82.94	**85.11**	82.61	79.10	81.27
		Taxonomy	97.83	98.16	**99.50**	99.33	97.83	98.83
	7	Temperature	81.41	81.10	84.45	82.78	79.77	81.94
		Taxonomy	97.66	97.82	98.99	99.33	98.16	97.83
	8	Temperature	78.78	76.58	85.62	83.28	80.27	80.77
		Taxonomy	94.96	96.33	99.00	99.16	97.99	96.49
	9	Temperature	62.69	74.59	82.94	81.27	78.76	74.75
		Taxonomy	91.48	94.65	98.33	98.66	98.49	94.65
**250 kbp**	6	Temperature	81.12	84.45	84.78	82.27	79.26	82.44
		Taxonomy	98.83	98.16	99.49	99.00	97.66	99.00
	7	Temperature	79.44	83.10	85.28	84.62	80.27	82.94
		Taxonomy	98.32	98.00	**99.50**	99.16	98.16	98.16
	8	Temperature	78.57	81.94	84.80	**85.62**	80.77	82.78
		Taxonomy	97.82	97.33	**99.50**	99.33	98.66	97.99
	9	Temperature	69.73	78.10	84.29	84.11	82.11	80.27
		Taxonomy	96.66	95.97	99.33	99.16	98.83	96.49
**500 kbp**	6	Temperature	79.94	84.78	83.95	81.77	78.60	82.78
		Taxonomy	98.50	98.17	**99.50**	99.00	97.66	99.00
	7	Temperature	80.45	84.27	84.78	83.61	79.43	83.11
		Taxonomy	98.50	98.50	**99.50**	99.33	97.99	98.66
	8	Temperature	78.75	81.94	85.45	84.78	80.60	83.61
		Taxonomy	97.99	98.30	**99.50**	**99.50**	97.99	98.33
	9	Temperature	73.75	80.28	**86.11**	85.62	80.94	81.77
		Taxonomy	97.32	97.83	99.33	99.16	98.49	98.16
**1,000 kbp**	6	Temperature	82.77	83.94	85.28	79.93	80.27	82.61
		Taxonomy	97.83	98.33	99.50	99.33	97.49	98.83
	7	Temperature	81.29	84.28	85.94	83.61	80.27	82.61
		Taxonomy	98.50	98.66	99.50	**99.67**	97.83	99.00
	8	Temperature	80.56	84.78	85.61	85.62	80.60	83.11
		Taxonomy	98.16	99.00	99.49	99.50	97.99	98.66
	9	Temperature	76.75	83.13	84.95	**86.29**	81.77	82.94
		Taxonomy	96.66	98.00	99.50	**99.67**	98.33	98.16

Table S7: The classification accuracy of six supervised classifiers trained on the *Temperature Dataset*, under the bias mitigation scenario, for two different label assignments, taxonomy and environment category, and k values ranging from 6 to 9 and fragment lengths of 10 kbp, 50kbp, 100 kbp, 250 kbp, 500 kbp, and 1000 kbp. The classification accuracy was determined through 10-fold cross-validation. The numbers in bold show the highest accuracy for the respective genome proxy length.

**Genome proxy length**	**k-mer**	**Labelling type**	**ANN**	**RF**	**SVM**	**MLDSP-1**	**MLDSP-2**	**MLDSP-3**
**10 kbp**	6	Temperature	61.23	57.85	**70.29**	55.50	61.20	59.90
		Taxonomy	95.13	92.11	**98.15**	90.10	93.60	92.00
	7	Temperature	58.59	50.97	66.66	58.70	57.20	58.00
		Taxonomy	93.98	90.10	97.31	94.10	93.80	88.00
	8	Temperature	35.74	39.51	54.87	50.20	50.50	47.80
		Taxonomy	77.15	82.71	91.58	89.60	90.80	80.60
	9	Temperature	28.47	30.73	39.60	45.30	42.60	36.60
		Taxonomy	61.69	67.84	77.38	82.40	82.10	74.40
**50 kbp**	6	Temperature	67.98	67.31	72.78	60.00	62.70	67.70
		Taxonomy	97.82	95.80	**98.99**	97.80	95.70	95.70
	7	Temperature	65.79	62.27	**72.89**	69.40	62.50	66.20
		Taxonomy	95.63	94.29	**98.99**	98.00	95.50	94.80
	8	Temperature	56.93	57.13	68.90	67.90	61.20	63.20
		Taxonomy	94.13	92.11	97.65	97.00	95.70	91.50
	9	Temperature	37.89	49.03	64.06	60.50	59.00	55.20
		Taxonomy	84.37	89.27	95.30	94.60	94.50	85.80
**100 kbp**	6	Temperature	66.60	65.47	**74.77**	64.70	61.90	68.60
		Taxonomy	97.99	95.13	**99.16**	98.80	94.60	97.30
	7	Temperature	69.37	65.19	72.83	68.90	63.20	69.10
		Taxonomy	96.15	96.15	**99.16**	99.00	95.00	96.50
	8	Temperature	67.99	61.47	71.79	69.60	64.40	66.90
		Taxonomy	94.30	94.30	98.65	98.70	95.80	95.30
	9	Temperature	40.83	53.56	69.61	66.10	60.20	62.00
		Taxonomy	89.45	92.12	97.65	96.30	96.70	90.80
**250 kbp**	6	Temperature	67.01	64.13	**74.29**	67.90	66.20	68.60
		Taxonomy	97.81	95.97	99.16	98.70	96.00	98.20
	7	Temperature	66.54	65.29	75.65	70.60	67.70	70.60
		Taxonomy	97.82	96.48	99.16	**99.30**	96.30	97.70
	8	Temperature	69.41	63.92	76.29	71.10	68.60	69.90
		Taxonomy	96.49	95.48	99.16	**99.30**	96.00	97.00
	9	Temperature	50.24	60.39	73.09	71.70	66.60	66.90
		Taxonomy	94.47	94.30	98.82	98.70	96.30	95.20
**500 kbp**	6	Temperature	64.42	65.11	74.40	68.60	64.20	69.70
		Taxonomy	96.30	95.97	99.16	99.20	94.50	98.20
	7	Temperature	70.99	67.53	75.82	72.70	65.40	69.90
		Taxonomy	95.30	95.81	99.16	99.20	94.80	98.00
	8	Temperature	68.40	64.50	74.08	72.60	67.40	70.40
		Taxonomy	95.48	95.81	99.16	99.20	94.50	97.30
	9	Temperature	66.20	62.48	**76.08**	71.90	67.60	68.70
		Taxonomy	95.98	95.47	99.16	**99.50**	95.20	97.00
**1,000 kbp**	6	Temperature	67.41	69.58	75.08	66.20	65.70	69.70
		Taxonomy	95.64	96.31	99.16	98.70	95.50	98.50
	7	Temperature	63.66	66.14	76.89	70.40	67.10	71.40
		Taxonomy	96.48	97.31	99.16	99.30	96.20	98.20
	8	Temperature	63.88	70.31	77.25	73.20	67.60	71.60
		Taxonomy	97.32	96.81	99.16	**99.50**	96.30	97.70
	9	Temperature	60.32	66.48	**78.14**	72.40	68.60	70.20
		Taxonomy	91.08	96.81	99.16	99.30	95.80	97.20

Table S8: The accuracy of six supervised learning classifiers trained on the *pH Dataset*, under the standard scenario, for two different label assignments, taxonomy and environment category, and k values ranging from 6 to 9 and fragment lengths of 10kbp, 50kbp, 100 kbp, 250 kbp, 500 kbp, and 1000 kbp. The classification accuracy was determined through 10-fold cross-validation. The numbers in bold show the highest accuracy for the respective fragment length.

**Genome proxy length**	**k-mer**	**Labelling type**	**ANN**	**RF**	**SVM**	**MLDSP-1**	**MLDSP-2**	**MLDSP-3**
**10 kbp**	6	pH	86.02	82.87	**89.24**	87.63	85.48	87.10
		Taxonomy	97.34	94.06	**97.84**	95.16	96.77	93.55
	7	pH	76.40	74.12	85.61	82.26	84.41	80.65
		Taxonomy	94.56	90.85	97.31	91.94	96.77	91.94
	8	pH	73.71	70.29	77.37	76.88	76.88	75.27
		Taxonomy	81.40	84.88	92.02	86.02	92.47	80.11
	9	pH	53.80	67.11	69.88	69.35	67.74	67.74
		Taxonomy	62.89	74.71	75.82	75.81	80.65	74.19
**50 kbp**	6	pH	90.38	89.80	91.29	88.71	83.33	89.25
		Taxonomy	97.31	97.31	98.39	98.39	97.31	97.85
	7	pH	86.58	83.86	**91.96**	89.25	84.95	89.78
		Taxonomy	**98.92**	95.23	98.39	97.85	96.24	97.31
	8	pH	78.16	83.92	87.60	90.86	86.02	85.48
		Taxonomy	90.67	93.10	97.28	97.31	97.31	96.24
	9	pH	72.72	75.85	83.36	82.26	83.33	81.18
		Taxonomy	88.19	89.74	95.09	95.16	95.16	93.01
**100 kbp**	6	pH	**93.07**	91.46	93.01	90.86	83.87	90.32
		Taxonomy	96.75	97.87	97.34	**98.39**	95.70	97.85
	7	pH	83.63	89.27	92.89	90.32	84.95	91.40
		Taxonomy	96.81	96.26	97.84	97.85	96.24	97.31
	8	pH	81.29	85.47	90.85	90.32	86.56	89.25
		Taxonomy	91.84	95.67	**98.39**	97.85	96.77	95.70
	9	pH	70.03	83.77	90.35	86.56	83.87	82.26
		Taxonomy	77.63	93.01	96.75	96.24	96.77	94.09
**250 kbp**	6	pH	91.81	90.38	**93.01**	91.40	87.10	89.78
		Taxonomy	97.84	95.67	98.39	**98.92**	96.77	96.77
	7	pH	91.49	89.74	92.92	91.94	87.10	90.32
		Taxonomy	96.73	96.78	98.39	97.85	96.24	97.31
	8	pH	79.09	86.61	92.08	90.32	88.17	89.78
		Taxonomy	90.91	96.26	97.34	97.85	97.31	96.77
	9	pH	81.17	86.43	92.49	90.86	88.71	86.56
		Taxonomy	90.15	96.29	97.31	97.85	97.31	96.24
**500 kbp**	6	pH	91.43	89.36	92.05	90.32	87.10	90.32
		Taxonomy	97.89	96.84	97.84	98.39	97.31	97.85
	7	pH	**93.57**	86.08	90.82	90.32	87.63	90.86
		Taxonomy	**98.92**	97.34	98.36	98.39	97.31	97.85
	8	pH	87.22	86.61	92.02	90.32	88.71	89.78
		Taxonomy	95.70	96.70	98.33	97.85	97.31	97.85
	9	pH	74.04	86.05	92.57	91.40	90.32	89.25
		Taxonomy	95.12	94.65	98.36	97.85	97.31	96.77
**1,000 kbp**	6	pH	**93.51**	89.30	89.06	88.17	86.56	91.94
		Taxonomy	97.34	97.28	97.84	97.85	97.85	98.39
	7	pH	90.29	89.27	91.43	89.25	87.63	90.32
		Taxonomy	97.84	98.89	98.39	97.85	97.31	97.31
	8	pH	90.96	89.36	90.38	92.47	87.63	90.32
		Taxonomy	97.25	98.42	98.39	98.39	97.85	97.31
	9	pH	87.11	88.19	91.96	90.86	88.17	90.86
		Taxonomy	89.91	96.73	98.39	97.85	**98.92**	97.31

Table S9: The accuracy of six supervised learning classifiers trained on the *pH Dataset*, under the bias mitigation scenario, for two different label assignments, taxonomy and environment category, and k values ranging from 6 to 9 and fragment lengths of 10kbp, 50kbp, 100 kbp, 250 kbp, 500 kbp, and 1000 kbp. The classification accuracy was determined through 10-fold cross-validation. The numbers in bold show the highest accuracy for the respective genome proxy length.

Genome proxy length	k-value	Labelling type	ANN	RF	SVM	MLDSP-1	MLDSP-2	MLDSP-3
**10 kbp**	6	pH	**83.30**	78.42	83.27	82.30	75.30	80.60
		Taxonomy	97.87	93.01	**97.89**	94.10	94.10	90.90
	7	pH	74.01	74.68	81.64	79.00	75.80	76.90
		Taxonomy	86.05	88.65	97.34	89.80	93.50	87.60
	8	pH	69.71	65.56	74.65	71.00	72.60	70.40
		Taxonomy	71.58	78.45	91.93	81.20	90.90	79.60
	9	pH	53.80	63.95	65.09	66.10	67.20	66.10
		Taxonomy	62.89	69.82	76.26	76.30	76.90	74.70
**50 kbp**	6	pH	84.88	82.75	82.72	83.30	74.20	84.40
		Taxonomy	97.34	95.20	97.89	98.40	95.20	96.80
	7	pH	83.83	80.61	85.99	85.50	76.90	84.90
		Taxonomy	97.89	94.68	**98.42**	97.80	94.60	95.20
	8	pH	70.03	76.78	84.44	**86.60**	78.00	81.20
		Taxonomy	92.40	91.96	97.34	96.20	96.20	96.20
	9	pH	68.25	70.82	77.34	76.30	78.00	74.20
		Taxonomy	83.74	88.80	94.62	95.70	94.10	91.40
**100 kbp**	6	pH	84.39	82.78	83.80	85.50	75.80	84.90
		Taxonomy	**98.42**	97.34	**98.42**	97.80	95.20	95.70
	7	pH	82.19	81.17	83.83	85.50	76.90	84.40
		Taxonomy	97.87	96.23	**98.42**	97.80	95.70	96.20
	8	pH	75.12	77.84	**86.55**	86.00	80.60	83.90
		Taxonomy	91.90	95.70	97.89	97.30	95.20	95.70
	9	pH	63.27	72.51	83.92	81.20	80.60	77.40
		Taxonomy	74.97	93.60	97.34	95.20	95.20	93.50
**250 kbp**	6	pH	85.96	81.14	83.77	81.70	79.00	84.90
		Taxonomy	97.87	96.81	98.42	**98.90**	94.10	97.30
	7	pH	84.94	83.77	84.36	85.50	78.50	86.60
		Taxonomy	96.81	97.37	98.42	97.80	95.20	97.80
	8	pH	74.09	82.81	85.99	**87.10**	81.20	86.00
		Taxonomy	89.27	95.18	98.42	97.80	95.20	95.70
	9	pH	70.47	77.98	87.08	**87.10**	83.30	82.30
		Taxonomy	82.84	95.73	97.34	97.30	96.20	95.70
**500 kbp**	6	pH	85.44	81.20	82.75	82.30	79.60	86.00
		Taxonomy	97.87	96.78	98.42	98.90	95.20	97.30
	7	pH	85.96	82.72	83.27	83.90	80.60	86.00
		Taxonomy	98.39	94.65	98.42	98.40	95.70	97.80
	8	pH	78.95	81.70	84.39	86.00	80.10	87.10
		Taxonomy	92.60	95.15	98.42	97.80	95.70	97.80
	9	pH	80.06	80.61	85.99	**88.20**	81.70	83.30
		Taxonomy	91.32	93.04	**98.95**	97.80	96.20	96.20
**1,000 kbp**	6	pH	84.39	81.64	82.19	82.80	79.60	86.00
		Taxonomy	97.87	96.26	97.87	97.80	96.80	97.80
	7	pH	85.38	79.50	83.25	84.90	79.60	86.60
		Taxonomy	97.87	95.70	98.42	98.40	96.80	97.80
	8	pH	81.70	82.75	84.88	86.00	79.60	86.00
		Taxonomy	96.29	95.15	98.42	97.80	97.80	97.80
	9	pH	76.17	82.81	85.96	**87.10**	80.10	**87.10**
		Taxonomy	91.35	95.73	**98.95**	97.80	97.80	97.30

F Confirmed candidate pairs list

The list of the IDs and the species name of the pairs after the FCGR filtering is provided in Table S10.

Table S10: List of the confirmed candidate pairs. The first 15 pairs are the environment-related pairs that passed the hypothesis testing.

Index	Confirmed candidate pair NCBI ID	Bacterium	Archaeon
1	GCA_000016785.1_GCA_000789255.1	*Thermotoga petrophila*	*Geoglobus acetivorans*
2	GCA_003568865.1_GCA_004102725.1	*Rubrobacter indicoceani*	*Methanoculleus taiwanensis*
3	GCA_003568865.1_GCA_017873855.1	*Rubrobacter indicoceani*	*Methanolinea mesophila*
4	GCA_003568865.1_GCA_900095385.1	*Rubrobacter indicoceani*	*Methanoculleus chikugoensis*
5	GCA_000512735.1_GCA_000211475.1	*Thermocrinis ruber*	*Pyrococcus furiosus*
6	GCA_000504085.1_GCA_000214725.1	*Pseudothermotoga elfii*	*Methanobacterium paludis*
7	GCA_000512735.1_GCA_000246985.3	*Thermocrinis ruber*	*Thermococcus litoralis*
8	GCA_003568865.1_GCA_000304355.2	*Rubrobacter indicoceani*	*Methanoculleus bourgensis*
9	GCA_000512735.1_GCA_000446015.1	*Thermocrinis ruber*	*Thermofilum adornatum*
10	GCA_000504085.1_GCA_000969905.1	*Pseudothermotoga elfii*	*Methanosarcina vacuolata*
11	GCA_000512735.1_GCA_000725425.1	*Thermocrinis ruber*	*Palaeococcus pacificus*
12	GCA_000512735.1_GCA_002214605.1	*Thermocrinis ruber*	*Thermococcus chitonophagus*
13	GCA_003568865.1_GCA_001571405.1	*Rubrobacter indicoceani*	*Methanoculleus thermophilus*
14	GCA_003568865.1_GCA_001602375.1	*Rubrobacter indicoceani*	*Methanoculleus horonobensis*
15	GCA_000145615.1_GCA_000317795.1	*Thermoanaerobacterium thermosaccharolyticum*	Caldisphaera lagunensis
16 *	GCA_000147695.3_GCA_000166095.1	*Thermoanaerobacter wiegelii*	*Methanothermus fervidus*
17 *	GCA_000212395.1_GCA_000166095.1	*Thermodesulfobium narugense*	*Methanothermus fervidus*
18 *	GCA_003722315.1_GCA_000166095.1	*Thermoanaerobacter ethanolicus*	*Methanothermus fervidus*
19	GCA_900128965.1_GCA_000018365.1	*Ferrithrix thermotolerans*	*Thermococcus onnurineus*
20	GCA_000025605.1_GCA_000243315.1	*Thermocrinis albus*	*Metallosphaera yellowstonensis*
21	GCA_000025605.1_GCA_013343295.1	*Thermocrinis albus*	*Metallosphaera tengchongensis*
22	GCA_000145615.1_GCA_000166095.1	*Thermoanaerobacterium thermosaccharolyticum*	*Methanothermus fervidus*
23	GCA_000512735.1_GCA_000151105.2	*Thermocrinis ruber*	*Thermococcus barophilus*
24	GCA_900129115.1_GCA_000166095.1	*Thermoanaerobacter uzonensis*	*Methanothermus fervidus*
25	GCA_000430585.1_GCA_000698785.1	*Alicyclobacillus herbarius*	*Nitrososphaera viennensis*
26	GCA_000444055.1_GCA_000698785.1	*Alicyclobacillus acidoterrestris*	*Nitrososphaera viennensis*
27	GCA_001447355.1_GCA_000698785.1	*Alicyclobacillus tengchongensis*	*Nitrososphaera viennensis*
28	GCA_001552255.1_GCA_000698785.1	*Alicyclobacillus shizuokensis*	*Nitrososphaera viennensis*
29	GCA_017298635.1_GCA_000698785.1	*Alicyclobacillus ferrooxydans*	*Nitrososphaera viennensis*
30	GCA_900176145.1_GCA_000698785.1	*Sulfobacillus thermosulfidooxidans*	*Nitrososphaera viennensis*
31	GCA_003259835.1_GCA_002945325.1	*Flavobacterium aquaticum*	*Methanococcus maripaludis*
32	GCA_003350545.1_GCA_002945325.1	*Flavobacterium glaciei*	*Methanococcus maripaludis*
33	GCA_003634755.1_GCA_002945325.1	*Flavobacterium limicola*	*Methanococcus maripaludis*
34	GCA_015223105.1_GCA_002945325.1	*Flavobacterium aquaticum*	*Methanococcus maripaludis*
35	GCA_900099915.1_GCA_002945325.1	*Flavobacterium omnivorum*	*Methanococcus maripaludis*
36	GCA_900106645.1_GCA_002945325.1	*Flavobacterium degerlachei*	*Methanococcus maripaludis*
37	GCA_900108015.1_GCA_002945325.1	*Flavobacterium urumqiense*	*Methanococcus maripaludis*
38	GCA_900110375.1_GCA_002945325.1	*Flavobacterium sinopsychrotolerans*	*Methanococcus maripaludis*
39	GCA_900129575.1_GCA_002945325.1	*Flavobacterium segetis*	*Methanococcus maripaludis*
40	GCA_900142885.1_GCA_002945325.1	*Flavobacterium xinjiangense*	*Methanococcus maripaludis*

* Pairs 16, 17, and 18 also passed hypothesis testing and could potentially be considered as environment-related pairs. However, during our final checks, we noticed that NCBI had recently changed the status of the *Methanothermus fervidus* reference genome to “suppressed” due to the lack of annotation. Since no updated version of this genome is currently available on NCBI, we chose not to include it in the final set of pairs to ensure complete confidence in our results. It is worth noting, however, that this species is not entirely excluded from our datasets, as our study is alignment-free and therefore not affected by the absence of annotation.

G Phenotypic traits of environment-related pairs

In our study, we studied the phenotypic traits of the species of the confirmed pairs groups, highlighting key attributes such as pH range, temperature, salinity, and cell shape. The Tables Table S11, Table S12, Table S13, Table S14, and Table S15 show the details of this study for Groups 1 to 5, respectively. The tables also include geochemical details of the environments from which the microbes were isolated.

Table S11: Characterizing phenotypic traits of species in Group 1 and their isolating environment. The table describes various phenotypic traits attributed to each of the species and the geochemical information associated with the environments the microbes were initially isolated. The optimized growth range(s), if known, are described in parentheses. Phenotypic trait categories lacking information for a given species are denoted with "—".

**Species**	***Thermoanaerobacterium*** [1, 2]	***Caldisphaera lagunensis*** [3]
**Domain**	Bacteria	Archaea
**pH range**	4.1-7.6 (5-5.25)	2.3-5.4 (3.75)
**Temperature**	60°C	45-80°C (72.5°C)
**Salinity**	0-2.5% NaCl	0-1.5% NaCl
**Cell shape**	Long slender granulated bacilli	Mostly regular cocci
**Gram stain**	Positive	Negative
**Motility**	Peritrichous flagella	Non-motile
**S-Layer protein composition**	S-layer lattice	P3-Symmetry layer lattice
**Oxygen tolerance**	Anaerobic	Anaerobic
**Genome size**	2.8 Mb	1.5 Mb
**Intergenic sequence content**	Pseudogenes present	Pseudogenes present
**Geographic source**	Derived from Austrian beet sugar factory	Derived from Mud Spring Mt Maquiling Laguna Philippines
**Geochemical parameters**	Observed in geothermal hot springs	Observed in volcanic acidic hot springs

Table S12: Characterizing phenotypic traits of species in Group 2 and their isolating environment. The table describes various phenotypic traits attributed to each of the species, and the geochemical information associated with the environments the microbes were initially isolated. The optimized growth range(s), if known, are described in parentheses. Phenotypic trait categories lacking information for a given species are denoted with "—".

**Species**	***Thermotoga petrophila***[4]	***Geoglobus acetivorans***[5]
**Domain**	Bacteria	Archaea
**pH range**	5.2-9.0 (7.0)	5.0-7.5 (6.8)
**Temperature**	47-88°C (80°C)	50-85°C (81°C)
**Salinity**	0.1-5.5% NaCl (1.0% NaCl)	1.0-6.0% NaCl (2.5% NaCl)
**Cell shape**	Rods (bacilli)	Regular to irregular cocci
**Gram stain**	Negative	Negative
**Motility**	Subpolar and lateral flagella	Non-motile
**S-Layer protein composition**	S-layer lattice	S-layer lattice
**Oxygen tolerance**	Anaerobic	Anaerobic
**Genome size**	1.8 Mb	1.9 Mb
**Intergenic sequence content**	Pseudogenes present	Pseudogenes present
**Geographic source**	Isolated from production fluid in the Kubiki oil reservoir, Niigata prefecture, Japan	Isolated from Ashadze hydrothermal field, Mid-Atlantic Ridge
**Geochemical parameters**	Subterranean starved conditions	Black smoker field at depth of 4100 m

Table S13: Characterizing phenotypic traits of species in Group 3 and their isolating environment. The table describes various phenotypic traits attributed to each of the species and the geochemical information associated with the environments the microbes were initially isolated. The optimized growth range(s), if known, are described in parentheses. Phenotypic trait categories lacking information for a given species are denoted with "—".

**Species**	***Thermocrinis ruber***[6, 7]	***Pyrococcus furiosus***[8, 9]	***Thermofilum adornatum***[10, 11]	***Palaeococcus pacificus***[12]	***Thermococcus chitonophagus***[13, 14]	***Thermococcus litoralis***[15, 16]
**Domain**	Bacteria	Archaea	Archaea	Archaea	Archaea	Archaea
**pH range**	7.0-8.5	5.0-9.0	5.3-8.5	5.0-8.0	3.5-9	6.0-8.5
**Temperature**	44-89°C (80°C)	70-100°C (93°C)	50-95°C (80°C)	50-90°C (80°C)	60-93°C (85°C)	55-98°C (88°C)
**Salinity**	0-0.4% NaCl	0.5-5% NaCl	0-2.5% NaCl	1-4% NaCl (3% NaCl)	1.8-6.5% NaCl (2.5% NaCl)	1.8-6.5% NaCl (2.5% NaCl)
**Cell shape**	Rod-shaped cells	Slightly irregular cocci	Filamentous bacilli	Irregular cocci	Round to irregular cocci	Round to irregular cocci
**Gram stain**	Negative	Negative	Negative	Negative	Negative	Negative
**Motility**	Monopolar polytrichous flagella	Monopolar polytrichous flagella	Monopolar polytrichous flagella	Monopolar polytrichous flagella	Monopolar polytrichous flagella	Non-flagellated, non-motile
**S-Layer protein composition**	No evidence of a regularly arrayed SLP	Hexagonal lattice	—	—	Hexagonal lattice	Hexagonal lattice
**Oxygen tolerance**	Aerobic	Anaerobic	Anaerobic	Anaerobic	Anaerobic	Anaerobic
**Genome size**	1.52 Mb	1.89 Mb	1.75 Mb	1.9 Mb	1.95 Mb	1.82 Mb
**Intergenic sequence content**	Pseudogenes present	Pseudogenes and IS elements present	No pseudogenes	No pseudogenes	Pseudogenes and IS elements present	Pseudogenes and IS elements present
**Geographic source**	Octopus Spring Yellowstone National Park WY USA	Submarine solfataric field in the bay of Porto di Levante Vulcano Island Italy	Isolated from a Kamchatkan (Siberian) hot spring in 2009	Isolated from a deep-sea hydrothermal vent field at a depth of 2737 m at the Niaochao site on the East Pacific Ocean Rise	Smoker Site Guaymas Basin Gulf of California Mexico	Shallow submarine solfataras near the beach of Lucrino Bay of Naples, Italy
**Geochemical parameters**	Evidence for hydrothermal petroleum-like substances in other hot springs atop the caldera	Evidence for gas discharges containing light hydrocarbons	Solfataric hot spring (sulphur-containing gas exhaust)	Geothermally heated marine sediments 2773 m	Evidence for gas discharges containing petroleum-like hydrocarbons	Evidence for gas discharges containing light hydrocarbons

Table S14: Characterizing phenotypic traits of species in Group 4 and their isolating environment. The table describes various phenotypic traits attributed to each of the species, and the geochemical information associated with the environments the microbes were initially isolated. The optimized growth range(s), if known, are described in parentheses. Phenotypic trait categories lacking information for a given species are denoted with "—".

**Species**	***Pseudothermotoga elfii***[17]	***Methanobacterium paludis***[18]	***Methanosarcina vacuolata***[19]
**Domain**	Bacteria	Archaea	Archaea
**pH range**	5.5–7.5	4.8–6.6	6.0–8.0
**Temperature**	66°C	16–40°C	18–42°C
**Salinity**	0.0–2.8% NaCl	—	—
**Cell shape**	Regular bacilli	Regular bacilli	Irregular cocci
**Gram stain**	Negative	Negative	Positive
**Motility**	Peritrichous flagella	Non-motile	Non-motile
**S-Layer protein composition**	SLPs present	—	—
**Oxygen tolerance**	Anaerobic	Anaerobic	Anaerobic
**Genome size**	2.2 Mb	2.5 Mb	4.6 Mb
**Intergenic sequence content**	Pseudogenes present	Pseudogenes present	Pseudogenes present
**Geographic source**	Isolated from an oil-producing well in Africa	Isolated from peat soil near Anchorage, Alaska	Isolated from an anaerobic digestor in the former USSR
**Geochemical parameters**	Deep subsurface environment	Described as "hydrogenotrophic, methanogen(ic)"	Initially found in sludge of methane tank; also found in wetlands and swamps

Table S15: Characterizing phenotypic traits of species in Group 5 and their isolating environment. The table describes phenotypic traits attributed to each species and the geochemical information associated with the environments from which the microbes were initially isolated. Optimized growth ranges, if known, are shown in the pH, temperature, and salinity rows. Phenotypic trait categories lacking information for a given species are denoted with "—".

**Species**	***Rubrobacter indicoceani***[20]	***Methanoculleus chikugoensis***[21]	***Methanoculleus bourgensis***[22, 23]	***Methanolinea mesophila***[24]	***Methanoculleus horonobensis***[25]	***Methanoculleus taiwanensis***[26]	***Methanoculleus thermophilus***[27, 28]
**Domain**	Bacteria	Archaea	Archaea	Archaea	Archaea	Archaea	Archaea
**pH range**	7.0–8.0	6.7–7.2	6.8–7.0	6.5–7.4	5.8–8.2	8.1	—
**Temperature**	20–37°C	25–30°C	37°C	20–40°C	25–45°C	37°C	55°C
**Salinity**	1.0–5.0% NaCl	0.1 M NaCl	—	0.0–0.025% NaCl	0.0–1.3% NaCl	0.0–0.1% NaCl	—
**Cell shape**	Short bacilli	Irregular cocci	—	Filamentous cocci	Irregular cocci	Irregular cocci	Irregular cocci
**Gram stain**	Positive	Negative	Negative	Negative	Negative	Negative	Negative
**Motility**	Non-motile	Non-motile flagella	—	Non-motile	Non-motile	Non-motile	—
**S-Layer protein composition**	—	—	—	—	—	SLPs present	—
**Oxygen tolerance**	Aerobic	Anaerobic	Anaerobic	Anaerobic	Anaerobic	Anaerobic	Anaerobic
**Genome size**	3.07 Mb	2.6 Mb	2.79 Mb	2.7 Mb	2.4 Mb	2.8 Mb	2.2 Mb
**Intergenic sequence content**	Pseudogenes present	Pseudogenes present	Pseudogenes present	Pseudogenes present	Pseudogenes present	Pseudogenes present	Pseudogenes present
**Geographic source**	Deep-sea sediment, Indian Ocean	isolated from paddy field soil in Chikugo, Fukuoka, Japan	Sewage, sludge digestor	Rice field soil, Taiwan	Groundwater sampled from a diatomaceous shale formation in Horonobe, Japan	Deep-sea sediment sourced off the coast of Taiwan	Sediment under nuclear power plant
**Geochemical parameters**	4602m depth (0.006154° N, 81.031163° E)	Gray Lowland Soil	Methanogenic, found in high ammonia and high salt biogas-synthesizing digestors	Hydrogenotrophic methanogenic	Hydrogenotrophic methanogenic	Hydrogenotrophic methanogenic	Hydrogenotrophic, methanogenic

H Environment-related pairs FCGRs

Our study employed a three-layer method to identify microbial pairs that share similar genomic signatures despite originating from maximally divergent domains. The final set includes 15 bacterium-archaeaon pairs. The FCGRs of these pairs are shown in Figures Figure S12 and Figure S13. Figure Figure S12 illustrates Groups 1 to 3, all of which inhabit extreme environments, while Figure Figure S13 shows Groups 4 and 5, which mainly consist of mesophiles.

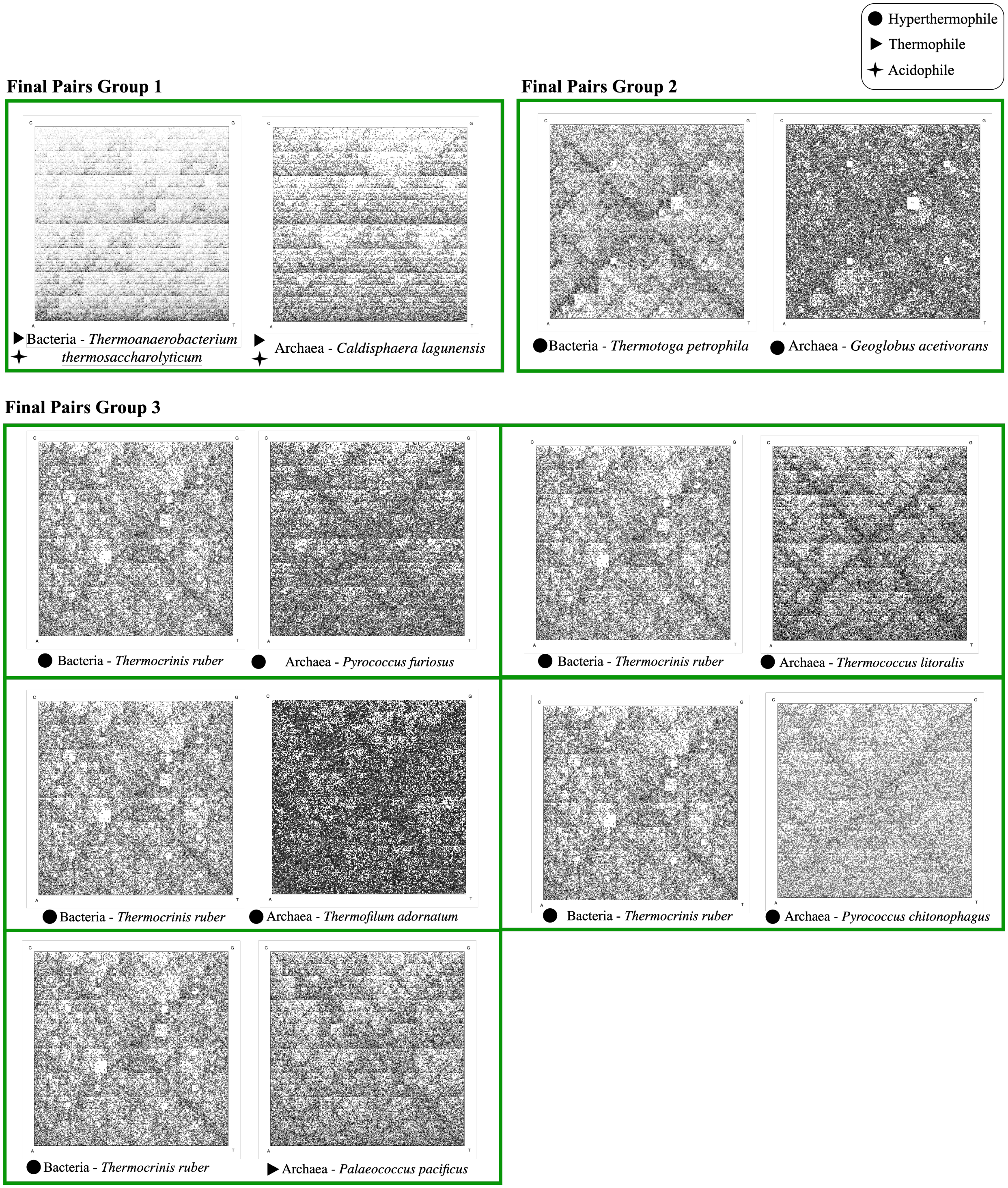

Figure S12: The FCGRs of all pairs in Groups 1, 2, and 3. We used $k = 8$ to generate the FCGRs.

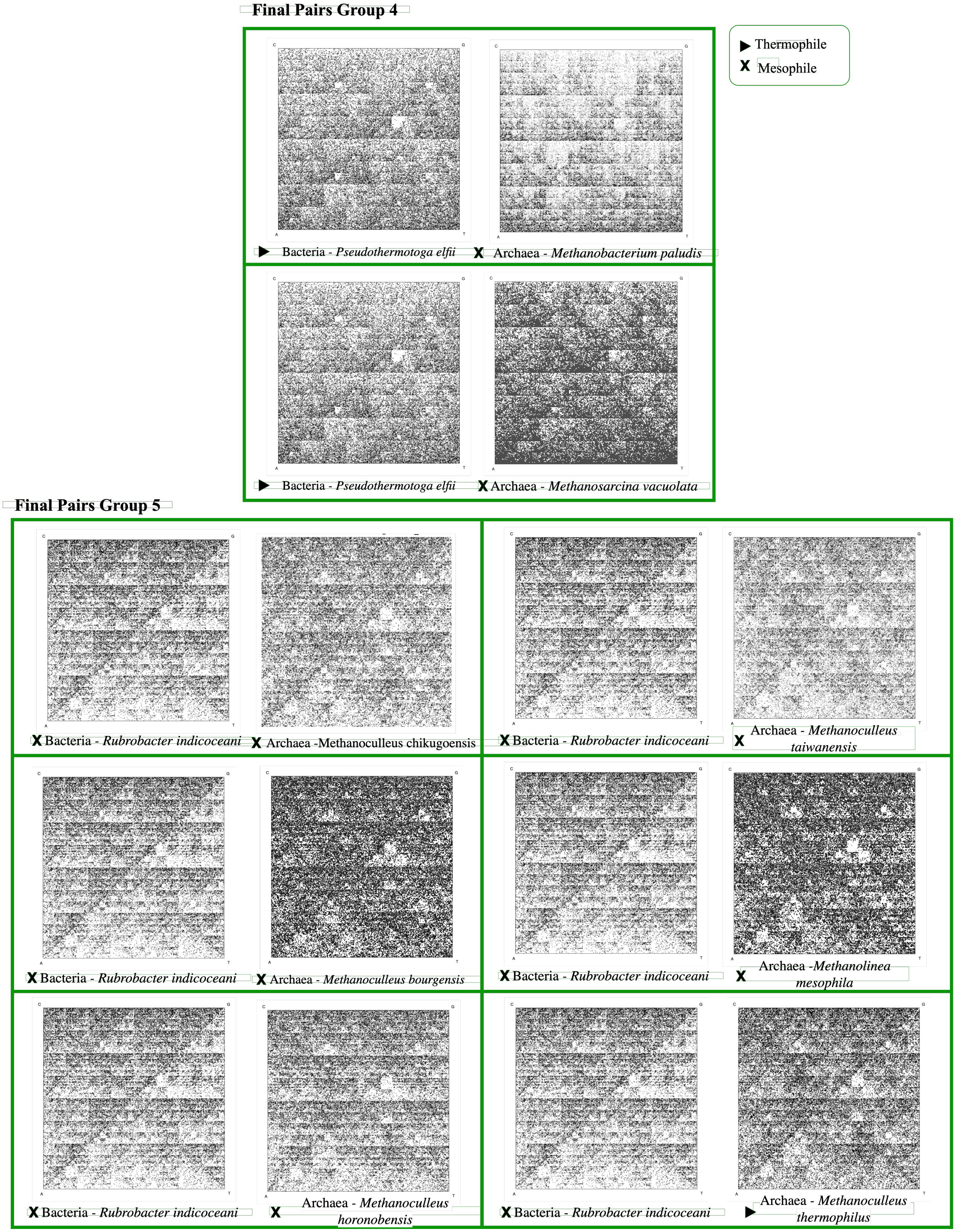

Figure S13: The FCGRs of all pairs in environment-related pairs Group 4 and 5. We used $k = 8$ to generate the FCGRs.

I Analysis of 3-mer profiles of confirmed bacterium-archaeon pairs

To investigate potential biases in 3-mer usage related to environmental adaptation, we performed a detailed analysis of the 3-mer profiles for the genome proxies of the organisms in the "confirmed bacterium-archaeon pairs" groups. The focus was on $k = 3$, due to its biological significance, as codons form a subset of the set of 3-mers. Following the four-step analysis, this section examines how the 3-mer frequencies reflect environmental adaptations across taxonomically divergent microbes. The results of this comprehensive analysis are discussed separately below, for each of the five "confirmed bacterium-archaeon pairs" groups.

Confirmed Pairs Group 1 (Compelling). This group consists of two thermophilic acidophiles: *T. thermosaccharolyticum*, (bacterium), and *C. lagunensis* (archaeon). As detailed below, all four steps of the 3-mer analysis concurred in showing remarkable genomic signature similarities between the two organisms.
3-mer deviation analysis: As illustrated in Figure S14, both organisms exhibited similar patterns of 3-mer over- and under-representation, relative to the dataset average.
Environment-relevant 3-mers identification: Figure S14 shows that the two species had 10 environment-relevant 3-mers in common with similar over- and under-representation, out of a total of 15 environment-relevant 3-mers identified for each organism.
Correlation assessment: As shown in Figure S15, the similarity in 3-mer profiles was quantitatively confirmed by a Spearman's rank correlation coefficient of 0.96 with p-value $< 10^{-5}$ between the 3-mer counts of the two organisms. This indicates a statistically significant correlation in the 3-mer composition of the two genomic signatures.
Comparison with literature: As shown in Table S16, our findings in the 3-mer bias patterns in *C. lagunensis* showed nearly complete agreement with previously reported findings in the biology literature, with 12 out of 15 3-mers showing agreement with the literature. Similarly, our findings for *T. thermosaccharolyticum* showed strong agreement with published codon usage and amino acid abundance patterns, with 12 of 15 environment-relevant 3-mers agreeing with established findings. The comparison between the two species also revealed 9 3-mers with similar frequencies in both genomes, which also aligned with published findings in the literature.

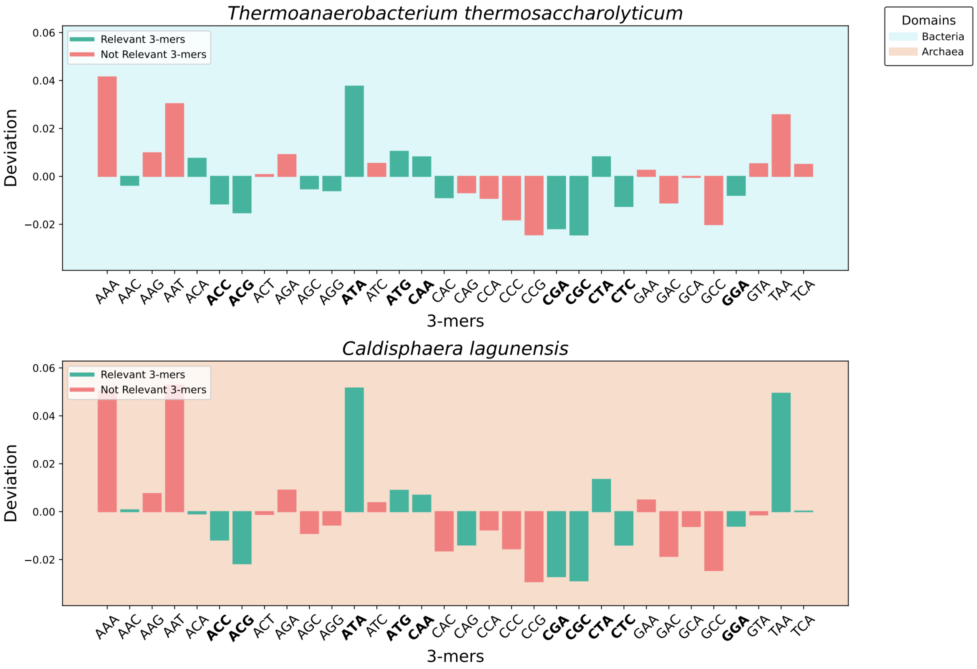

Figure S14: 3-mer usage bias analysis of the Confirmed Pairs Group 1, which includes two species: one bacterium, *Thermoanaerobacterium thermosaccharolyticum*, and one archaeon, *Caldisphaera lagunensis*. The top panel displays the deviation of each 3-mer in *T. thermosaccharolyticum* relative to its average frequency within both *Temperature Dataset* and *pH Dataset*, while the bottom panel shows the same analysis for *C. lagunensis*. Green bars indicate 3-mers identified as relevant to the environment-type classification for that species, whereas red bars represent 3-mers that did not influence that classification. Only canonical 3-mers are considered in this analysis. Shared environment-relevant 3-mers with similar over- and under-representation are indicated in boldface.

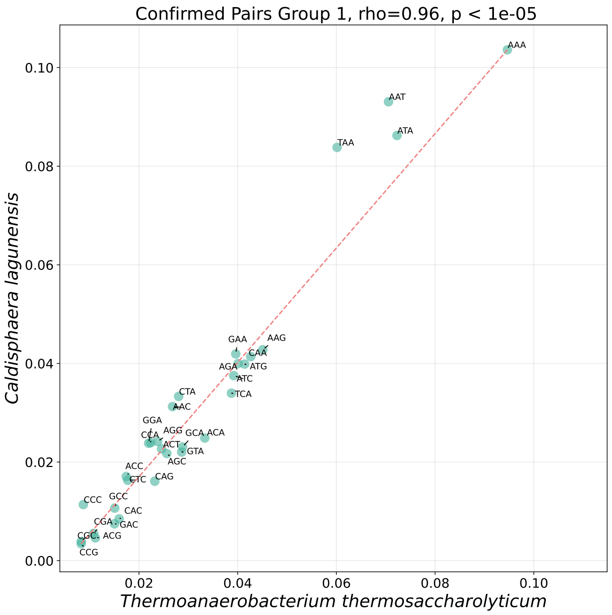

Figure S15: Correlation analysis of 3-mer usage between the bacterium *Thermoanaerobacterium thermosaccharolyticum* and the archaeon *Caldisphaera lagunensis*, pair-members from the Confirmed Pairs Group 1. Each point represents a specific 3-mer, with its normalized frequency in *T. thermosaccharolyticum* plotted on the x-axis and its frequency in *C. lagunensis* on the y-axis. The analysis reveals a strong positive Spearman's rank correlation coefficient, $\rho = 0.96$ with p-value $< 10^{-5}$, between the 3-mer usage patterns of the two organisms.

Table S16: Over- and under-representation of the relevant 3-mers, found by our method to be collectively associated with genomic signatures of extremophiles found in Group 1. The symbol ↑ (↓) indicates over-representation (under-representation) of a 3-mer/codon. Matched arrows, e.g., (↑, ↑) indicate that both our method and the literature agree in their finding. Mismatched arrows indicate disagreement. In cases where the letter "X" is found in place of a secondary arrow indicates a lack of supporting literature. The 3-mers with complete matching patterns between the bacteria and the archaea in this group are highlighted in bold.

**Domain**	Bacteria	Archaea
**Species**	*Thermoanaerobacterium thermosaccharolyticum*	*Caldisphaera lagunensis*
**Temperature Label**	Thermophiles	Thermophiles
**pH Label**	Acidophiles	Acidophiles
**Corresponding Amino Acid**	**Representation of 3-mers**	
Arg	AGG (↓, ↓)<br>**CGC (↓, ↓)**<br>**CGA (↓, ↓)**	**CGA (↓, ↓)**<br>**CGC (↓, ↓)**
Asn	AAC (↓, ↑)	AAC (↑, ↑)
Gln	**CAA (↑, ↑)**	**CAA (↑, ↑)**<br>CAG (↓, ↓)
Gly	GGA (↓, ↑)	GGA (↓, ↓)
His	CAC (↓, ↓)	-
Ile	**ATA (↑, ↑)**	**ATA (↑, ↑)**
Leu	**CTA (↑, ↑)**<br>**CTC (↓, ↓)**	**CTA (↑, ↑)**<br>**CTC (↓, ↓)**
Met	ATG (↑, X)	ATG (↑, X)
Ser	AGC (↓, ↓)	TCA (↑, ↓)
STOP	-	TAA (↑, X)
Thr	**ACA (↑, ↑)**<br>**ACC (↓, ↓)**<br>**ACG (↓, ↓)**	**ACA (↑, ↑)**<br>**ACC (↓, ↓)**<br>**ACG (↓, ↓)**

Confirmed Pairs Group 2 (strong). This group consists of one pair of hyperthermophiles, *T. petrophila* (bacterium), and *G. acetivorans* (archaeon). For this group, three out of the four steps of the analysis showed remarkable genomic signature similarities between the two organisms, with the fourth indicating a moderate level of agreement with biological literature.
3-mer deviation analysis: As illustrated in Figure S16, similar to Group 1, both organisms displayed remarkable convergence in their patterns of over- or under-representation relative to the dataset average.
Environment-relevant 3-mers identification: Feature importance analysis revealed that the two organisms had in common eight environment-relevant 3-mers with similar over- and under-representation patterns.

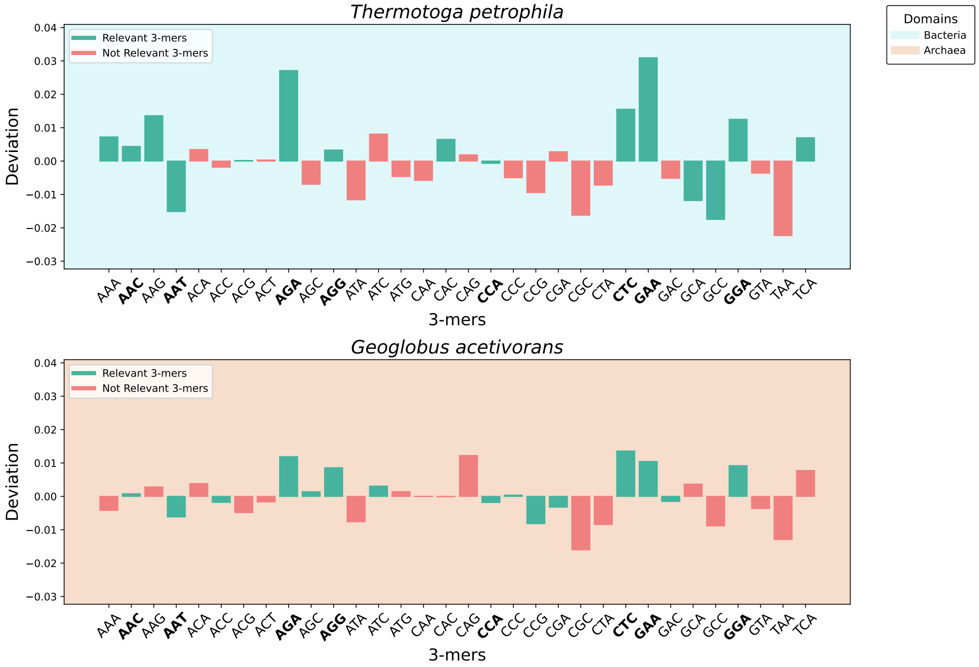

Figure S16: 3-mer usage bias analysis of the Confirmed Pairs Group 2, which includes two species: one bacterium, *T. petrophila*, and one archaeon, *G. acetivorans*. The top panel displays the deviation of each 3-mer in *T. petrophila* relative to its average frequency within *Temperature Dataset*, while the bottom panel shows the same analysis for *G. acetivorans*. Green bars indicate 3-mers identified as relevant to the environment-type classification for that species, whereas red bars represent 3-mers that did not influence that classification. Only canonical 3-mers are considered in this analysis. Shared environment-relevant 3-mers with similar over- and under-representation patterns are indicated in boldface.

Correlation assessment: As shown in Figure S17, the similarity in 3-mer frequency profiles was quantitatively confirmed by a Spearman's rank correlation coefficient of 0.81 with p-value $< 10^{-5}$ between the 3-mer counts of the two organisms.
Comparison with literature: As shown in Table S17, our findings revealed that for *T. petrophila*, seven out of 15 3-mers were either over- or under-represented in a pattern consistent with

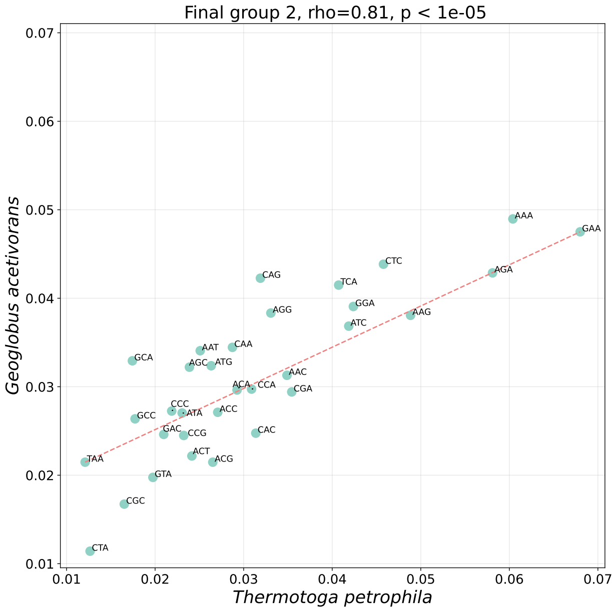

Figure S17: Correlation analysis of 3-mer usage between the bacterium *T. petrophila* and the archaeon *G. acetivorans*, pair, members from the Confirmed Pairs Group 2. Each point represents a specific 3-mer, with its normalized frequency in *T. petrophila* plotted on the x-axis and its frequency in *G. acetivorans* on the y-axis. The analysis reveals a strong positive Spearman's rank correlation coefficient, $\rho = 0.81$ with p-value $< 10^{-5}$, between the 3-mer usage patterns of the two organisms.

previously reported patterns in the literature. Similarly, for *G. acetivorans*, eight out of 15 3-mers showed agreement with known bias trends in the literature. Overall, there was a moderate level of agreement between the 3-mer bias patterns identified by our method and the codon usage and amino acid abundance biases reported in other studies. Additionally, the comparison between the two species revealed four 3-mers with similar frequencies across both genomes, further supporting observations previously documented in the literature.

Table S17: Over- and under-representation of the relevant 3-mers, found by our method to be collectively associated with genomic signatures of extremophiles found in the Confirmed Pairs Group 2. The symbol ↑ (↓) indicates over-representation (under-representation) of a 3-mer/codon. Matched arrows, e.g., (↑, ↑) indicate that both our method and literature agree in their finding. Mismatched arrows indicate disagreement. In cases where the letter "X" is found in place of a secondary arrow indicates a lack of supporting literature. The 3-mers with complete matching patterns between the bacteria and the archaea in this group are highlighted in bold.

**Domain**	Bacteria	Archaea
**Species**	*Thermotoga petrophila*	*Geoglobus acetivorans*
**Temperature Label**	Hyperthermophiles	Hyperthermophiles
**Corresponding Amino Acid**	**Representation of 3-mers**	
Ala	GCA (↓, X)<br>GCC (↓, X)	-
Arg	**AGA (↑, ↑)**<br>**AGG (↑, ↑)**	**AGA (↑, ↑)**<br>**AGG (↑, ↑)**<br>CGA (↓, ↑)
Asn	AAC (↑, ↓)<br>**AAT (↓, ↓)**	AAC (↑, ↓)<br>**AAT (↓, ↓)**
Asp	-	GAC (↓, X)
Glu	**GAA (↑, ↑)**	**GAA (↑, ↑)**
Gly	GGA (↑, X)	GGA (↑, X)
His	CAC (↑, ↓)	-
Ile	-	ATC (↑, ↑)
Leu	CTC (↑, ↑)	CTC (↑, X)
Lys	AAA (↑, ↑)<br>AAG (↑, ↑)	-
Pro	CCA (↑, ↓)	CCA (↓, ↓)<br>CCG (↓, ↓)<br>CCC (↑, ↓)
Ser	TCA (↑, ↓)	AGC (↑, ↓)
Thr	ACG (↑, ↓)	ACC (↓, ↓)

Confirmed Pairs Group 3 (very strong). This group includes six hyperthermophiles: one bacterium, *T. ruber*, and five archaea: *P. furiosus*, *T. litoralis*, *T. adornatum*, *P. pacificus*, and *T. chitonophagus*. All four steps of the analysis consistently showed remarkable genomic signature similarities between the bacterium and at least one of the archaea.
3-mer deviation analysis: As shown in Figure S18, the 3-mer frequency profiles for all six organisms in this group exhibited similar patterns of over- and under-representation from the dataset average, with notable similarities between the bacterium and the five archaea.
Environment-relevant 3-mers identification: Feature importance analysis revealed a significant overlap in environment-relevant 3-mers across these taxonomically diverse organisms, which is shown in Figure S18.

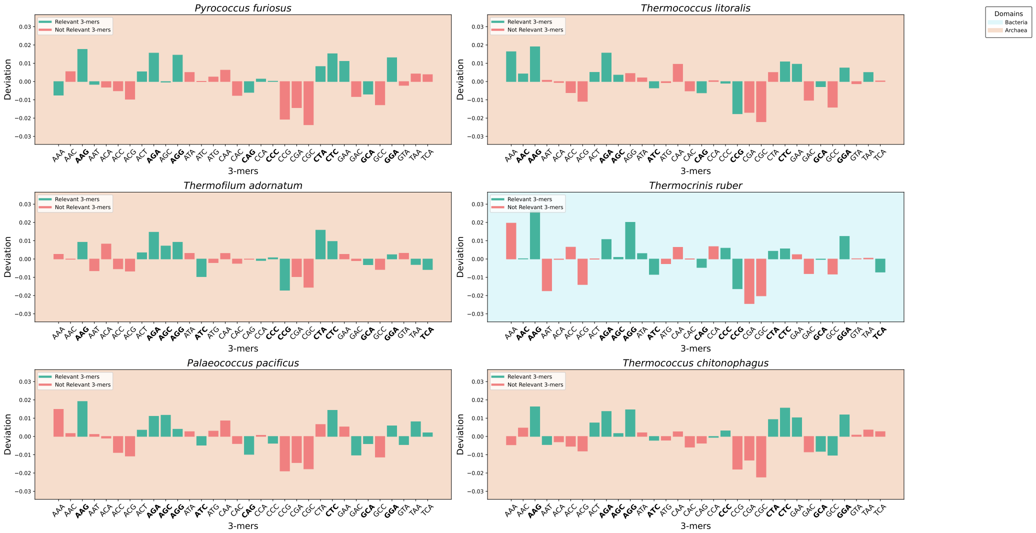

Figure S18: 3-mer usage bias analysis of the Confirmed Pairs Group 3. The blue panel shows the bacterium, and the orange panels show the archaea. The bars show the deviation of each 3-mer relative to the average of that 3-mer in the *Temperature dataset*. Green bars indicate 3-mers identified as relevant to the environment-type classification for that species, whereas red bars represent 3-mers that did not influence that classification. Only canonical 3-mers are considered in this analysis. Shared environment-relevant 3-mers with similar over- and under-representation are indicated in boldface.

Correlation assessment: As shown in Figure S19, the similarity in 3-mer profiles was confirmed by Spearman's rank correlation coefficients (mean correlation 0.79, range $0.76 - 0.81$, all with p-value $< 10^{-5}$) between the 3-mer counts of the bacterium and each of the five archaea.

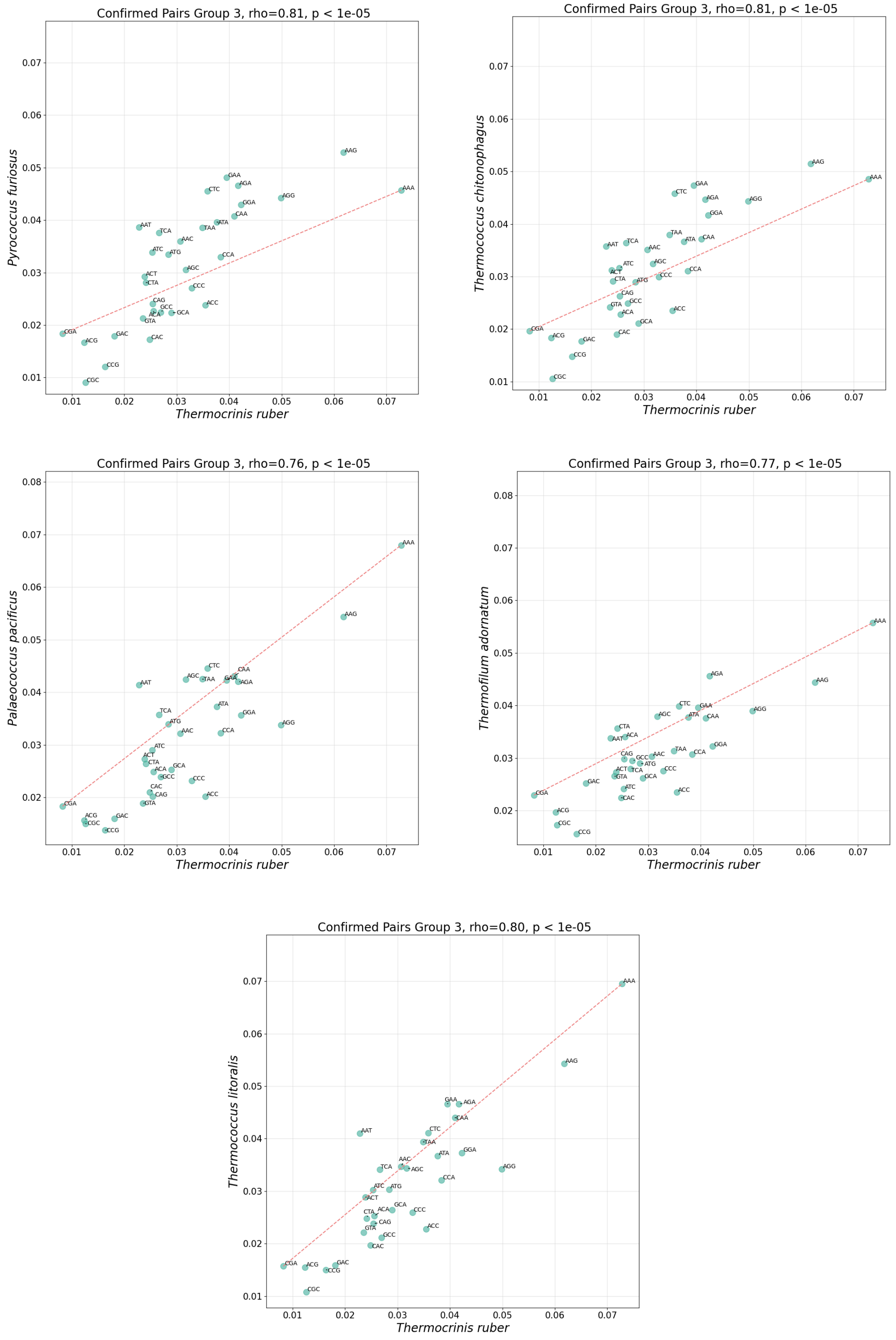

Figure S19: Correlation analysis of 3-mer usage between the pairs in Confirmed Pairs Group 3. Each point represents a specific 3-mer, with its normalized frequency in *T. ruber* plotted on the x-axis and its frequency in the five archaea on the y-axis. The analysis reveals a strong positive Spearman's rank correlation coefficient, with p-value $< 10^{-5}$ for all pairs.

Comparison with literature: As shown in Table S18, there was a moderate agreement between our findings and previously reported over- and under-representation patterns in the biology literature.

Table S18: Over- and under-representation of the relevant 3-mers, found by our method to be collectively associated with genomic signatures of extremophiles found in the Confirmed Pairs Group 3. The symbol ↑ (↓) indicates over-representation (under-representation) of a 3-mer/codon. Matched arrows, e.g., (↑, ↑ ref) indicate that both our method and literature agree in their finding. Mismatched arrows indicate disagreement. In cases where the letter "X" is found in place of a secondary arrow indicates a lack of supporting literature. The 3-mers with complete matching patterns are highlighted in bold.

**Domain**	Bacteria	Archaea				
**Species**	*Thermocrinis ruber*	*Pyrococcus furiosus*	*Thermofilum adornatum*	*Palaeococcus pacificus*	*Thermococcus chitonophagus*	*Thermococcus litoralis*
**Temperature Label**	Hyper-thermophiles	Hyper-thermophiles	Hyper-thermophiles	Hyper-thermophiles	Hyper-thermophiles	Hyper-thermophiles
**Corresponding Amino Acid**	**Representation of 3-mers**					
Ala	GCA (↓, *X*)	GCA (↓, *X*)	GCA (↑, *X*)		GCA (↓, *X*)<br>GCC (↓, *X*)	GCA (↓, *X*)
Arg	**AGA (↑, ↑)**<br>**AGG (↑, ↑)**	**AGA (↑, ↑)**<br>**AGG (↑, ↑)**	**AGA (↑, ↑)**<br>**AGG (↑, ↑)**	**AGA (↑, ↑)**<br>**AGG (↑, ↑)**	**AGA (↑, ↑)**<br>**AGG (↑, ↑)**	**AGA (↑, ↑)**
Asn	AAC (↑, ↓)	AAT (↓, ↓)	AAT (↓, ↓)		AAT (↓, ↓)	AAC (↑, ↓)
Asp	GAC (↓, *X*)			GAC (↓, *X*)		GAC (↓, *X*)
Gln	**CAG (↓, ↓)**	**CAG (↓, ↓)**		**CAG (↓, ↓)**		**CAG (↓, ↓)**
Glu		GAA (↑, ↑)			GAA (↑, ↑)	GAA (↑, ↑)
Gly	GGA (↑, *X*)	GGA (↑, *X*)	GGA (↓, *X*)	GGA (↑, *X*)<br>GCA (↓, *X*)	GGA (↑, *X*)	GGA (↑, *X*)
His						
Ile	ATA (↑, ↑)<br>ATC (↓, ↑)		ATC (↓, ↑)	ATC (↓, ↑)	ATC (↓, ↑)	ATC (↓, ↑)
Leu	**CTA (↑, ↑)**	**CTA (↑, ↑)**<br>CTC (↑, ↑)	**CTA (↑, ↑)**<br>CTC (↑, ↑)	CTC (↑, ↑)	**CTA (↑, ↑)**<br>CTC (↑, ↑)	CTC (↑, ↑)
Lys	**AAG (↑, ↑)**	**AAG (↑, ↑)**<br>AAA (↓, ↑)	**AAG (↑, ↑)**	**AAG (↑, ↑)**	**AAG (↑, ↑)**	**AAG (↑, ↑)**<br>AAA (↑, ↑)
Pro	CCC (↑, ↓)<br>**CCG (↓, ↓)**	CCA (↑, ↓)	CCC (↑, ↓)<br>**CCG (↓, ↓)**		CCC (↑, ↓)<br>CCA (↓, ↓)	CCC (↓, ↓)<br>**CCG (↓, ↓)**
Ser	AGC (↑, ↓)<br>TCA (↓, ↓)	AGC (↓, ↓)	AGC (↑, ↓)<br>TCA (↑, ↓)	AGC (↑, ↓)<br>TCA (↑, ↓)	AGC (↑, ↓)	
STOP		TAA (↑, *X*)	TAA (↑, *X*)			
Thr		ACT (↑, ↓)	ACT (↑, ↓)	ACT (↑, ↓)	ACT (↑, ↓)<br>ACC (↓, ↓)	ACT (↑, ↓)
Val				GTA (↓, ↑)		

Confirmed Pairs Group 4 (strong). This group includes one thermophilic bacterium, *P. elfii* and two mesophilic archaea, *M. paludis* and *M. vacuolata*. Three of four steps of the analysis consistently showed genomic signature similarities between the bacteria and at least one of the archaea, with the fourth indicating a moderate level of agreement with existing biological literature.
3-mer deviation analysis: As illustrated Figure S20, the 3-mer frequency profiles for all three organisms in this exhibited similar patterns of deviation from the dataset average.
Environment-relevant 3-mers identification: Feature importance analysis revealed an overlap in environment-relevant 3-mers across these organisms, which is shown in Figure S20.

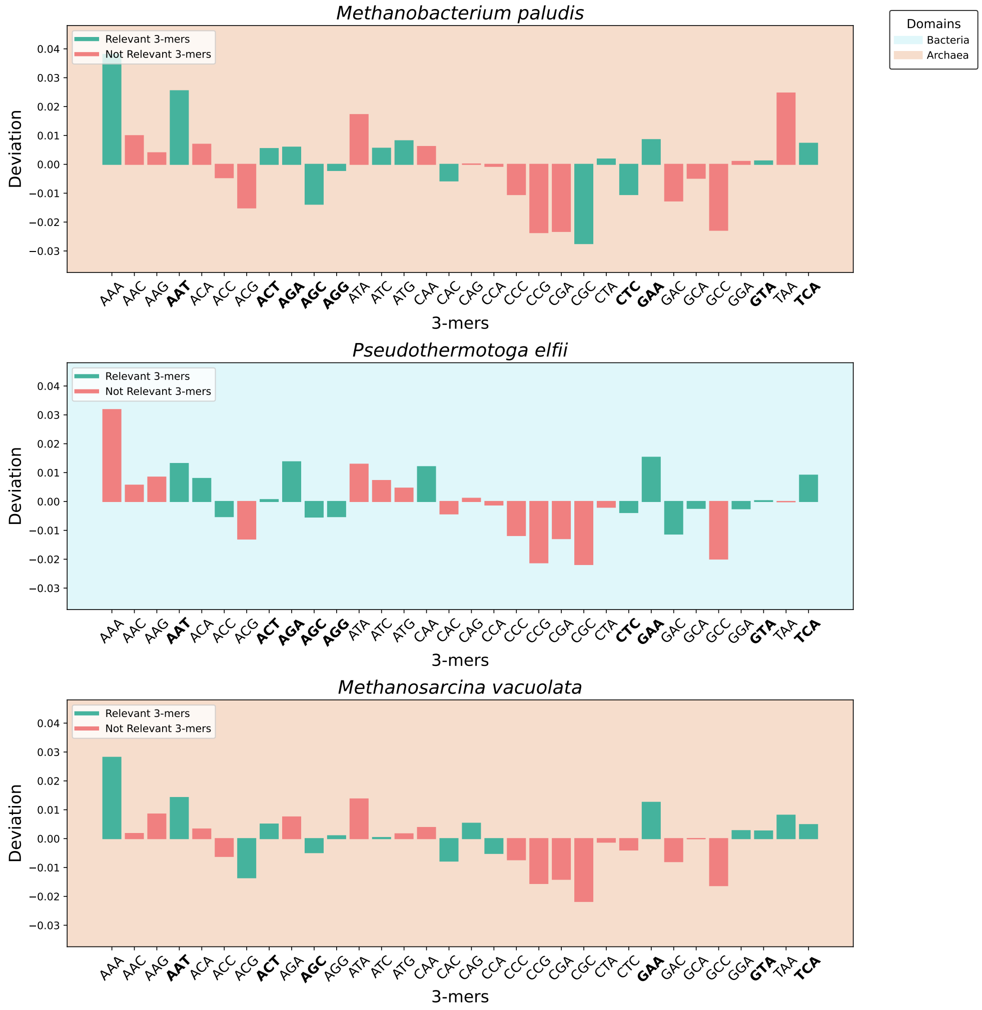

Figure S20: 3-mer usage bias analysis of the Confirmed Pairs 4. The blue panel shows the bacterium, and the orange panels show the archaea. The bars show the deviation of each 3-mer relative to the average of that 3-mer in the *Temperature dataset*. Green bars indicate 3-mers identified as relevant to the environment-type classification for that species, whereas red bars represent 3-mers that did not influence that classification. Only canonical 3-mers are considered in this analysis. Shared environment-relevant 3-mers with similar over- and under-representation are indicated in boldface.

Correlation assessment: As shown in Figure S21, the similarity in 3-mer profiles was quantitatively confirmed by high Spearman's rank correlation coefficients (mean correlation = 0.945, all with p-value $< 10^{-5}$) between the 3-mer counts of the bacterium and each of the two archaea.

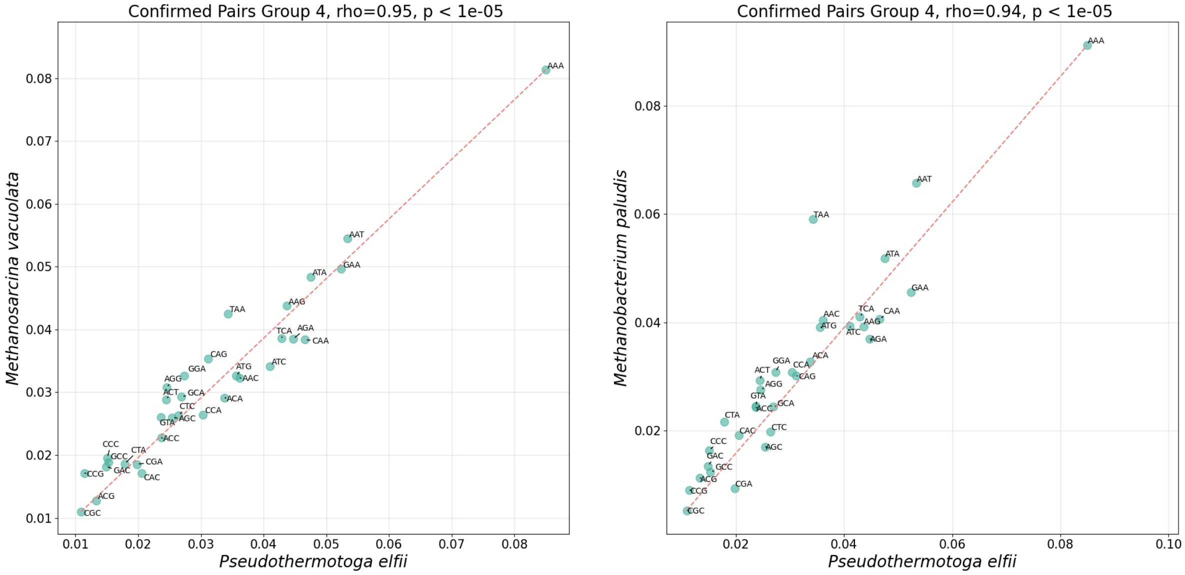

Figure S21: Correlation analysis of 3-mer usage between the pairs from the Confirmed Pairs 4. Each point represents a specific 3-mer, with its normalized frequency in *P. elfi* plotted on the x-axis and its frequency in the archaea on the y-axis. The analysis reveals a strong positive Spearman's rank correlation coefficient, with p-value $< 10^{-5}$ for all pairs.

Comparison with literature: As shown in Table S19, compared with s 1, 2 and 3, less agreement was found between the 3-mer biases identified by our method and biology literature findings.

Table S19: Over- and under-representation of the relevant 3-mers, found by our method to be collectively associated with genomic signatures of extremophiles found in Confirmed Pairs Group 4. The symbol ↑ (↓) indicates over-representation (under-representation) of a 3-mer/codon. Matched arrows, e.g., (↑, ↑) indicate that both our method and literature agree in their finding. Mismatched arrows indicate disagreement. In cases where the letter "X" is found in place of a secondary arrow indicates a lack of supporting literature. The 3-mers with complete matching patterns across species are highlighted in bold.

**Domain**	Bacteria	Archaea	
**Species**	*Pseudothermotoga elfii*	*Methanobacterium paludis*	*Methanosarcina vacuolata*
**Temperature Label**	Thermophiles	Mesophiles	Mesophiles
**Corresponding Amino Acid**	**Representation of 3-mers**		
Ala	GCA (↓, X)	-	-
Arg	AGG (↓,↑)	AGA (↑,↓)<br>AGG (↓,↓)<br>CGC (↓,↑)	AGG (↑,↓)
Asn	AAT (↑, X)	AAT (↑,↑)	AAT (↑,↑)
Asp	GAC (↓, X)	-	-
Gln	CAA (↑,↓)	-	CAG (↑,↑)
Glu	GAA (↑,↑)	GAA (↑, X)	GAA (↑, X)
Gly	GGA (↓,↑)	-	GGA (↑,↓)
His	-	CAC (↓,↑)	CAC (↓,↑)
Ile	-	ATC (↑,↓)	ATC (↑,↓)
Leu	CTC (↓,↑)	CTC (↓, X)	-
Lys	-	AAA (↑, X)<br>CTA (↑,↓)	AAA (↑, X)
Met	-	ATG (↑,↑)	-
Pro	-	-	CCA (↓,↓)
Ser	**AGC (↓,↓)**<br>TCA (↑,↓)	**AGC (↓,↓)**<br>TCA (↑,↑)	**AGC (↓,↓)**<br>TCA (↑,↑)
STOP	-	-	TAA (↑,↑)
Thr	ACA (↑, -)<br>ACC (↓,↓)<br>ACT (↓,↓)	ACT (↑,↓)	ACT (↑, X)<br>ACG (↓,↓)
Val	GTA (↑,↑)	GTA (↑, X)	GTA (↑, X)

Confirmed Pairs 5 (strong). This group includes one mesophilic bacterium, *R. indicoceani*, five mesophilic archaea, *M. chikugoensis*, *M. bourgensis*, *M. mesophila*, *M. horonobensis*, and *M. taiwanensis*, and one thermophilic archaeon *M. thermophilus*. Three out of four steps of the analysis revealed remarkable genomic signature similarities between the bacteria and at least one of the archaea, but less agreement was found with biological literature.
3-mer deviation analysis: As shown in Figure S22, the 3-mer frequency profiles for all three organisms in this group showed similar patterns of deviation from the dataset average.
Environment-relevant 3-mer identification: Feature importance analysis revealed an overlap in environment-relevant 3-mers across these organisms, which is shown in Figure S22.

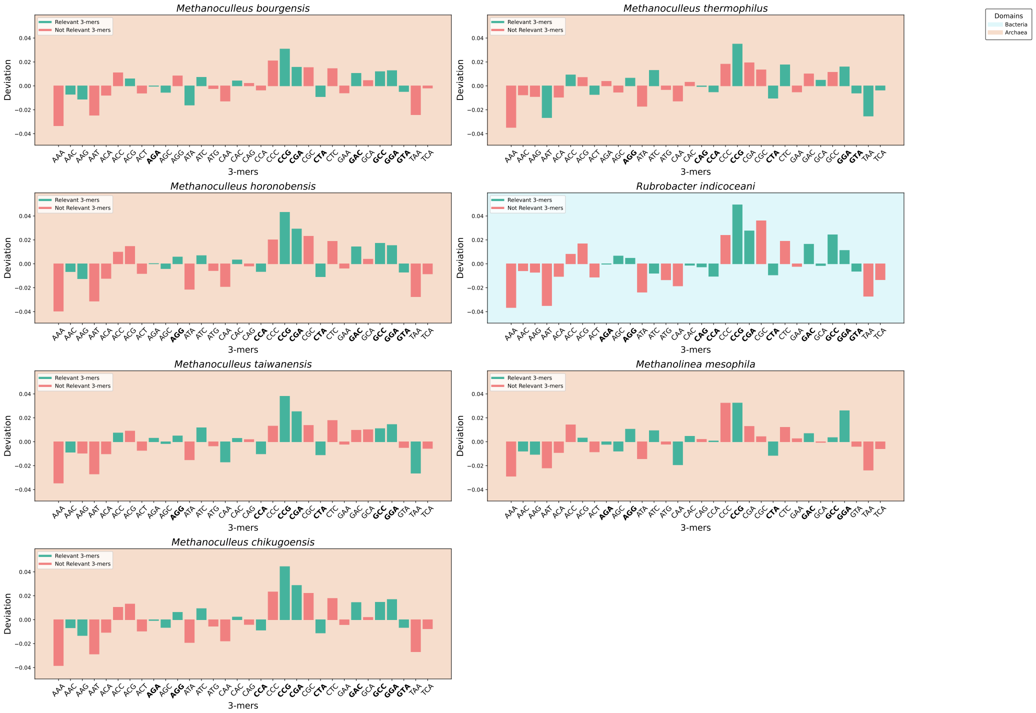

Figure S22: 3-mer usage bias analysis of the Confirmed Pairs Group 5. The blue panel shows the bacterium, and the orange panels show the archaea. The bars show the deviation of each 3-mer relative to the average of that 3-mer in the *Temperature dataset*. Green bars indicate 3-mers identified as relevant to the environment-type classification for that species, whereas red bars represent 3-mers that did not influence that classification. Only canonical 3-mers are considered in this analysis. Shared environment-relevant 3-mers with similar over- and under-representation are indicated in boldface.

Correlation assessment: As shown in Figure S23, the similarity in 3-mer profiles was quantitatively confirmed by high Spearman's rank correlation coefficients (all with p-value $< 10^{-5}$) between the 3-mer counts of the bacterium and each of the archaea.

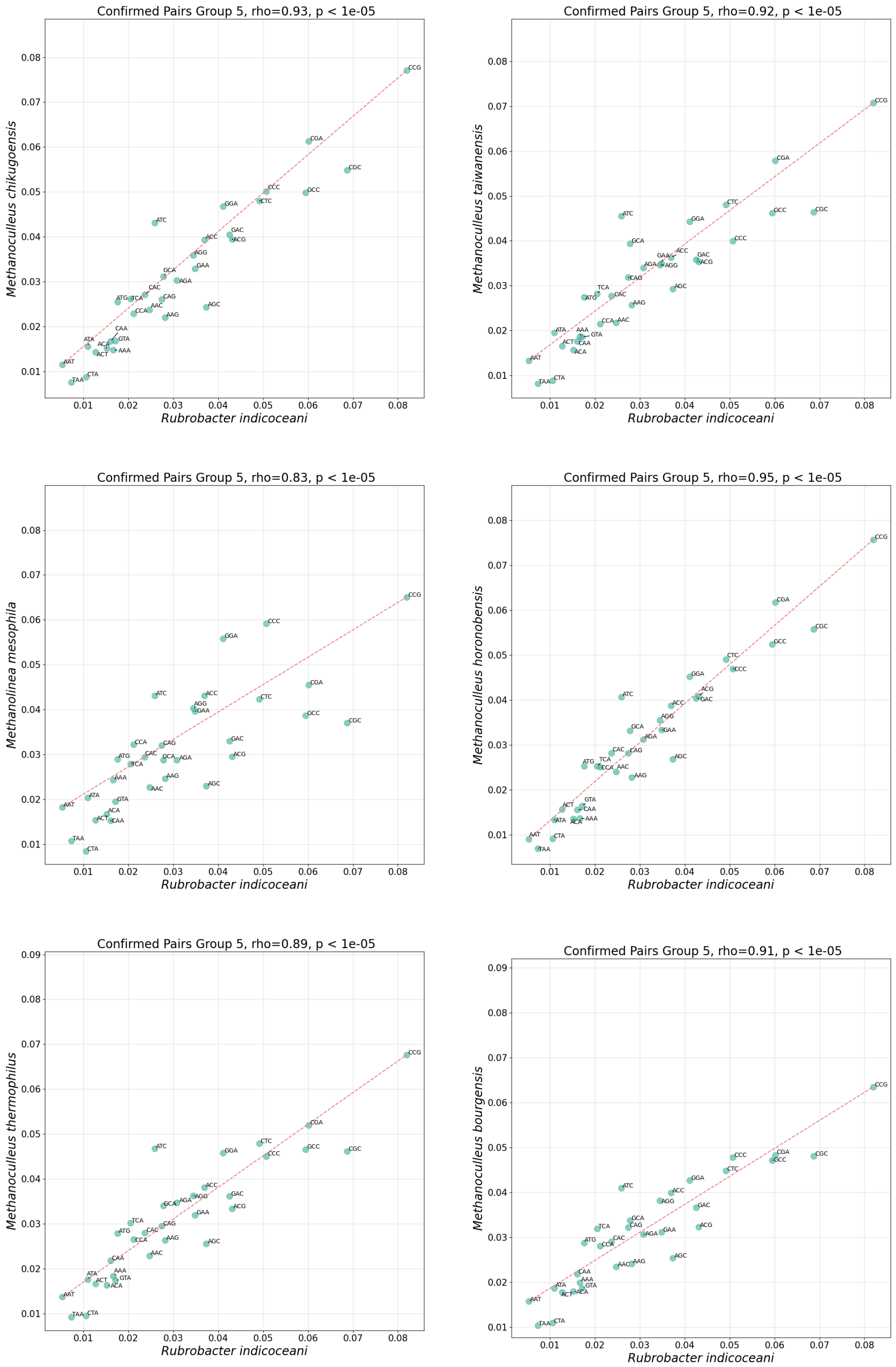

Figure S23: Correlation analysis of 3-mer usage between the pairs in Confirmed Pairs Group 5. Each point represents a specific 3-mer, with its normalized frequency in *R. indicoceani* plotted on the x-axis and its frequency in the six archaea on the y-axis. The analysis reveals a strong positive Spearman's rank correlation coefficient, with p-value $< 10^{-5}$ for all pairs.

Comparison with literature: As shown in Table S20, similar to Group 4, less agreement was found between 3-mer biases identified by our method and biology literature findings.

Table S20: Over- and under-representation of the relevant 3-mers, found by our method to be collectively associated with genomic signatures found in Confirmed Pairs Group 5. The symbol ↑ (↓) indicates over-representation (under-representation) of a 3-mer/codon. Matched arrows, e.g., (↑, ↑) indicate that both our method and literature agree in their finding. Mismatched arrows indicate disagreement. In cases where the letter "X" is found in place of a secondary arrow indicates a lack of supporting literature. The 3-mers with complete matching patterns across species are highlighted in bold.

**Domain**	Bacteria	Archaea					
**Species**	*Rubrobacter indicoceani*	*M. chikugoensis*	*M. bourgensis*	*M. thermophilus*	*M. horonobensis*	*M. taiwanensis*	*Methanolinea mesophila*
**Temperature Label**	Mesophiles	Mesophiles	Mesophiles	Thermophiles	Mesophiles	Mesophiles	Mesophiles
**Corresponding Amino Acid**	**Representation of 3-mers**						
Ala	AGA (↑, ↓)<br>GCA (↓,↓)<br>GCC (↑, X)	GCC (↑, X)	GCC (↑, X)	GCA (↑, X)	GCC (↑, X)	GCC (↑, X)	GCC (↑, X)
Arg	AGG (↑,↓)<br>**CGA (↑, ↑)**	AGA (↓,↓)<br>AGG (↑, ↓)<br>**CGA (↑, ↑)**	AGA (↓,↓)<br>**CGA (↑, ↑)**	AGG (↑, ↑)	AGG (↑,↓)<br>**CGA (↑, ↑)**	AGA (↓,↓)<br>AGG (↑, ↓)<br>**CGA (↑, ↑)**	AGA (↓,↓)
Asn	-	AAC (↓, ↑)	AAC (↓, ↑)	AAT (↓, X)	AAC (↓, ↑)	AAC (↓, ↑)	AAC (↓, ↑)
Asp	GAC (↑, X)	-	GAC (↑, X)	-	GAC (↑, X)	-	GAC (↑, X)
Gln	CAG (↓, ↑)	-	-	CAG (↓, ↑)	-	-	CAA (↓, X)
Glu	-	-	-	-	-	-	-
Gly	GGA (↑,↓)	GGA (↑,↓)	GGA (↑,↓)	GGA (↑, ↑)	GGA (↑,↓)	GGA (↑,↓)	GGA (↑,↓)
His	CAC (↓, ↑)	CAC (↑, ↑)	CAC (↑, ↑)	-	CAC (↑, ↑)	CAC (↑, ↑)	CAC (↑, ↑)
Ile	ATC (↓,↓)	ATA (↓, ↓)<br>ATC (↑, ↓)	ATA (↓, ↓)<br>ATC (↑, ↓)	ATC (↑, ↑)	ATC (↑,↓)	ATC (↑,↓)	ATC (↑,↓)
Leu	**CTA (↓,↓)**	**CTA (↓,↓)**	**CTA (↓,↓)**	CTA (↓, ↑)<br>CTC (↑, ↑)	**CTA (↓,↓)**	**CTA (↓,↓)**	**CTA (↓,↓)**
Lys	-	AAG (↓, X)	AAG (↓, X)	-	AAG (↓, X)	AAG (↓, X)	AAG (↓, X)
Met	-	-	-	-	-	-	-
Pro	**CCA (↓,↓)**<br>CCG (↑,↓)	**CCA (↓,↓)**<br>CCG (↑,↓)	CCG (↑,↓)	CCA (↓, ↑)<br>CCG (↑, X)	**CCA (↓,↓)**<br>CCG (↑,↓)	**CCA (↓,↓)**<br>CCG (↑,↓)	CCA (↑,↓)<br>CCG (↑,↓)
Ser	AGC (↑,↓)	AGC (↓,↓)	AGC (↓,↓)	TCA (↓,↓)	AGC (↓,↓)	AGC (↓,↓)	AGC (↓,↓)
STOP	-	-	-	TAA (↓, X)	-	TAA (↓, ↑)	TAA (↓, ↑)
Thr	-	-	ACG (↑,↓)	ACC (↑,↓)<br>ACT (↓,↓)	-	ACC (↑, X)<br>ACG (↑,↓)	-
Val	GTA (↓, X)	GTA (↓, X)	-	GTA (↓, ↑)	GTA (↓, X)	-	-

Table S21 shows the references to the literature used for this study.

J Co-occurrences details

This section provides details of the geographical co-occurrences of Groups 1 to 4, in Table S22, Table S23, Table S24, Table S25, respectively. No co-occurrence was found for Group 5.

Table S21: Codon usage biases across extremophile groups as characterized in the literature. The table summarizes the various codon usage biases, considering related amino acid abundance patterns associated with prokaryotes across pH and temperature-adapted extremophilic categories. Each observation is followed by [ref], linking to the paper providing the rationale for this pattern.

**Codon Usage Biases**	**Acidophiles**	**Alkaliphiles**	**Psychrophiles**	**Mesophiles**	**Thermophiles**	**Hyperthermophiles**
**Increased**	CAA [29]	AAC [29]	GCA [30]	ATG [31]	AGG [32]	AGG [32]
	GGA[29]	AAT [29]	CAA [33]	CAA [29]	AGA [32]	AGA [32]
	CCA [29]	GAC [29]	GAA [34]	AAT [29]	GGA [35]	CTC [32]
	CGC [29]	ATG [30]	CAG [29]	CTC [32]	CTA [32]	
	AGC [36]	CGC [37]	CCA [38]	GTA [35]		
	TCA [36]	TAT [37]	CCC [38]			
**Decreased**	AGG [29]	CAA [29]	AGG [39]	ATC [40]	ACT [38]	CCT [38]
	CTC [29]	GAG [29]	CAC [39]	ACG [37]	ACG [38]	CCC [38]
	TCA [29]		CTC [34]	TCC [37]		CCA [38]
	AAA[30]	ACA [37]			CCG [38]	
	CCA [37]		GGC [37]			
	AGT [37]			TCG [37]		

Table S22: Description of co-occurrence of species found in Group 1. The following table describes the location data for each of the co-occurrences between the two species in Group 1, including the species name for the mapped 16s rRNA reads, sample IDs for the relevant Microbe Atlas Project entry, coordinate information (Latitude, Longitude), and notable environmental observations.

**Attribute**	**Description**
Mapped Read 1 ID	*Thermoanaerobacterium thermosaccharolyticum*
Mapped Read 2 ID	*Caldisphaera lagunensis*
Sample ID	SRS5070100
Environment Descriptor	Washburn Hot Springs [41]
Coordinates	44.376° N, 110.69° W
Notable Environmental Observations	Described by McKay et al. [41] as an "ideal ancient habitat," displaying high levels of sulphate, sulphide, methane, hydrogen, carbon dioxide, and ammonium sulphate.

Table S23: Description of co-occurrence of species in Group 2. The following table describes the location data for each of the co-occurrences between the species in Group 2, including the species name for the mapped 16s rRNA reads, sample IDs for the relevant Microbe Atlas Project entry, coordinate information (Latitude, Longitude), and notable environmental observations.

**Attribute**	**Description**	
Mapped Read 1 ID	*Thermotoga petrophila*	
Mapped Read 2 ID	*Geoglobus acetivorans*	
Sample IDs	SRS5544491	SRS730398
Environment Descriptor	Brothers Volcano [42]	Juan de Fuca Ridge [43]
Coordinates	34.86208959° S, 179.0573881° E	47.753500° N, 127.763883° W
Notable Environmental Observations	Hydrothermally active submarine volcano	Deep biofilm community demonstrating iron redox chemistry

Table S24: Description of co-occurrence of species found in Group 3. The following table describes the location data for each of the co-occurrences between species found in Group 3, including the species name for the mapped 16s rRNA reads, sample IDs for the relevant Microbe Atlas Project entry, coordinate information (Latitude, Longitude), and notable environmental observations. Habitats where an occurrence was identified for a particular species are specified, displaying unique co-occurrences where location data was available (duplicates are removed).

**Sample ID**	**Environment**	**Coordinates**	**Notable Observations**	**Co-occurrences by species**		
				T. ruber	*T. litoralis*	*T. adornatum*
SRS7008575	Norris-Mammoth Corridor, YNP (Yellowstone National Park), USA	44.754° N, 110.7257° W	Hot spring water	✓		✓
SRS4347131	Culex Basin, YNP, USA	44.5775° N, 110.7896° W	Hot spring sediment	✓		✓
SRS6018221	Geyser Creek Basin, YNP, USA	44.6904° N, 110.7291° W	Hot spring sediment	✓		✓
SRS1971309	Joseph's Coat, YNP, USA	44.376° N, 110.69° W	Hot spring sediment	✓		✓
SRS2354974	Mono Lake, USA	37.97° N, 119.07° W	High salinity salt lake	✓		✓
SRS3206925	Guaymas Basin, Gulf of California	27.0114° N, 110.5931° W	Hydrothermal vent sediment	✓	✓	
ERS1370018	Mid-Cayman Rise, Cayman Trough	18°32'49.9"N, 81°43'05.2"W	Deep sea hydrothermal vent	✓	✓	
SRS5070100	Washburn Springs, YNP, USA	44.376° N, 110.69° W	Extremophilic microbial mat	✓		✓
SRS6608865	Yellowstone Lake, YNP, USA	44.5106° N, 110.3566° W	Filamentous streamer microbial communities	✓		✓
SRS5544488	Brothers Volcano, Pacific Ocean	34.8611° S, 179.0576° E	Hydrothermal vent sediment	✓		✓
ERS1372490	Manus Basin, Papua New Guinea	3°47'59.7"S, 152°06'03.1"E	Marine basaltic hydrothermal vent biome	✓	✓	

Table S25: Description of co-occurrence of species found in Group 4. The following table describes the location data for each of the co-occurrences between the species in Group 4, including the species name for the mapped 16s rRNA reads, sample IDs for the relevant Microbe Atlas Project entry, coordinate information (Latitude, Longitude), and notable environmental observations.

**Attribute**	**Description**		
Mapped Read 1 ID	*Pseudothermotoga elfii*		
Mapped Read 2 ID	*Methanobacterium paludis*		
Mapped Read 3 ID	*Methanosarcina vacuolata*		
Sample ID	DRS114146	SRS586146	SRS836040
Environment Descriptor	Bioreactor, Tokyo, Japan [44]	Shengli Oil Field, China [45]	Norman, Oklahoma, USA
Coordinates	35.7127° N, 139.7620° E	37.54° N, 118.33° E	35.2200° N, 97.4400° W
Notable Environmental Observations	Microbiome from a "thermophilic methanogenic biocathode" implying high temperature and methane conditions [44]	Anaerobic, mesophilic culture; involved in "methanogenic degradation of hydrocarbons" [45]	Landfill leachate exposed to "CH_4 and CO_2 gases" and complex soluble chemical compounds [46]

K Horizontal gene transfer experimental details

To further investigate potential genetic exchange, we performed a Basic Local Alignment Search Tool (BLAST) analysis. Pairwise alignments were conducted between the bacteria in each group (subject samples) and the archaea (query samples), using genome assemblies referenced by their RefSeq accession numbers. Using the standard megablast algorithm with default parameters, no alignments were detected between any pairs.

When applying discontinuous megablast parameters across most cluster groups, the alignments showed only 1–2% query coverage with moderate percent identity (66.51–73.14%) and numerous gaps, yet remained statistically significant ($4e^{-17}$ to $1e^{-170}$). In Cluster Group 5, alignments were not feasible due to the scaffolded nature of *M. horonobensis* and *M. taiwanensis*. Geneious annotation revealed only minimal open reading frames, limited to ubiquitous housekeeping genes. If HGT were the dominant mechanism, we would expect higher, gapless identities in coding regions, given the fidelity required for adaptive advantage. Taken together, these results indicate that HGT is not the primary driver of whole-genome compositional convergence, which is more plausibly explained by environmental selection pressures revealed through our k-mer analysis.

L Analysis of impact of distance thresholds

In this section, we investigated the effect of varying the distance thresholds on the list of confirmed candidate pairs. Specifically, we adjusted the threshold from the 90th percentile to the 75th and 95th percentiles for each distance metric. The results are presented in Table S26.

Table S26: Effect of distance thresholds on the number of confirmed candidate pairs.

**Threshold**	**Value**			**# Confirmed**	**# Passing**
	**Distance Descriptor**	**DSSIM**	**LPIPS**	**Candidate Pairs**	**Hypothesis Testing**
75th percentile	0.162334	0.497843	0.142033	28	10
95th percentile	0.19020	0.50130	0.17760	53	15

Our threshold sensitivity analysis revealed that increasing the threshold produced 12 additional confirmed candidate pairs; however, none of these satisfied the label-matching criteria during hypothesis testing. Conversely, lowering the threshold removed 13 confirmed candidate pairs, 10 of which did pass hypothesis testing. These results demonstrate that thresholds can be adjusted based on the underlying distance distributions of the dataset. In this study, since the genus-level distance is very restrictive, we ended up using the 90th percentile threshold.

M Analysis of impact of number of sub-fragments (n)

To analyze the effect of n (number of sub-fragments) on classification performance, we conducted taxonomic and environment-type classification under the bias mitigation scenario, using $k = 6$ and an SVM model. Genome proxy lengths ranged from 1,000 bp to 1,000 kbp, and n ranged from 10 to 100k. Table S27 and Table S28 show the results of this experiment for the *Temperature Dataset* and the *pH Dataset*, respectively.

Table S27: The accuracy of SVM classifiers trained on the *Temperature Dataset*, under the bias mitigation scenario, for two different label assignments, taxonomy and environment labelling, and $k = 6$, fragment lengths of 1000bp, 10kbp, 50kbp, 100 kbp, 250 kbp, 500 kbp, and 1000 kbp, and n ranging from 10 to 100k. The classification accuracy was determined through stratified 10-fold cross-validation, and the numbers in bold show the highest accuracy for the respective genome proxy length. For values of n where the sub-fragment length is shorter than the k-mer size (6), a "–" is shown since no valid k-mers can be formed.

**Genome proxy length**	**Class labelling type**	**Number of sub fragments (n)**								
		5	10	100	500	1,000	5,000	10,000	50,000	100,000
1,000 bp *	Temperature	41.69	42.32	39.09	-	-	-	-	-	-
	Taxonomy	86.40	86.08	79.67	-	-	-	-	-	-
10 kbp	Temperature	65.97	70.29	69.16	65.51	65.79	-	-	-	-
	Taxonomy	97.31	98.15	98.15	97.14	96.47	-	-	-	-
50 kbp	Temperature	71.76	72.78	72.54	71.96	72.89	72.52	-	-	-
	Taxonomy	99.16	98.99	98.99	98.99	99.16	98.99	-	-	-
100 kbp	Temperature	74.04	74.77	74.19	74.21	72.75	72.82	73.16	-	-
	Taxonomy	99.16	99.16	99.16	99.16	99.16	99.16	99.16	-	-
250 kbp	Temperature	72.32	74.29	75.00	74.84	73.61	75.00	73.89	-	-
	Taxonomy	99.16	99.16	99.16	99.15	99.15	99.15	99.15	-	-
500 kbp	Temperature	74.17	74.40	75.33	74.28	74.51	75.10	74.55	73.88	-
	Taxonomy	99.16	99.16	99.16	99.16	99.16	99.16	99.16	99.16	-
1,000 kbp	Temperature	73.94	75.08	74.19	75.22	74.69	74.96	74.82	75.60	75.08
	Taxonomy	99.16	99.16	99.16	99.16	99.16	99.16	99.16	99.16	99.16

* A fragment length of 1000 bp was included only in this specific experiment. In all other experiments in the main study, it was excluded because it is too short to adequately capture genomic information.

Table S28: The accuracy of SVM classifiers trained on the *pH Dataset*, under the bias mitigation scenario, for two different label assignments, taxonomy and environment category, and $k = 6$, fragment lengths of 1000 bp, 10kbp, 50kbp, 100 kbp, 250 kbp, 500 kbp, and 1000 kbp, and n ranging from 10 to 100k. The classification accuracy was determined through stratified 10-fold cross-validation, and the numbers in bold show the highest accuracy for the respective genome proxy length. For values of n where the sub-fragment length is shorter than the k-mer size (6), a "–" is shown since no valid k-mers can be formed.

**Genome proxy length**	**Class labelling type**	**Number of sub fragments (n)**								
		5	10	100	500	1,000	5,000	10,000	50,000	100,000
1,000 bp *	pH	73.74	69.32	69.85	-	-	-	-	-	-
	Taxonomy	89.21	91.90	77.86	-	-	-	-	-	-
10 kbp	pH	81.60	83.27	83.27	82.25	81.63	-	-	-	-
	Taxonomy	97.86	97.89	96.81	97.89	97.89	-	-	-	-
50 kbp	pH	82.27	82.72	84.38	81.66	85.46	82.77	-	-	-
	Taxonomy	98.42	98.42	98.42	98.42	98.42	97.89	-	-	-
100 kbp	pH	82.22	83.80	83.24	82.74	83.80	83.27	83.27	-	-
	Taxonomy	97.30	98.42	98.42	97.86	98.42	98.42	98.42	-	-
250 kbp	pH	81.60	83.77	81.63	82.71	81.63	83.77	83.24	-	-
	Taxonomy	98.94	98.42	98.42	98.42	98.42	98.42	98.42	-	-
500 kbp	pH	82.69	82.75	81.60	82.19	82.19	82.71	82.71	82.19	-
	Taxonomy	98.42	98.42	98.42	98.42	98.42	98.42	98.42	98.42	-
1,000 kbp	pH	82.19	82.19	82.71	82.19	82.71	82.19	82.19	82.71	82.19
	Taxonomy	98.42	97.87	98.42	98.42	98.42	98.42	98.42	98.42	98.42

* A fragment length of 1000 bp was included only in this specific experiment. In all other experiments in the main study, it was excluded because it is too short to adequately capture genomic information.

The results show that sub-fragment lengths as short as 10 bp are sufficient to capture taxonomic or environmental components, indicating that the underlying patterns are relatively short, often under 10 bp. However, this does not hold for very short genome proxy lengths (e.g., 1,000 bp), particularly for environmental classification, as there are not enough k-mers to capture the patterns. These findings suggest that retaining both taxonomic and environmental components requires a "sufficient" number of "short" genome sub-fragments. In our study, we observed that the "short" sub-fragments can be as small as 10 bp, provided that the number of sub-fragments is sufficiently large. Formally, this requirement can be expressed as

$$len(\text{genome\_proxy}) \geq 10{,}000 \ , \quad \frac{len(\text{genome\_proxy})}{n} \geq 10\text{bp} \tag{1}$$

Thus, the "sufficient" number of genome sub-fragments should be at least 1,000 to reliably preserve taxonomic and environmental signals.

References

[1] Lynd, L., Baskaran, S., and Casten, S. (2001) Salt Accumulation Resulting from Base Added for pH Control, and Not Ethanol, Limits Growth of Thermoanaerobacterium thermosaccharolyticum HG-8 at Elevated Feed Xylose Concentrations in Continuous Culture. *Biotechnol. Prog.,* **17**(1), 118–125.

[2] Matteuzzi, D., Hollaus, F., and Biavati, B. (1978) Proposal of Neotype for Clostridium thermohydrosulfuricum and the Merging of Clostridium tartarivorum with Clostridium thermosaccharolyticum. *Int. J. Syst. Bacteriol.,* **28**(4), 528–531.

[3] Itoh, T., Suzuki, K., and Sanchez, P. C. et al. (2003) Caldisphaera lagunensis gen. nov., sp. nov., a novel thermoacidophilic crenarchaeote isolated from a hot spring at Mt Maquiling, Philippines. *Int. J. Syst. Evol. Microbiol.,* **53**(4), 1149–1154.

[4] Takahata, Y., Nishijima, M., and Hoaki, T. et al. (2001) Thermotoga petrophila sp. nov. and Thermotoga naphthophila sp. nov., two hyperthermophilic bacteria from the Kubiki oil reservoir in Niigata, Japan.. *Int. J. Syst. Evol. Microbiol.,* **51**(5), 1901–1909.

[5] Slobodkina, G. B., Kolganova, T. V., and Querellou, J. et al. (2009) Geoglobus acetivorans sp. nov., an iron(III)-reducing archaeon from a deep-sea hydrothermal vent. *Int. J. Syst. Evol. Microbiol.,* **59**(11), 2880–2883.

[6] Clifton, C., Walters, C., and Simoneit, B. (1990) Hydrothermal petroleums from Yellowstone National Park, Wyoming, U.S.A.. *Appl. Geochem.,* **5**(1-2), 169–191.

[7] Huber, R., Eder, W., and Heldwein, S. et al. (1998) Thermocrinis ruber gen. nov., sp. nov., a Pink-Filament-Forming Hyperthermophilic Bacterium Isolated from Yellowstone National Park. *Appl. Environ. Microbiol.,* **64**(10), 3576–3583.

[8] Capaccioni, B., Tassi, F., and Vaselli, O. (2001) Organic and inorganic geochemistry of low temperature gas discharges at the Baia di Levante beach, Vulcano Island, Italy. *J. Volcanol. Geotherm. Res.,* **108**(1-4), 173–185.

[9] Fiala, G. and Stetter, K. O. (1986) Pyrococcus furiosus sp. nov. represents a novel genus of marine heterotrophic archaebacteria growing optimally at 100°C. *Arch. Microbiol.,* **145**(1), 56–61.

[10] Dominova, I. N., Kublanov, I. V., and Podosokorskaya, O. A. et al. (2013) Complete Genomic Sequence of " Thermofilum adornatus " Strain 1910b T , a Hyperthermophilic Anaerobic Organotrophic Crenarchaeon. *Genome Announc.,* **1**(5).

[11] Zayulina, K. S., Kochetkova, T. V., and Piunova, U. E. et al. (2020) Novel Hyperthermophilic Crenarchaeon Thermofilum adornatum sp. nov. Uses GH1, GH3, and Two Novel Glycosidases for Cellulose Hydrolysis. *Front. Microbiol.,* **10**.

[12] Zeng, X., Zhang, X., and Jiang, L. et al. (2013) Palaeococcus pacificus sp. nov., an archaeon from deep-sea hydrothermal sediment. *Int. J. Syst. Evol. Microbiol.,* **63**(Pt_6), 2155–2159.

[13] Huber, R., Stöhr, J., and Hohenhaus, S. et al. (1995) Thermococcus chitonophagus sp. nov., a novel, chitin-degrading, hyperthermophilic archaeum from a deep-sea hydrothermal vent environment. *Arch. Microbiol.,* **164**(4), 255–264.

[14] Bazylinski, D. A., Farrington, J. W., and Jannasch, H. W. (1988) Hydrocarbons in surface sediments from a Guaymas Basin hydrothermal vent site. *Org. Geochem.,* **12**(6), 547–558.

[15] Neuner, A., Jannasch, H. W., and Belkin, S. et al. (1990) Thermococcus litoralis sp. nov.: A new species of extremely thermophilic marine archaebacteria. *Arch. Microbiol.,* **153**(2), 205–207.

[16] Orlando, V., Franco, T., and Dario, T. et al. (2011) Submarine and Inland Gas Discharges from the Campi Flegrei (Southern Italy) and the Pozzuoli Bay: Geochemical Clues for a Common Hydrothermal-Magmatic Source. *Procedia Earth Planet. Sci.,* **4**, 57–73.

[17] Ravot, G., Magot, M., and Fardeau, M.-L. et al. (1995) Thermotoga elfii sp. nov., a Novel Thermophilic Bacterium from an African Oil-Producing Well. *Int. J. Syst. Bacteriol.,* **45**(2), 308–314.

[18] Cadillo-Quiroz, H., Bräuer, S. L., and Goodson, N. et al. (2014) Methanobacterium paludis sp. nov. and a novel strain of Methanobacterium lacus isolated from northern peatlands. *Int. J. Syst. Evol. Microbiol.,* **64**(Pt_5), 1473–1480.

[19] Zhilina, T. N. and Zavarzin, G. A. (1987) NOTES:Methanosarcina vacuolata sp. nov., a Vacuolated Methanosarcina. *Int. J. Syst. Bacteriol.,* **37**(3), 281–283.

[20] Chen, R.-W., Wang, K.-X., and Wang, F.-Z. et al. (2018) Rubrobacter indicoceani sp. nov., a new marine actinobacterium isolated from Indian Ocean sediment. *Int. J. Syst. Evol. Microbiol.,* **68**(11), 3487–3493.

[21] Dianou, D., Miyaki, T., and Asakawa, S. et al. (2001) Methanoculleus chikugoensis sp. nov., a novel methanogenic archaeon isolated from paddy field soil in Japan, and DNA-DNA hybridization among Methanoculleus species.. *International Journal of Systematic and Evolutionary Microbiology,* **51**(5), 1663–1669.

[22] Maus, I., Wibberg, D., and Stantscheff, R. et al. (2012) Complete Genome Sequence of the Hydrogenotrophic, Methanogenic Archaeon Methanoculleus bourgensis Strain MS2 T , Isolated from a Sewage Sludge Digester. *J. Bacteriol.,* **194**(19), 5487–5488.

[23] DOE Joint Genome Institute Taxon 2540341105: [Methanoculleus bourgensis]. `https://img.jgi.doe.gov/cgi-bin/m/main.cgi?section=TaxonDetail&page=taxonDetail&taxon_oid=2540341105` (2025) Accessed: 2025-05-14.

[24] Sakai, S., Ehara, M., and Tseng, I.-C. et al. (2012) Methanolinea mesophila sp. nov., a hydrogenotrophic methanogen isolated from rice field soil, and proposal of the archaeal family Methanoregulaceae fam. nov. within the order Methanomicrobiales. *Int. J. Syst. Evol. Microbiol.,* **62**(Pt_6), 1389–1395.

[25] Shimizu, S., Ueno, A., and Tamamura, S. et al. (2013) Methanoculleus horonobensis sp. nov., a methanogenic archaeon isolated from a deep diatomaceous shale formation. *Int. J. Syst. Evol. Microbiol.,* **63**(Pt_11), 4320–4323.

[26] Weng, C.-Y., Chen, S.-C., and Lai, M.-C. et al. (2015) Methanoculleus taiwanensis sp. nov., a methanogen isolated from deep marine sediment at the deformation front area near Taiwan. *Int. J. Syst. Evol. Microbiol.,* **65**(Pt_3), 1044–1049.

[27] Narihiro, T., Kusada, H., and Yoneda, Y. et al. (2016) Draft Genome Sequences ofMethanoculleus horonobensisStrain JCM 15517,Methanoculleus thermophilusStrain DSM 2373, andMethanofollis ethanolicusStrain JCM 15103, Hydrogenotrophic Methanogens Belonging to the FamilyMethanomicrobiaceae. *Genome Announc.,* **4**(2).

[28] Leibniz Institute DSMZ - German Collection of Microorganisms and Cell Cultures GmbH Methanoculleus thermophilus CR-1 - BacDive ID 7022. (2025) Accessed: 2025-05-14.

[29] Khan, M. F. and Patra, S. (2018) Deciphering the rationale behind specific codon usage pattern in extremophiles. *Sci. Rep.,* **8**(1).

[30] Goldstein, R. A. (2007) Amino-acid interactions in psychrophiles, mesophiles, thermophiles, and hyperthermophiles: Insights from the quasi-chemical approximation. *Protein Sci.,* **16**(9), 1887–1895.

[31] Panja, A. S., Maiti, S., and Bandyopadhyay, B. (2020) Protein stability governed by its structural plasticity is inferred by physicochemical factors and salt bridges. *Sci. Rep.,* **10**(1).

[32] Zeldovich, K. B., Berezovsky, I. N., and Shakhnovich, E. I. (2007) Protein and DNA Sequence Determinants of Thermophilic Adaptation. *PLoS Comput. Biol.,* **3**(1), 63–72.

[33] Bowman, J. S. and Deming, J. W. (2014) Alkane hydroxylase genes in psychrophile genomes and the potential for cold active catalysis. *BMC Genomics,* **15**(1), 1120.

[34] Saunders, N. F., Thomas, T., and Curmi, P. M. et al. (2003) Mechanisms of Thermal Adaptation Revealed From the Genomes of the AntarcticArchaea Methanogenium frigidumandMethanococcoides burtonii. *Genome Res.,* **13**(7), 1580–1588.

[35] De Farias, S. and Bonato, M. (2002) Preferred codons and amino acid couples in hyperthermophiles. *Genome Biol.,* **3**(8).

[36] Raymond-Bouchard, I., Goordial, J., and Zolotarov, Y. et al. (2018) Conserved genomic and amino acid traits of cold adaptation in subzero-growing Arctic permafrost bacteria. *FEMS Microbiol. Ecol.,* **94**(4).

[37] Carbone, A., Képès, F., and Zinovyev, A. (2004) Codon Bias Signatures, Organization of Microorganisms in Codon Space, and Lifestyle. *Mol. Biol. Evol.,* **22**(3), 547–561.

[38] Hait, S., Mallik, S., and Basu, S. et al. (2019) Finding the generalized molecular principles of protein thermal stability. *Proteins,* **88**(6), 788–808.

[39] Riley, M., Staley, J. T., and Danchin, A. et al. (2008) Genomics of an extreme psychrophile, Psychromonas ingrahamii. *BMC Genomics,* **9**(1), 210.

[40] Szilágyi, A. and Závodszky, P. (2000) Structural differences between mesophilic, moderately thermophilic and extremely thermophilic protein subunits: results of a comprehensive survey. *Structure,* **8**(5), 493–504.

[41] McKay, L. J., Dlakić, M., and Fields, M. W. et al. (2019) Co-occurring genomic capacity for anaerobic methane and dissimilatory sulfur metabolisms discovered in the Korarchaeota. *Nat. Microbiol.,* **4**(4), 614–622.

[42] Reysenbach, A.-L., St. John, E., and Meneghin, J. et al. (2020) Complex subsurface hydrothermal fluid mixing at a submarine arc volcano supports distinct and highly diverse microbial communities. *Proc. Natl. Acad. Sci. U. S. A.,* **117**(51), 32627–32638.

[43] Jungbluth, S. P., Grote, J., and Lin, H.-T. et al. (2012) Microbial diversity within basement fluids of the sediment-buried Juan de Fuca Ridge flank. *ISME J.,* **7**(1), 161–172.

[44] Kobayashi, H., Toyoda, R., and Miyamoto, H. et al. (2021) Analysis of a Methanogen and an Actinobacterium Dominating the Thermophilic Microbial Community of an Electromethanogenic Biocathode. *Archaea,* **2021**, 1–13.

[45] Qin, Q.-S., Feng, D.-S., and Liu, P.-F. et al. (2017) Metagenomic Characterization of *Candidatus* Smithella cisternae Strain M82_1, a Syntrophic Alkane-Degrading Bacteria, Enriched from the Shengli Oil Field. *Microbes Environ.,* **32**(3), 234–243.

[46] Stamps, B. W., Lyles, C. N., and Suflita, J. M. et al. (2016) Municipal Solid Waste Landfills Harbor Distinct Microbiomes. *Front. Microbiol.,* **7**.
